# The diversity and evolution of chelicerate hemocyanins

**DOI:** 10.1186/1471-2148-12-19

**Published:** 2012-02-14

**Authors:** Peter Rehm, Christian Pick, Janus Borner, Jürgen Markl, Thorsten Burmester

**Affiliations:** 1 Institute of Zoology and Zoological Museum, University of Hamburg, D-20146 Hamburg, Germany; 2 Institute of Zoology, Johannes Gutenberg University Mainz, D-55099 Mainz, Germany

## Abstract

**Background:**

Oxygen transport in the hemolymph of many arthropod species is facilitated by large copper-proteins referred to as hemocyanins. Arthropod hemocyanins are hexamers or oligomers of hexamers, which are characterized by a high O_2 _transport capacity and a high cooperativity, thereby enhancing O_2 _supply. Hemocyanin subunit sequences had been available from horseshoe crabs (Xiphosura) and various spiders (Araneae), but not from any other chelicerate taxon. To trace the evolution of hemocyanins and the emergence of the large hemocyanin oligomers, hemocyanin cDNA sequences were obtained from representatives of selected chelicerate classes.

**Results:**

Hemocyanin subunits from a sea spider, a scorpion, a whip scorpion and a whip spider were sequenced. Hemocyanin has been lost in Opiliones, Pseudoscorpiones, Solifugae and Acari, which may be explained by the evolution of trachea (i.e., taxon Apulmonata). Bayesian phylogenetic analysis was used to reconstruct the evolution of hemocyanin subunits and a relaxed molecular clock approach was applied to date the major events. While the sea spider has a simple hexameric hemocyanin, four distinct subunit types evolved before Xiphosura and Arachnida diverged around 470 Ma ago, suggesting the existence of a 4 × 6mer at that time. Subsequently, independent gene duplication events gave rise to the other distinct subunits in each of the 8 × 6mer hemocyanin of Xiphosura and the 4 × 6mer of Arachnida. The hemocyanin sequences were used to infer the evolutionary history of chelicerates. The phylogenetic trees support a basal position of Pycnogonida, a sister group relationship of Xiphosura and Arachnida, and a sister group relationship of the whip scorpions and the whip spiders.

**Conclusion:**

Formation of a complex hemocyanin oligomer commenced early in the evolution of euchelicerates. A 4 × 6mer hemocyanin consisting of seven subunit types is conserved in most arachnids since more than 400 Ma, although some entelegyne spiders display selective subunit loss and independent oligomerization. Hemocyanins also turned out to be a good marker to trace chelicerate evolution, which is, however, limited by the loss of hemocyanin in some taxa. The molecular clock calculations were in excellent agreement with the fossil record, also demonstrating the applicability of hemocyanins for such approach.

## Background

Hemocyanins are large copper-proteins that transport O_2 _in the hemolymph of many arthropods and mollusks [1-3]. However, the hemocyanins of these two phyla are structurally different and emerged independently [4]. Hemocyanins evolved early in the arthropod stem lineage from the phenoloxidases, which are O_2_-consuming enzymes involved in the melanin pathway [3]. Other members of the arthropod hemocyanin superfamily have lost the ability to bind copper and thus O_2_, and gave rise to the non-respiratory pseudo-hemocyanins (cryptocyanins) in decapod crustaceans and the hexamerins in hexapods, which serve as storage proteins [3,5,6].

Arthropod hemocyanins form hexamers or oligo-hexamers of identical or related subunits with a molecular mass of about 75 kDa [1,2]. In each subunit, O_2_-binding is mediated by two Cu^+ ^ions, which are coordinated by six histidine residues ("type III" copper binding site). Based on biochemical, immunochemical and molecular phylogenetic analyses, distinct hemocyanin subunit types have been identified in Chelicerata, Myriapoda, Crustacea and Hexapoda [3,7-12]. These subunits experienced an independent evolution within each of these taxa, with the exception of a more complex pattern within the Pancrustacea (Crustacea and Hexapoda) [13].

Within the chelicerates, biochemical analyses have demonstrated the presence of hemocyanins in Xiphosura (horseshoe crabs), Scorpiones, Uropygi (whip scorpions), Amblypygi (whip spiders), and Araneae (true spiders), but failed to identify these respiratory proteins in Pycnogonida (sea spiders; Pantopoda), Solifugae (sunspiders) and Acari (mites and ticks) [8,10,11]. Complete sets of hemocyanin subunit sequences are available from the tarantula *Eurypelma californicum *(= *Aphonopelma hentzi*) [14], the hunting spider *Cupiennius salei *[15], the golden orb web spider *Nephila inaurata *[16] and the horse shoe crab *Carcinoscorpius rotundicauda *(GenBank acc. nos. DQ090484-DQ090469). Chelicerate hemocyanins are composed of up to eight distinct subunit types and form either 1 × 6, 2 × 6, 4 × 6 or 8 × 6mers [10,11]. Each subunit type occupies a distinct position within the native oligomer [7,17-21]. The subunits have similar oxygen binding properties, but different physico-chemical characteristics [22,23] and evolutionary origins [3,14-16].

Xiphosurans (horseshoe crabs) have the largest hemocyanin molecules known, consisting of 48 (8 × 6) subunits and up to eight distinct subunits types in *Limulus polyphemus *(6 × subunit type I, 8 × II, 2 × IIA, 8 × IIIA, 8 × IIIB, 8 × IV, 4 × V, 4 × VI) [1,10,19,21]. Scorpiones, Amblypygi, Uropygi, and some Araneae have 4 × 6mer hemocyanins. The 4 × 6mer hemocyanin of the tarantula *E. californicum *is the best studied example and comprises seven distinct subunit types (4 × a, 2 × b, 2 × c, 4 × d, 4 × e, 4 × f, and 4 × g-type subunits) [7,10,11,23]. A similar subunit composition was found in many other Araneae, the Amblypygi and the Uropygi [8,10]. Among the Araneae, variations from this "standard" scheme have been found in the entelegyne spiders of the RTA-clade (RTA = "retrolateral tibial apophysis"). In this large taxon, 1 × 6mer and 2 × 6mer hemocyanins occur that consist only of g-type subunits, but have lost the other six subunit types (a through f) present in other Araneae [10,11,15]. Scorpion hemocyanins are composed of eight subunit types named 2, 3A, 3B, 3C, 4, 5A and 5B [24]. Immunological and structural studies have suggested the correspondence between the distinct araneaen, scorpion and *Limulus *hemocyanin subunits [10,11,17,25-27]. This indicates that the evolution of distinct subunit types preceded the separation of Xiphosura and Arachnida.

To understand the evolution of the complex oligomeric structures of chelicerate hemocyanins, we have obtained 16 novel cDNA sequences of hemocyanin subunits from a sea spider, a horseshoe crab, a whip scorpion and a whip spider. Together with the previously sequenced hemocyanins from horseshoe crabs, scorpions and spiders, and those assembled from expressed sequence tags (ESTs), 67 full length chelicerate subunit sequences are available. These data allow us *i*. to trace the hemocyanin subunit evolution, *ii*. to reconstruct the emergence of the hemocyanin oligomers, and *iii*. to infer chelicerate phylogeny and divergence times.

## Methods

### Sequencing of chelicerate hemocyanin cDNA

Full length coding sequences were available for the hemocyanins of the tarantula *E. californicum *[14], the mangrove horseshoe crab *C. rotundicauda*, the golden orb web spider *N. inaurata *[16] and the hunting spider *C. salei *[15] (Additional file 1). In addition, hemocyanin primary structures had been obtained by conventional amino acid sequencing from the horseshoe crab *Tachypleus tridentatus *(TtrHcA; see Additional file 1 for the abbreviations of the proteins) [28] and the scorpion *Androctonus australis *(AauHc6) [29].

4,062 ESTs were generated from total RNA of the sea spider *Endeis spinosa *(Pycnogonida) as described before [30]. Gene ontology assessment and BLAST searches identified six ESTs with significant similarities to arthropod hemocyanins. Assembly of the ESTs resulted in a single cDNA sequence. Two cDNA clones from the original library were selected for sequencing by a commercial service (GATC, Konstanz, Germany), which both yielded identical sequences (acc. no. FR865911).

A cDNA library form the horseshoe crab *L. polyphemus *(Xiphosura) total RNA was prepared and screened with specific anti-*Limulus*-hemocyanin antibodies [21]. Four complete hemocyanin cDNAs sequences were obtained by primer walking. The cDNAs were assigned after translation to distinct subunits on the basis of known N-termini [31], identifying subunits II, IIIa, IV and VI (acc. nos. AM260213-AM260216). An additional hemocyanin cDNA (coding for subunit IIIB) was identified from a set of ESTs [30] and the complete coding sequence was obtained by primer walking (acc. no. FR865912).

A CloneMiner (Invitrogen) cDNA library was constructed from total RNA of a female emperor scorpion *Pandinus imperator *(Scorpiones) and submitted to 454 pyrosequencing [32], resulting in 428,844 high-quality reads. Hemocyanin subunit sequences were deduced from the assembled contigs (acc. nos. FN424079-FN424086).

A single whip spider *Euphrynichus bacillifer *(Amblypygi) was purchased from a commercial pet supplier. A cDNA library was constructed employing the Mint Universal kit (Evrogen). 433,348 reads were obtained from the cDNA by 454 pyrosequencing and the hemocyanin sequences were deduced from the assembled contigs (acc. nos. FR865913-FR865920).

A single whip scorpion *Mastigoproctus giganteus *(Uropygi) was purchased from a commercial pet supplier. Total RNA was extracted and converted into a Mint Universal cDNA library, from which 481,905 reads were obtained by 454 pyrosequencing. Full length coding sequences of hemocyanin subunits a, d, e, f, and g, as well as partial sequences from subunits b and c were deduced from the assembled contigs. The missing fragments of subunits b and c were obtained by RT-RCR employing gene specific primers. The cDNA fragments were cloned into pGEM and sequenced. The final subunit sequences have been submitted to the databases under the accession numbers FR865920-FR865926.

### Sequence assembly and analyses

The web-based tools provided by the ExPASy Molecular Biology Server of the Swiss Institute of Bioinformatics (http://www.expasy.org) were used for cDNA translation and the analyses of amino acid sequences. A multiple sequence alignment of the amino acid sequences of all available arthropod hemocyanins and selected arthropod phenoloxidases was constructed employing MAFFT 6 [33] with the G-INS-i routine and the BLOSUM 45 matrix at http://mafft.cbrc.jp/alignment/server/. A complete list of sequences used in this study is provided in Additional file 1. For the phylogenetic inferences, signal peptides as well as the N- and C-terminal extensions of some phenoloxidases and hemocyanins were excluded from the multiple sequence alignment. The final alignment comprised 143 sequences and 912 positions (Additional file 2).

### Phylogenetic analyses

The most appropriate model of amino acid sequence evolution (WAG + Γ model; [34]) was selected with ProtTest [35] using the Akaike Information Criterion. Bayesian phylogenetic analysis was performed using MrBayes 3.1.2 [36]. We assumed the WAG model with a gamma distribution of substitution rates. Metropolis-coupled Markov chain Monte Carlo sampling was performed with one cold and three heated chains. Two independent runs were performed in parallel for 4.5 million generations until the average standard deviation of split frequencies was < 0.01. Starting trees were random and the trees were sampled every 100th generation. The program Tracer 1.4 (http://tree.bio.ed.ac.uk/software/tracer/) was used to examine log-likelihood plots and Markov chain Monte Carlo summaries for all parameters. Posterior probabilities were estimated on the final 35,000 trees (burnin = 10,000). Trees were displayed using the arthropod phenoloxidases as an outgroup [4].

### Molecular clock calculations

The program PhyloBayes 3.3 was used for molecular clock estimates [37], employing the MrBayes consensus tree as input. First three relaxed clock models, the lognormal autocorrelated clock model (LOG) [38], the Cox-Ingersoll-Ross process (CIR) [39] and uncorrelated gamma multipliers (UGM) [40] were compared by tenfold cross-validation with eight replicates, as specified in PhyloBayes 3.3. Rates across sites were modeled assuming a discrete gamma distribution with four categories. Divergence time priors were either uniform or modeled with a birth death process. Node ages were calculated using either hard constrains, which do not allow calibrated nodes to fall outside the calibration dates or soft bounds, which allows for divergence times outside the calibration interval [37]. For each of these settings rates across sites were modeled assuming a discrete gamma distribution with four categories and a Dirichlet process. All calculations were run for 50,000 (burnin 20,000) cycles.

The tree was calibrated with fossil constraints [41-43]. The maximum age for the separation of arthropod subphyla and thus the maximum age of the origin of the Chelicerata was set to the base of the Cambrian period 543 Ma ago (Ma). Stratigraphic information was obtained from http://www.fossilrecord.net[44]. Numerical ages derive from the "International Stratigraphic Chart" 2009 (http://www.stratigraphy.org) (Table 1).

**Table 1:** Calibration points used for relaxed Bayesian molecular clock analyses

Split	Bounds		Strata	Fossils	Reference
	Max	Min			
Euchelicerata-Pycnogonida	543	501	lower bound: first arthropods in the Early Cambrian upper bound: base of Upper Cambrian	*Rusophycus*; *Cambropycnogon klausmuelleri*	Benton 1993 [41], Crimes 1987 [45], Waloszek and Dunlop 2002 [46]

origin of Xiphosura		445	Late Ordovician	*Lunataspis aurora*	Rudkin et al. 2008 [47]

origin of Scorpiones		428	Silurian	*Allopalaeophonus caledonicus*	Dunlop 2010 [43]

Mygalomorphae-Araneaomorphae	382.7	240	lower bound: Grès à meules, upper Buntsandstein, Trias; upper bound: Givetian, middle Devonian	*Rosamygale grauvogeli;* *Attercopus fimbriunguis*	Selden and Gall 1992 [48], Selden et al. 2008 [49]

## Results

### Hemocyanin sequences

A putative hemocyanin of the sea spider *E. spinosa *was identified in the ESTs of this species [30]. The EspHc1 cDNA measures 2,104 bp and translates into a protein of 631 amino acids with a predicted molecular mass of 72.2 kDa (Additional file 3). Full length cDNAs of five hemocyanin subunits of the Atlantic horseshoe crab *L. polyphemus *were obtained (LpoHcII, LpoHcIIIa, LpoHcIIIb, LpoHcIV, LpoHcVI). The predicted *L. polyphemus *hemocyanin subunits measure between 624 and 638 amino acids, with molecular masses of 72.3-73.4 kDa (Additional file 3). Subunits I, IIa and V could not identified in the cDNA library [21] or in the ESTs [30]. Seven hemocyanin subunit are available from the mangrove horseshoe crab *C. rotundicauda*, which corresponds to the subunits I, II, IIIa, IIIb, IV, V, and VI (acc. nos. DQ090484-DQ090490; Additional file 3). Thus seven of the eight hemocyanin subunit types of Xiphosura could be included in our analyses. The nature of an eighth type identified exclusively in *L. polyphemus *(IIa) and its exact topological position within the 4 × 6mer remains unclear. Its N-terminal sequence resembles that of subunit IIIa and it may occupy a homologous position in some hexamers [21,27,31].

**Figure 1:**
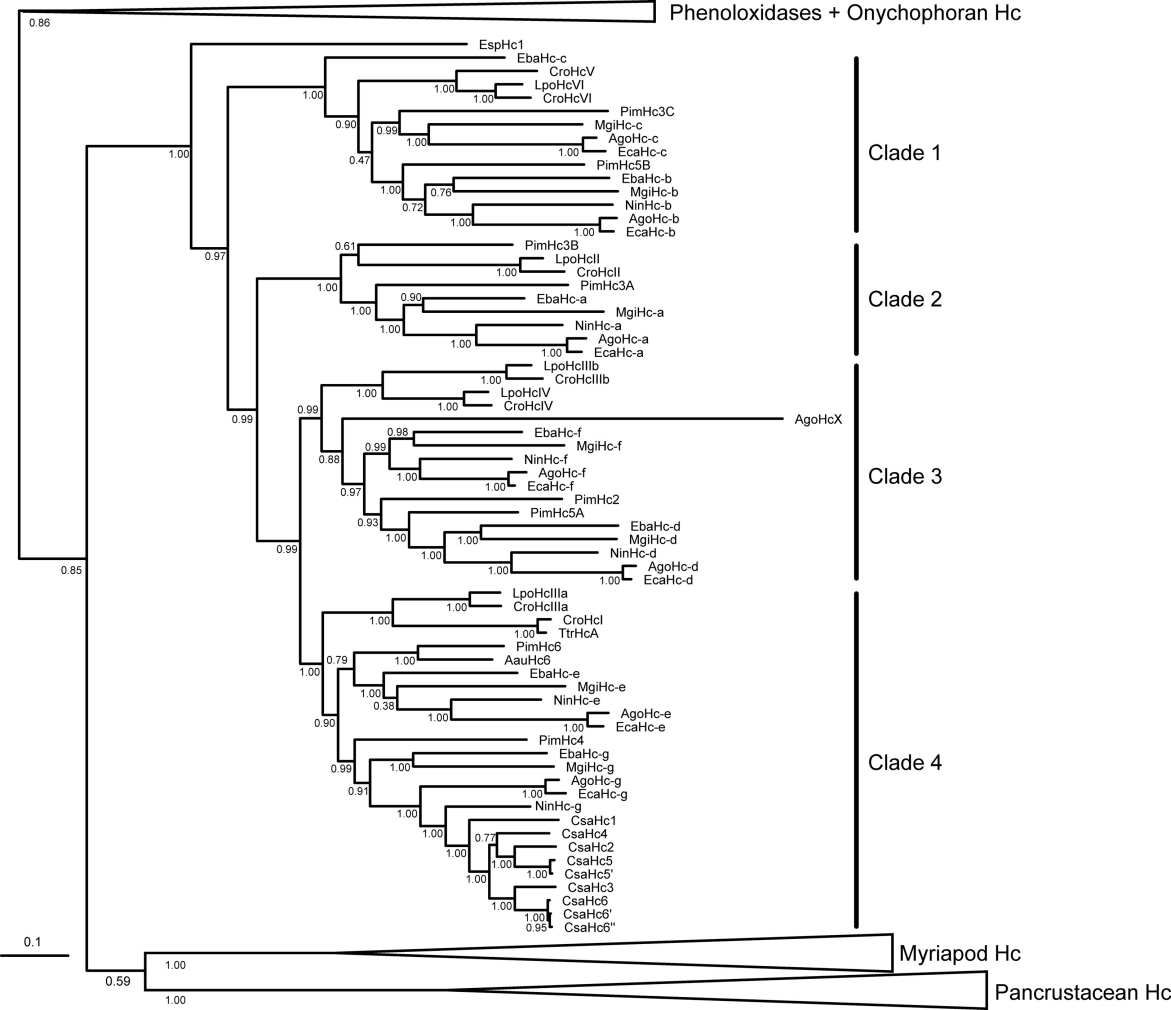
**Phylogenetic tree of the chelicerate hemocyanin subunits**. The numbers at the nodes represent Bayesian posterior probabilities estimated with the WAG model of amino acid substitution. The species abbreviations are: Aau, *Androctonus australis*; Ago, *Acanthoscurria gomesiana*; Cro, *Carcinoscorpius rotundicauda*; Csa, *Cupiennius salei*; Eba, *Euphrynichus bacillifer*; Eca, *Eurypelma californicum*; Esp, *Endeis spinosa*; Lpo, *Limulus polyphemus*; Mgi, *Mastigoproctus giganteus*; Nin, *Nephila inaurata*; Pim, *Pandinus imperator*; Ttr, *Tachypleus tridentatus*. The bar represents 0.1 expected substitutions per site. See Additional file 1 for abbreviations of the proteins

454 pyrosequencing was employed to obtain ESTs from the scorpion *P. imperator *[32], the whip spider *E. bacillifer *(unpublished), the whip scorpion *M. giganteus *(unpublished), the pseudoscorpion *Chelifer cancroides *(unpublished), the sun spider *Gluvia dorsalis *(unpublished) and the harvestman *Phalangium opilio *(unpublished). Hemocyanin sequences were identified in *E. bacillifer *and *M. giganteus*, but not in *P. opilio *(474,081 reads), *G. dorsalis *(425,934 reads) or *C. cancroides *(443,697 reads). In both, *E. bacillifer *and *M. giganteus *seven hemocyanin sequences were found, which are orthologous to *E. californicum *hemocyanin subunits a-g (Figure 1). The predicted proteins measure between 621 and 639 amino acids, with molecular masses of 71.2-73.7 kDa (Additional file 3).

The publicly available chelicerate ESTs were searched for hemocyanin sequences using the tblastn algorithm (4 November 2011). We identified hemocyanin sequences in the ESTs from the tarantula *Acanthoscurria gomesiana *(see below), the hunting spider *C. salei *(two ESTs), the orb web spider *Nephila antipodiana *(two ESTs) and the tarantula *Aphonopelma *sp. (one EST). A total of 173 ESTs from the house spider *Parasteatoda tepidariorum *display significant similarities to hemocyanin and represent all seven subunits (a-g) found in other Araneae. However, none of the ESTs from *C. salei, N. antipodiana, Aphonopelma *sp. or *P. tepidariorum *could be assembled to a complete coding sequence and therefore these sequences were not included in our analyses. Lorenzini and colleagues obtained 6,790 ESTs from a hemocyte library of *A. gomesiana *[50]. A total of 463 ESTs display significant similarities with hemocyanin, as identified by BLAST searches. These sequences were assembled into eight contigs. On the basis of similarity searches, seven subunits were assigned to arachnid hemocyanin subunits a-g (Additional file 3). The eighth putative hemocyanin sequence (tentatively named AgoHcX) has no obvious ortholog among the chelicerate hemocyanin subunits.

### Phylogeny of chelicerate hemocyanin subunits

Bayesian phylogenetic analyses show that the hemocyanins tree basically follows the accepted arthropod relationships on the level of the subphyla (Figure 1). The euarthropods split in the two sister groups Chelicerata and Mandibulata; though, the clade comprising the hemocyanins of the Mandibulata (Myriapoda + Pancrustacea) is poorly supported (Bayesian posterior probability 0.58). Myriapod and pancrustacean hemocyanins each are monophyletic, whereas the crustacean hemocyanins are not because the remipede hemocyanins were found more closely related to those of the insects than to the malacostracan hemocyanins [13].

Chelicerate hemocyanins are monophyletic (Bayesian posterior probability = 1.0). The lineage leading to the hemocyanin subunit from the sea spider *E. spinosa *(EspHc1) diverges first. Within the euchelicerate hemocyanins, four well-supported clades of distinct subunit types were identified (clades 1-4; Bayesian posterior probabilities ≥ 0.99). Clade 1 is the first branch within the euchelicerate hemocyanins and is formed by the arachnid b/c-type and xiphosuran V/VI-type subunits. These subunits facilitate the inter-hexamer contacts within the 4 × 6mer, as well as between the 2 × 6mer half-structures [1,21]. While the xiphosuran subunits V and VI form a common monophyletic clade, the arachnid hemocyanin subunits c do not. Here, the sequence of the putative c-subunit of the whip spider *E. bacillifer *(EbaHc-c) was found more diverged, mirrored by its basal position within the b/c clade.

Clade 2 comprises the arachnid a-type and the xiphosuran type II subunits. In agreement with immunological studies with the scorpion *A. australis *[17], the scorpion *P. imperator *has two a-type subunits, of which PimHc3A groups with a basal position to the other arachnid a-type subunits, while the position of PimHc3B is not well resolved. Note that subunit 3B represents a unique feature of scorpions that does not occur in the other arachnid hemocyanins.

The common clade that includes the remaining euchelicerate subunits received 0.99 Bayesian support. This clade splits into two sub-clades, leading to arachnid subunits d and f, and xiphosuran subunits IIIb and IV, on the one hand (clade 3), and arachnid subunits e and g, and xiphosuran subunits I and IIIa, on the other (clade 4). Within the arachnid d/f and e/g subtrees, respectively, the observed subunit relationships essentially mirror the expected phylogeny of the species, although the positions of the scorpion subunits are somewhat ambiguous (notably PimHc2). The HcX sequence, which had only been identified in *A. gomesiana*, is included in the d/f-clade, a position that is confirmed by pairwise comparisons, revealing that AgoHcX displays the highest sequence similarity to the arachnid f-subunits (not shown). Exclusion of AgoHcX from the phylogenetic analyses results in the same topology as described here, but Bayesian support slightly increased throughout the tree (Additional file 4).

Entelegyne spiders of the RTA-clade, such as *C. salei*, are known to diverge from the arachnid standard scheme of 4 × 6mer hemocyanins and to possess a mixture of 1 × 6mers and 2 × 6mers [10,11,18]. In agreement with previous studies [15], we found that the *C. salei *hemocyanin subunits all belong to the arachnid g-type, whereas the other six types (a-f) appear to have been lost during the evolution of this taxon. The *C. salei *hemocyanin subunits consistently group with the subunit g of *N. inaurata*, an entelegyne spider with a 4 × 6mer hemocyanin.

### Molecular clock analyses of chelicerate hemocyanins

A timescale of chelicerate hemocyanin evolution was inferred on the basis of the chelicerate hemocyanins described above. Due to the divergent evolutionary rates (cf. Figure 1), we excluded the hemocyanin sequences of Onychophora, Myriapoda, Crustacea and Hexapoda, as well as the phenoloxidases. Cross-validation shows that that the uncorrelated gamma clock model (UGM) fits better than the log-normal model or the CIR process. The cross-validation score of UGM vs. log-normal was 6.4625 +/- 18.8999 and of UGM vs. CIR 5.8125 +/- 21.1925. Further support comes from a comparison of the calculated divergence times of different orthologous subunit pairs (e.g., EcaHc-a vs. NinHc-a compared to EcaHc-d vs. NinHc-d), which are most similar under UGM. Thus UGM was applied in our calculations. The time estimates with soft bounds and hard bounds were similar, with estimates being on average ~5% older when using hard bounds (Additional file 5 and Additional file 6). No notable difference was observed between the results obtained with rates modeled with the Dirichlet process or with a discrete gamma distribution (< 0.1% mean difference).

**Figure 2:**
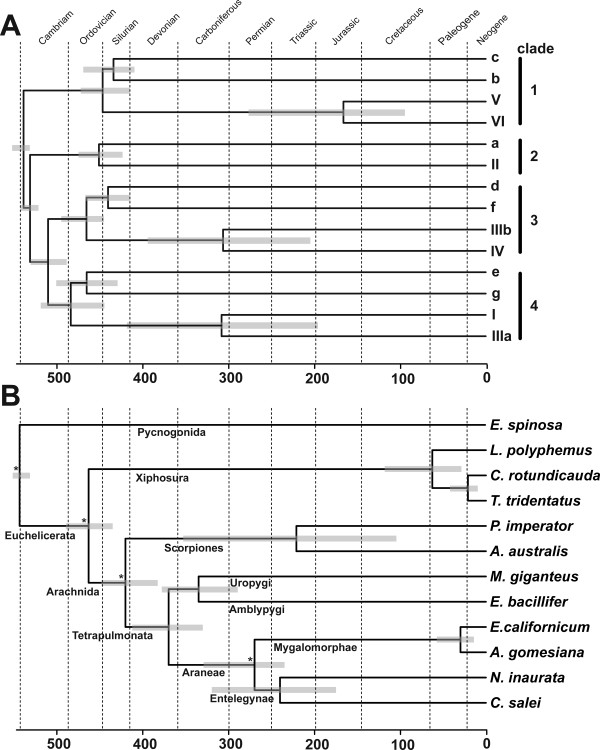
**Timescale of hemocyanin evolution**. **A**. Evolution in the chelicerate hemocyanin subunits. **B**. Hemocyanin-derived timescale of chelicerate evolution. The divergence times are means resulting from the estimates obtained with a birth-death process and soft bounds. Rates across sites were modeled assuming a gamma distribution. The grey bars correspond to the 95% confidence intervals. Ma, million years ago; asterisks denote the nodes used for calibration (see Table 1).

The divergence times resulting from the estimates obtained with soft bounds, a birth death process and rates modeled with the Gamma distribution are displayed in Figure 2 (see Additional file 5). We applied five calibration points, which correspond to four upper (minimum) and two lower (maximum) bounds derived from the fossil record (Table 1). Subunits with uncertain orthology were ignored. We first calculated the divergence times of the distinct subunit types in Arachnida and Xiphosura (Figure 2A). The earliest split of euchelicerate hemocyanins is formed by the clade of arachnid b/c and xiphosuran V/VI-type subunits, which occurred ~540 Ma. Within this clade, the exact arrangement of the three clades consisting of *i*. arachnid b-type subunits, *ii*. arachnid c-type subunits and xiphosuran V/VI-type subunits is not well resolved (Figure 1), which is reflected by a rapid diversification 437-453 Ma. Xiphosuran subunits V and VI separated ~169 Ma. The a-type subunits, which include the xiphosuran subunit II, split ~536 Ma, followed by the four clades defined above, consisting of arachnid subunits d/f, arachnid e/g, xiphosuran I/IIIa, and xiphosuran IIIb/IV subunits, which commenced to diversify ~509 Ma. Arachnid d- and f-type subunits split ~441 Ma, arachnid e- and g-type subunits ~467 Ma. Separation events within the xiphosuran subunits (I vs. IIIa and IIIb vs. IV) occurred 304 and 309 Ma, respectively. In subsequent analyses, the hemocyanin sequences were employed to estimate chelicerate divergence times (Figure 2B, see below).

### Hemocyanin-derived phylogeny of Chelicerata

We specifically studied the relationships among chelicerate taxa by concatenating the seven orthologous hemocyanin subunit sequences, representing spider subunits a-g, into a single sequence alignment. Clear orthologs were available for *E. californicum, A. gomesiana, N. inaurata, E. bacillifer*, and *M. giganteus*, respectively. There is no c-type subunit in *N. inaurata*, which was coded as missing data. *P. imperator *has two a-type subunits (PimHcIIIA and PimHcIIIB), of which we selected PimHcIIIA on the basis of its closer relationship to araneaen a-type subunits. In case of *C. rotundicauda*, clear orthology assessment was only possible for subunit a (see above). We assigned CroHcV and VI to b and c, CroHcIIIb and IV to d and f, and CroHcI and IIIa to e and g-subunits. In a second approach, we exchanged these sequences. Because the single *E. spinosa *hemocyanin subunit constitutes a conclusive outgroup for all seven subunit types (Figure 1), seven copies of this sequence were concatenated.

Bayesian phylogenetic analyses were applied and the sea spider *E. spinosa *was used as outgroup (Figure 3.3). All nodes displayed a Bayesian support of 1.0 and there was no effect of an exchange of the ambiguous subunits from *C. rotundicauda*. The Xiphosura (*C. rotundicauda*) are the sister taxon of the Arachnida. Within the Arachnida, the scorpion *P. imperator *diverged first. Whip spiders (Amblypygi) and whip scorpions (Uropygi) form a common clade (Pedipalpi). The Pedipalpi form the sister taxon of the Araneae, represented by the mygalomorph spiders *E. californicum *and *A. gomesiana *on the one hand, and the entelegyne spider *N. inaurata *on the other.

**Figure 3:**
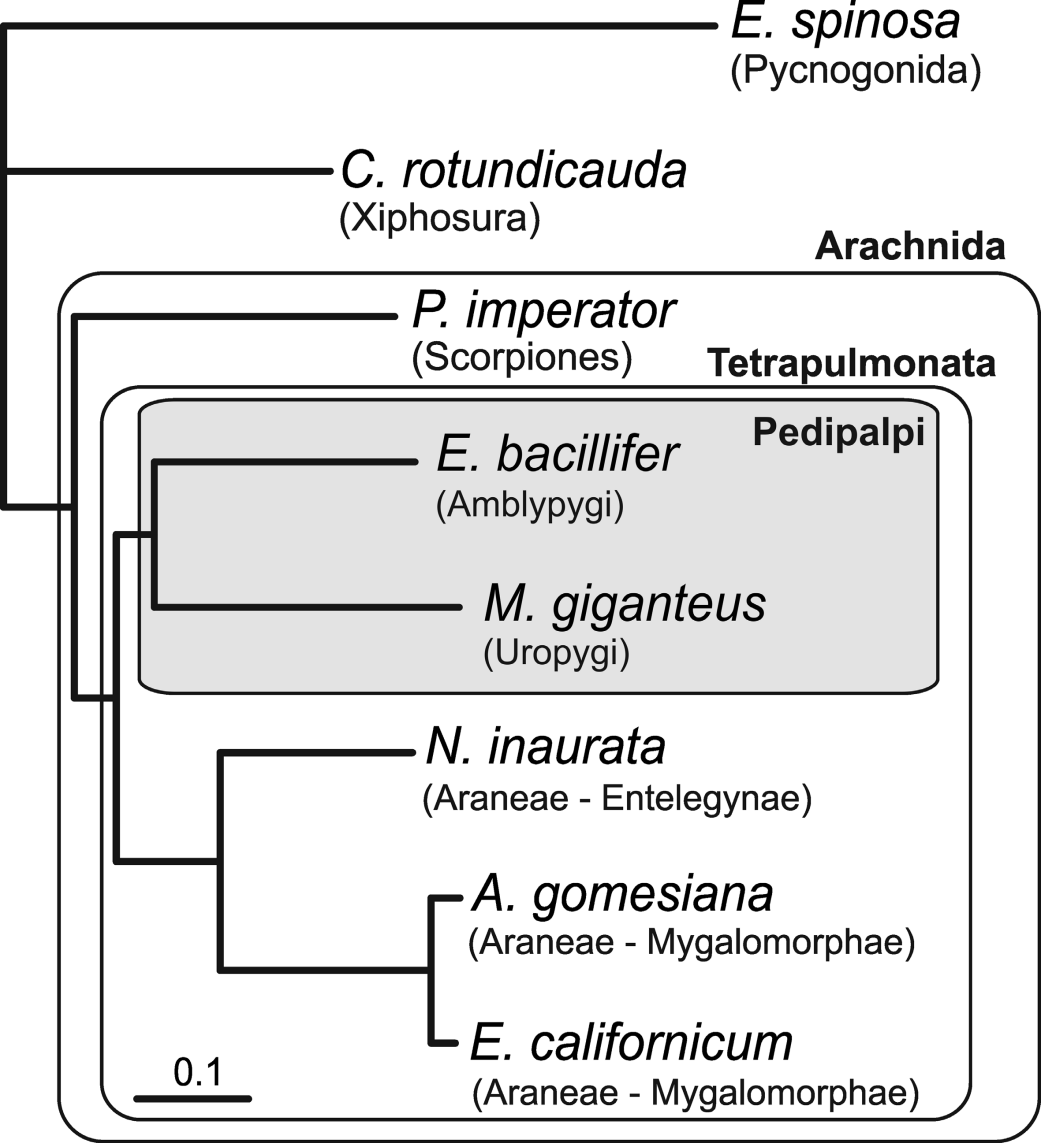
**Hemocyanin-based phylogeny of chelicerates**. The tree was derived from the concatenated hemocyanin alignment. The Pedipalpi are shaded. All nodes are supported with 1.0 Bayesian probabilities. The bar represents 0.1 substitutions per site.

According to the molecular clock calculations (see above), the hemocyanins of Pycnogonida and Euchelicerata diverged ~543 Ma (Figure 2B). Considering the different orthologous subunits, xiphosuran and arachnid hemocyanins separated between 444 to 489 Ma (mean 462 Ma). We calculated that the orthologous hemocyanin subunits of Scorpiones and Tetrapulmonata (i.e. Araneae + Pedipalpi) split ~419 Ma (405-440 Ma). Within the scorpions, *P. imperator *(Iurida) and *A. australis *(Buthida) separated ~221 Ma. The hemocyanins of Pedipalpi and Araneae diverged ~369 Ma (357-414 Ma), those of Amblypygi and Uropygi 334 Ma (316-344 Ma). Within the Araneae, hemocyanins of *N. inaurata *(Entelegynae) and the Mygalomorphae (*E. californicum *+ *A. gomesiana*) diverged ~271 Ma (254-288 Ma). The hemocyanins of *E. californicum *and *A. gomesiana *split 30 Ma (21-35 Ma). *N. inaurata *subunit g and the *C. salei *hemocyanins separated ~239 Ma; the xiphosurans *L. polyphemus *and *C. rotundicauda *diverged ~62 Ma (56-67 Ma).

## Discussion

Hemocyanin subunits assemble into hexamers, which may form quaternary structures comprising up to 8 × 6mers [1]. The evolutionary advantage of large oligomers presumably lies in a higher O_2_-carrying capacity per mol and higher cooperativity, which also enhances the O_2 _transport, combined with a low viscosity and a low colloid-osmotic pressure of the hemolymph. The phylogenetic tree permits inferring the origins and modifications of these complex protein structures in the chelicerates.

**Figure 4:**
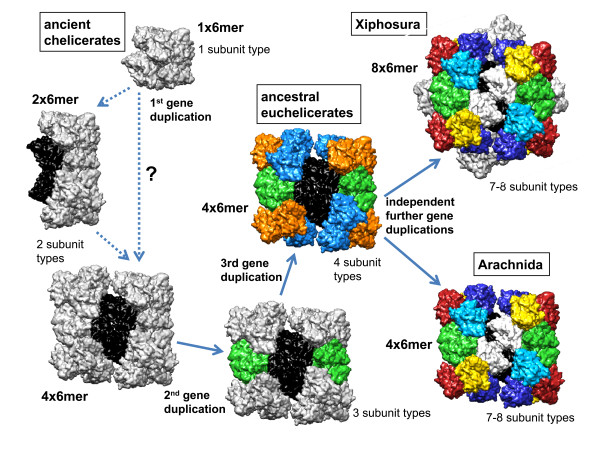
**Scheme of hemocyanin evolution in Chelicerata**. Color code: black/white, subunit clade 1 (b/c/V/VI); green, subunit clade 2 (a/II); medium blue, subunit clade 3 (d/f/IIIb/IV); orange, subunit clade 4 (e/g/I/IIIa); light blue, d/IV; dark blue f/IIIb^Q3^; yellow, g/IIIa; red, e/I. See text for further details and explanations.

The presence of only a single subunit in *E. spinosa *along with its basal position in the tree suggests that early chelicerate hemocyanins had a simple, homo-hexameric structure (Figure 4). This hypothesis is supported by the independent emergence of hemocyanin oligo-hexamers in the other arthropod subphyla, which hints to more simple hemocyanins in the last common arthropod ancestor [1,3].

### Early emergence of euchelicerate hemocyanin oligomers

The phylogenetic analyses demonstrate that the early euchelicerate hemocyanin, which was already used for O_2 _supply in the last common ancestor of the arachnids and the xiphosurans more than 445 Ma, was composed of at least four distinct subunit types. These subunits were the ancestors of the subunits represented in clades 1-4, respectively (Figure 1 and 2A). According to our phylogenetic reconstruction, clade 1, which includes arachnid b/c and xiphosuran V/VI subunits, diverged first. Both b/c and V/VI-subunits form heterodimers, which are responsible for the contacts between the hexamers [1,7,17,21,31]. Thus the emergence of clade 1-subunits was most likely associated with the organization of the first oligo-hexameric hemocyanin (Figure 4). This event must have taken place very early in the evolution of euchelicerates, and may be associated with significant morphological and physiological changes. We calculated that the corresponding gene duplication occurred ~540 Ma (Figure 2A). It may be speculated that first a 2 × 6mer consisting of two distinct subunit types evolved. Such dodecamers occur today in various crustaceans [1,10] and in the spiders of the RTA-clade [15,18]. Alternatively, this gene duplication has already resulted in a typical chelicerate 4 × 6mer, which is stabilized exclusively by a central tetrameric ring of clade 1 subunits. Notably, stable 4 × 6mers were obtained in hybrid reassembly experiments from mixtures of a clade 1 heterodimer as "linker" and another subunit type as "hexamer former" [51]. These experiments convincingly worked with scorpion heterodimer 5B-3C plus *L. polyphemus *subunit II, tarantula heterodimer b-c plus scorpion subunit 4, and *Limulus *heterodimer V-VI plus scorpion subunit 4.

The next step in evolution was the separation of clade 2 from the remaining subunits ~536 Ma. Clade 2 includes arachnid subunit a and xiphosuran subunit II. With the exception of PimHc3B (which exclusively occurs in scorpions), the a-subunits follow the expected phylogeny of the euchelicerates. In the hemocyanin quaternary structure, subunit a/II is located at the inter-hexamer interface of the basic 2 × 6mer [21]. It may therefore be speculated that this step was required not only for stabilization of the 4 × 6mer hemocyanin, but also for improving cooperativity (Figure 4).

An additional gene duplication event, which may have taken place ~510 Ma, long before the arachnid-xiphosuran split, gave rise to both, clade 3, consisting of arachnid subunits d and f, and xiphosuran subunits IIIb and IV, and clade 4, comprising arachnid subunits e and g, and xiphosuran subunits I and IIIa. Even though this largely is in accordance with previous comparative immunochemical studies [10,11,25,27], a common origin of subunits e and I, d and IV, f and IIIb, and IIIa and g, respectively, as proposed before [10,26], can now be excluded.

The separation of the arachnid subunits d and f, and e and g, respectively, occurred before scorpions and spiders diverged. Thus the last common ancestor of all arachnids had a 4 × 6mer of seven subunits that were similar to subunit types a-g, demonstrating the evolutionary success of this conserved structure, which has remained essentially unchanged for more than 450 Ma.

### Independent but parallel evolution of hemocyanin oligomers in Xiphosura and Arachnida

In horseshoe crabs, a large 8 × 6mer evolved. Although cooperativity is not further enhanced by this step, it might have been required for reducing the osmotic pressure and the viscosity of the hemolymph [1]. Notably, the duplication of the subunits corresponding to arachnid b and c (i.e., V and VI), d and f (IIIb and IV) and e and g (I and IIIa) occurred independently and the separation of xiphosuran subunits occurred more than 150 Ma later. Nevertheless, a comparable subunit diversity evolved: Six distinct subunit types for each topological position within the hexamer, plus a heterogeneity of the central linker unit to form an asymmetric 2 × 6mer. Consequently, today both arachnid and xiphosuran hemocyanins consist of seven distinct subunits (plus an independently evolved eighth subunit in *L. polyphemus *[IIa] and the scorpions [3B]), but only subunits a and II (clade 2) are one-to-one-orthologs. Thus, there was an evolutionary pressure to maximize the distinctiveness of subunits within the hemocyanin hexamer, which may be explained by better regulatory properties.

### Evolution of the arachnid hemocyanins

As outlined above, the principal structure of the early arachnid hemocyanin was most likely a 4 × 6mer (Figure 4). Previous immunological and structural investigations identified orthologs between scorpions and spiders (Araneae) [10,11,17,24,25,27]. The phylogenetic tree (Figure 1) shows that these studies were essentially correct. We should also note that in our previous phylogenetic analyses, the subunit AauHc6 was assigned to the araneaen g-type subunits [16], while on the basis of the structural and immunological similarities AauHc6 was homologized with e-type subunits [29]. The new tree, which includes more sequences, suggests that the protein-based studies were correct and AauHc6 is indeed an e-type subunit.

The 4 × 6mer structure is present in the mygalomorph spiders (*E. californicum *and *A. gomesiana*), and is found also in many Entelegynae (eight-eyed spiders; e.g., *N. inaurata*) [10,14,16]. The entelegyne spiders of the RTA-clade, however, diverge from this standard scheme and have a mixture of 1 × 6mer and 2 × 6mer hemocyanins [10,15,18,25]. This hemocyanin type is built by six distinct g-type subunits, with subunit CsaHc1 (see Figure 1) forming the inter-hexamer bridge within the 2 × 6mer molecules [15]. This suggests a loss of the other six subunit types (a-f) during evolution. Thus the ancestor of RTA-clade spiders most likely had a simple hexameric hemocyanin, exclusively built by g-type subunits. Some 170 Ma, the reconstruction of a more complex hemocyanin type commenced. This might be explained by physiological and behavioral changes that e.g. required a higher oxygen capacity in the hemolymph and/or a hemocyanin with a higher cooperativity.

There is little information about the subunit AgoHcX, which is only known from the ESTs of *A. gomesiana *[50]. Phylogenetic analyses place the protein with a long branch at the base of the arachnid d/f-subunit clade. The fact that the AgoHcX sequence was found in ESTs and does not display any nonsense mutation suggests that this unique hemocyanin-like protein is translated into a functional protein. However, it is neither known whether HcX is restricted to certain taxa (e.g., the mygalomorph spiders) nor whether it is component of the hemocyanin oligomer. The latter seems to be unlikely because of its derived sequence. In addition, no evidence for HcX was found in the hemocyanin of the closely related tarantula *E. californicum*, despite more than 30 years of research.

### Absence of hemocyanin in some chelicerate taxa

Notably, some arachnids do not have hemocyanin or any other O_2_-transport protein in their hemolymph. Despite the large number of ESTs obtained, no hemocyanin sequences were detected in the harvestman *P. opilio *(Opiliones), the pseudoscorpion *C. cancroides *(Pseudoscorpiones) and the sun spider *G. dorsalis *(Solifugae). This observation essentially agrees with previous findings [10,11,26], with the exception that Kempter et al. [26] suggested the presence of a dodecameric hemocyanin in the harvestman *Leiobunum limbatum*. However, re-evaluation of the original data and new experiments suggest that the protein in question may actually be a vitellogenin-like, di-tetrameric protein (not shown), similar to those found in other arachnids [8]. In this context, we would also like to note that in contrast to previous suggestions [10], the haplogyne spider *Dysdera *does not possess a 1 × 6mer hemocyanin. A recent reinvestigation of a number of individuals demonstrated that *Dysdera *lacks any hemocyanin, but express a tetrameric non-respiratory protein (not shown). We formally cannot exclude that hemocyanins are present in other species of these taxa or are expressed only under certain environmental conditions. However, we consider such scenario unlikely because such specific expression is not observed in other chelicerates. In addition, no evidence for hemocyanin was found in the 394,960 ESTs or the available genomic sequences of Acari. Thus mites and ticks most likely lack hemocyanin as well.

Opiliones, Pseudoscorpiones and Solifugae are apulmonate arachnids. The absence of hemocyanin may be a synapomorphic character and an indication for a close relationship of these taxa, which agrees with some character-based phylogenetic studies [52,53] (see below). Morphological and/or physiological characteristics may have rendered a respiratory protein unnecessary. Notably, apulmonate arachnids do not breathe through book lungs, but possess trachea, which may be sufficient to support the aerobic metabolism. The hemocyanin-less Acari (mites and ticks) are usually small and also have trachea [54]. By contrast, spiders, scorpions, whip spiders, whip scorpions have book lungs, which are filled with hemolymph and which may limit O_2 _consumption. Here, hemocyanin may be required for efficient O_2 _uptake and distribution.

### Implications for chelicerate phylogeny

Hemocyanin sequences have been successfully used to infer arthropod phylogeny [3,4,12,15,16,55-57]. Traditionally, Chelicerata were considered as the sister group of the Mandibulata, a taxon that comprises Myriapoda, Crustacea and Hexapoda [58]. However, several molecular phylogenetic studies have provided evidence for a common clade of Myriapoda and Chelicerata ("Myriochelata" or "Paradoxopoda" hypothesis; e.g., [59-62]). Morphological evidence is poor and restricted to similarities of neurogenesis [63], which may, however, also represent a plesiomorphic state. In our study, we received some support for the monophyly of Mandibulata, which agrees with previous studies employing hemocyanin sequences [9], as well as other molecular approaches [64,65].

The relationship among the major chelicerate lineages is controversial. Phylogenetic trees derived from the hemocyanin subunit sequences (Figure 1) or the concatenated alignment (Figure 3) can also be used to deduce the relative position of some chelicerate taxa, while others cannot be considered due to the lack of hemocyanin (Acari, Opiliones, Pseudoscorpiones, Solifugae). Notably, Weygoldt and Paulus [52] suggested a taxon "Apulmonata", which joins Solifugae, Opiliones, Pseudoscorpiones, Acari, Ricinulei (hooded tickspiders), and Palpigradi (microwhip scorpions). Palpigradi and Ricinulei were not available for our studies. However, the absence of hemocyanin in Acari, Opiliones, Pseudoscorpiones and Solifugae tentatively supports monophyletic "Apulmonata".

Morphological and molecular studies have placed the pycnogonids (sea spiders) either as sister group of the Euchelicerata [59,66], nested within the Chelicerata [67], or considered them as the sister group of all other Euarthropoda ("Cormogonida" hypothesis; [68]). Our phylogenetic tree (Figure 1) strongly supports the inclusion of the Pycnogonida in the Chelicerata as sister group of the Euchelicerata. This position is also tentatively supported by the hemocyanin mono-hexamer and is in line with recent neuroanatomical studies, which demonstrated the homology of deuterocerebral appendages of Pycnogonida and Euchelicerata [69]. An ingroup position of the Pycnogonida with the Arachnida, as deduced from complete mitochondrial DNA sequences [70,71] is not supported by our data and should be considered unlikely.

In agreement with morphological considerations and most previous molecular phylogenetic studies, the Xiphosura form the sister group of the Arachnida. Within the arachnids, the relative positions of Scorpiones, Araneae, Uropygi and Amblypygi are controversial [66]. We found monophyletic Tetrapulmonata (Araneae, Uropygi, and Amblypygi), which is the sister group of the scorpions. In previous phylogenetic analyses, the relative position of the taxa Araneae, Amblypygi and Uropygi has been controversial. While some morphological studies favor a sister group relationship between Araneae and Amblypygi, forming the taxon Labellata [66,72], others support a common taxon referred to as Pedipalpi, which comprises the Uropygi and Amblypygi [73]. The latter view is supported by our molecular phylogenetic trees. This finding holds for the tree derived from the concatenated alignment (Figure 3) as well as for most analyses of single subunits (Figure 1), with the exception of an unusual position of *E. bacillifer *subunit c and unresolved relationships among the subunits e.

The fossil record of chelicerates is far from being complete, but still allows the estimation of the evolutionary history of this taxon [42,43]. The true origin of the chelicerates is currently uncertain, but dates back at least to the early Cambrian period [43]. We calculated that the first split within the chelicerates occurred 542 Ma (Figure 2B). This slightly predates the earliest stemline chelicerates, which derive from the Lower Cambrian Maotianshan Shale some 530 Ma [74]. The first putative pycnogonid derives from the Orsten fauna ~500 Ma [46], the oldest xiphosuran fossil was found in a Late Ordovician Lagerstätte and dates ~445 Ma [47] and the first unambiguous arachnid is a ~428 Ma old Silurian scorpion [42,43]. These dates are actually close to our molecular clock estimates (463 and 420 Ma, respectively; Figure 2B). The first fossils of true spiders, whip spiders and whip scorpions were found in Carboniferous strata, which are 310-320 Ma old, although the Tetrapulmonata are probably of Devonian origin [43]. Our calculations confirm this notion and date the origin of the clade leading to Tetrapulmonata 369 Ma. The oldest opisthothele fossil (modern spiders) is a mygalomorph spider dating 240 Ma, while the oldest representative of the sistergroup Araneomorphae (web-building spiders) is of early Cretaceous origin [43]. We calculated the earliest divergence within the Araneae 271 Ma, which is somewhat older.

The most successful subgroup within the Araneomorphae are the Entelegynae, which are subdivided into the Orbicularidae and the spiders of the RTA clade. The lower bound of divergence of the Orbicularidae (e.g., *N. inaurata*) and the RTA-clade (*C. salei*) is a net from an orbicularian spider from the early Cretaceous period, some 140 Ma. We calculated that the formation of the *Cupiennius*-type hemocyanin commenced about 171 Ma, which should be considered as the lower bound for the time of emergence of the RTA-clade.

## Conclusions

Our results clearly demonstrate that chelicerate hemocyanin structure is conservative, but also allows innovations. There is little doubt that hemocyanin evolution commenced as a hexamer with a single subunit type, as present today in the sea spider. The first hemocyanin oligo-hexamer emerged early in euchelicerate evolution, probably associated with the demand for better oxygen supply. Gene duplications led to the formation of a 4 × 6mer hemocyanin in early euchelicerates, which was structurally retained in the arachnids. In xiphosurans, however, an 8 × 6mer hemocyanin built from two identical 4 × 6mers emerged. Although in both arachnids and xiphosurans at least two additional but independent subunit duplications occurred, the architecture of the 4 × 6mer has remained conserved in most taxa for more than 450 Ma. Only in the spiders of the RTA-clade, gene losses and independent duplications gave rise to a novel hemocyanin version, as exemplified by the 2 × 6mer hemocyanin of *C. salei*. Again changing physiological demands may have been the cause for these events. The conservative structure of hemocyanins makes them an excellent marker to trace chelicerate evolution, which is only limited by the absence of hemocyanin in some taxa.

## Abbreviations

CIR: Cox-Ingersoll-Ross process; ESTs: Expressed sequence tags; LOG: Lognormal autocorrelated clock model; Ma: Million years ago; RTA: Retrolateral tibial apophysis; UGM: Uncorrelated gamma multipliers.

## Authors' contributions

TB conceived the study and carried out the phylogenetic analyses. PR, CP and JB provided and analyzed sequence data. PR performed the molecular clock analyses. JM drafted the model of hemocyanin evolution. PR, JM and TB drafted the manuscript. All authors read and approved the final version of the manuscript.

## Supplementary Material

Additional file 1 **List of sequences used in this study**. The accession numbers of the cDNA sequences are given, except (*), which has been derived by conventional protein sequencing. SU = subunit. Click here for file

Additional file 2 **Multiple sequence alignment of chelicerate, crustacean, myriapod and insect hemocyanins, and selected arthropod phenoloxidases**. Click here for file

Additional file 3 **Molecular properties of chelicerate hemocyanin cDNA and the deduced amino acid**. The asterisks (*) denote incomplete N-terminal sequences of *P. imperator *hemocyanins subunits, with 8 and 9 amino acids missing. Click here for file

Additional file 4 **Phylogenetic tree of the chelicerate hemocyanin subunits excluding AgoHcX**. The numbers at the nodes represent Bayesian posterior probabilities estimated with the WAG model of amino acid substitution. The species abbreviations are: Aau, *Androctonus australis*; Ago, *Acanthoscurria gomesiana*; Cro, *Carcinoscorpius rotundicauda*; Csa, *Cupiennius salei*; Eba, *Euphrynichus bacillifer*; Eca, *Eurypelma californicum*; Esp, *Endeis spinosa*; Lpo, *Limulus polyphemus*; Mgi, *Mastigoproctus giganteus*; Nin, *Nephila inaurata*; Pin, *Pandinus imperator*; Ttr, *Tachypleus tridentatus*. See Additional file 1 for abbreviations of the proteins. Click here for file

Additional file 5 **Divergence times of chelicerate hemocyanin subunit types (see Figure 2A)**. Rates across sites were modeled assuming a gamma distribution (Γ) or with a Dirichlet process (D). Divergence time priors were either uniform or modeled with a birth death process. Hard or soft bounds were applied. Divergence times are given in Ma. Click here for file

Additional file 6 **Divergence times of chelicerate taxa, as estimated from the hemocyanin sequences (see Figure 2B)**. Rates across sites were modeled assuming a gamma distribution (Γ) or with a Dirichlet process (D). Divergence time priors were either uniform or modeled with a birth death process. Hard or soft bounds were applied. Divergence times are given in Ma. Click here for file
