# Supplementary material for: The diversity and evolution of chelicerate hemocyanins
Source: BMC Evol Biol. 2012 Feb 14;12:19. doi: 10.1186/1471-2148-12-19 (PMC3306762; doi:10.1186/1471-2148-12-19)
Supplement: Additional file 1 — List of sequences used in this study. The accession numbers of the cDNA sequences are given, except (*), which has been derived by conventional protein sequencing. SU = subunit. [file 1471-2148-12-19-S1.DOC]

**Additional file 1.** List of sequences used in this study. The accession numbers of the cDNA sequences are given, except (*), which has been derived by conventional protein sequencing. SU = subunit.

| **Abbreviation** | **Acc. No.** | **Protein** | **Species** | **Subphylum** | **Order** |
| --- | --- | --- | --- | --- | --- |
| PlaPPO | HE574802 | Prophenoloxidase | *Polyxenus lagurus* | Myriapoda | Diplopoda |
| DmaPPO | FJ381649 | Prophenoloxidase | *Daphnia magna* | Crustacea | Cladocera |
| DmePPOA1 | D45835 | Prophenoloxidase A1 | *Drosophila melanogaster* | Hexapoda | Diptera |
| DmePPO2 | AC006074 | Prophenoloxidase 2 | *Drosophila melanogaster* | Hexapoda | Diptera |
| AfrPPO | AM850109 | Prophenoloxidase | *Artemia franciscana* | Crustacea | Anostraca |
| PseHc | HE574706 | Hemocyanin | *Peripatopsis sedgewicki* | Onychophora |  |
| EpiHc1 | AJ420966 | Hemocyanin | *Epiperipatus sp.* | Onychophora |  |
| EspHc1 | FR865911 | Hemocyanin 1 | *Endeis spinosa* | Chelicerata | Pantopoda |
| LpoHcII | AM260213 | Hemocyanin SU II | *Limulus polyphemus* | Chelicerata | Xiphosura |
| LpoHcIIIa | AM260214 | Hemocyanin SU IIIa | *Limulus polyphemus* | Chelicerata | Xiphosura |
| LpoHcIIIb | FR865912 | Hemocyanin SU IIIb | *Limulus polyphemus* | Chelicerata | Xiphosura |
| LpoHcIV | AM260215 | Hemocyanin SU IV | *Limulus polyphemus* | Chelicerata | Xiphosura |
| LpoHcVI | AM260216 | Hemocyanin SU VI | *Limulus polyphemus* | Chelicerata | Xiphosura |
| CroHcI | DQ090484 | Hemocyanin SU I | *Carcinoscorpius rotundicauda* | Chelicerata | Xiphosura |
| CroHcII | DQ090485 | Hemocyanin SU II | *Carcinoscorpius rotundicauda* | Chelicerata | Xiphosura |
| CroHcIIIa | DQ090486 | Hemocyanin SU IIIa | *Carcinoscorpius rotundicauda* | Chelicerata | Xiphosura |
| CroHcIIIb | DQ090487 | Hemocyanin SU IIIb | *Carcinoscorpius rotundicauda* | Chelicerata | Xiphosura |
| CroHcIV | DQ090488 | Hemocyanin SU IV | *Carcinoscorpius rotundicauda* | Chelicerata | Xiphosura |
| CroHcV | DQ090489 | Hemocyanin SU V | *Carcinoscorpius rotundicauda* | Chelicerata | Xiphosura |
| CroHcVI | DQ090490 | Hemocyanin SU VI | *Carcinoscorpius rotundicauda* | Chelicerata | Xiphosura |
| TtrHcA | none* | Hemocyanin SU α | *Tachypleus tridentatus* | Chelicerata | Xiphosura |
| AauHc6 | P80476* | Hemocyanin SU 6 | *Androctonus australis* | Chelicerata | Scorpiones |
| PimHc2 | FN424080 | Hemocyanin SU 2 | *Pandinus imperator* | Chelicerata | Scorpiones |
| PimHc3A | FN424079 | Hemocyanin SU 3A | *Pandinus imperator* | Chelicerata | Scorpiones |
| PimHc3B | FN424082 | Hemocyanin SU 3B | *Pandinus imperator* | Chelicerata | Scorpiones |
| PimHc3C | FN424081 | Hemocyanin SU 3C | *Pandinus imperator* | Chelicerata | Scorpiones |
| PimHc4 | FN424085 | Hemocyanin SU 4 | *Pandinus imperator* | Chelicerata | Scorpiones |
| PimHc5A | FN424084 | Hemocyanin SU 5A | *Pandinus imperator* | Chelicerata | Scorpiones |
| PimHc5B | FN424086 | Hemocyanin SU 5B | *Pandinus imperator* | Chelicerata | Scorpiones |
| PimHc6 | FN424083 | Hemocyanin SU 6 | *Pandinus imperator* | Chelicerata | Scorpiones |
| EbaHc-a | FR865913 | Hemocyanin SU a | *Euphrynichus bacillifer* | Chelicerata | Amblypygi |
| EbaHc-b | FR865914 | Hemocyanin SU b | *Euphrynichus bacillifer* | Chelicerata | Amblypygi |
| EbaHc-c | FR865915 | Hemocyanin SU c | *Euphrynichus bacillifer* | Chelicerata | Amblypygi |
| EbaHc-d | FR865916 | Hemocyanin SU d | *Euphrynichus bacillifer* | Chelicerata | Amblypygi |
| EbaHc-e | FR865917 | Hemocyanin SU e | *Euphrynichus bacillifer* | Chelicerata | Amblypygi |
| EbaHc-f | FR865918 | Hemocyanin SU f | *Euphrynichus bacillifer* | Chelicerata | Amblypygi |
| EbaHc-g | FR865919 | hemocyanin SU g | *Euphrynichus bacillifer* | Chelicerata | Amblypygi |
| MgiHc-a | FR865920 | hemocyanin SU a | *Mastigoproctus giganteus* | Chelicerata | Uropygi |
| MgiHc-b | FR865921 | hemocyanin SU b | *Mastigoproctus giganteus* | Chelicerata | Uropygi |
| MgiHc-c | FR865922 | hemocyanin SU c | *Mastigoproctus giganteus* | Chelicerata | Uropygi |
| MgiHc-d | FR865923 | hemocyanin SU d | *Mastigoproctus giganteus* | Chelicerata | Uropygi |
| MgiHc-e | FR865924 | hemocyanin SU e | *Mastigoproctus giganteus* | Chelicerata | Uropygi |
| MgiHc-f | FR865925 | hemocyanin SU f | *Mastigoproctus giganteus* | Chelicerata | Uropygi |
| MgiHc-g | FR865926 | hemocyanin SU g | *Mastigoproctus giganteus* | Chelicerata | Uropygi |
| EcaHc-a | X16893 | hemocyanin SU a | *Eurypelma californicum* | Chelicerata | Araneae |
| EcaHc-b | AJ290429 | hemocyanin SU b | *Eurypelma californicum* | Chelicerata | Araneae |
| EcaHc-c | AJ277489 | hemocyanin SU c | *Eurypelma californicum* | Chelicerata | Araneae |
| EcaHc-d | AJ290430 | hemocyanin SU d | *Eurypelma californicum* | Chelicerata | Araneae |
| EcaHc-e | X16894 | hemocyanin SU e | *Eurypelma californicum* | Chelicerata | Araneae |
| EcaHc-f | AJ277491 | hemocyanin SU f | *Eurypelma californicum* | Chelicerata | Araneae |
| EcaHc-g | AJ277492 | hemocyanin SU g | *Eurypelma californicum* | Chelicerata | Araneae |
| AgoHc-a | EST data | hemocyanin SU a | *Acanthoscurria gomesiana* | Chelicerata | Araneae |
| AgoHc-b | EST data | hemocyanin SU b | *Acanthoscurria gomesiana* | Chelicerata | Araneae |
| AgoHc-c | EST data | hemocyanin SU c | *Acanthoscurria gomesiana* | Chelicerata | Araneae |
| AgoHc-d | EST data | hemocyanin SU d | *Acanthoscurria gomesiana* | Chelicerata | Araneae |
| AgoHc-e | EST data | hemocyanin SU e | *Acanthoscurria gomesiana* | Chelicerata | Araneae |
| AgoHc-f | EST data | hemocyanin SU f | *Acanthoscurria gomesiana* | Chelicerata | Araneae |
| AgoHc-g | EST data | hemocyanin SU g | *Acanthoscurria gomesiana* | Chelicerata | Araneae |
| AgoHcX | EST data | unknown hemocyanin | *Acanthoscurria gomesiana* | Chelicerata | Araneae |
| NinHc-a | AJ547807 | hemocyanin SU a | *Nephila inaurata* | Chelicerata | Araneae |
| NinHc-b | AJ547808 | hemocyanin SU b | *Nephila inaurata* | Chelicerata | Araneae |
| NinHc-d | AJ547809 | hemocyanin SU d | *Nephila inaurata* | Chelicerata | Araneae |
| NinHc-e | AJ547810 | hemocyanin SU e | *Nephila inaurata* | Chelicerata | Araneae |
| NinHc-f | AJ547811 | hemocyanin SU f | *Nephila inaurata* | Chelicerata | Araneae |
| NinHc-g | AJ547812 | hemocyanin SU g | *Nephila inaurata* | Chelicerata | Araneae |
| CsaHc1 | AJ307903 | hemocyanin SU 1 | *Cupiennius salei* | Chelicerata | Araneae |
| CsaHc2 | AJ307904 | hemocyanin SU 2 | *Cupiennius salei* | Chelicerata | Araneae |
| CsaHc3 | AJ307905 | hemocyanin SU 3 | *Cupiennius salei* | Chelicerata | Araneae |
| CsaHc4 | AJ307906 | hemocyanin SU 4 | *Cupiennius salei* | Chelicerata | Araneae |
| CsaHc5 | AJ307907 | hemocyanin SU 5 | *Cupiennius salei* | Chelicerata | Araneae |
| CsaHc5' | AJ307908 | hemocyanin SU 5'' | *Cupiennius salei* | Chelicerata | Araneae |
| CsaHc6 | AJ307909 | hemocyanin SU 6 | *Cupiennius salei* | Chelicerata | Araneae |
| CsaHc6' | AJ307910 | hemocyanin SU 6' | *Cupiennius salei* | Chelicerata | Araneae |
| CsaHc6'' | AJ307911 | hemocyanin SU 6'' | *Cupiennius salei* | Chelicerata | Araneae |
| ScoHcA | AJ344359 | hemocyanin SU A | *Scutigera coleoptrata* | Myriapoda | Scutigeromorpha |
| ScoHcD | AJ344360 | hemocyanin SU D | *Scutigera coleoptrata* | Myriapoda | Scutigeromorpha |
| ScoHcC | AJ431379 | hemocyanin SU C | *Scutigera coleoptrata* | Myriapoda | Scutigeromorpha |
| ScoHcB | AJ512793 | hemocyanin SU B | *Scutigera coleoptrata* | Myriapoda | Scutigeromorpha |
| ScoHcX | AJ431378 | hemocyanin SUx | *Scutigera coleoptrata* | Myriapoda | Scutigeromorpha |
| SpiHc1 | AJ297738 | hemocyanin SU A | *Spirostreptus sp.* | Myriapoda | Spirostreptida |
| AgiHc1 | HE574799 | hemocyanin SU A | *Archispirostreptus gigas* | Myriapoda | Spirostreptida |
| PanHc1 | HE574797 | hemocyanin SU 1 | *Polydesmus angustus* | Myriapoda | Polydesmida |
| PanHc2 | HE574798 | hemocyanin SU 2 | *Polydesmus angustus* | Myriapoda | Polydesmida |
| HauHc1 | HE574800 | hemocyanin SU 1 | *Hanseniella audax* | Myriapoda | Symphyla |
| HauHc2 | HE574801 | hemocyanin SU 2 | *Hanseniella audax* | Myriapoda | Symphyla |
| StuHc1 | FM863709 | hemocyanin 1 | *Speleonectes tulumensis* | Crustacea | Remipedia |
| StuHc2 | FM863710 | hemocyanin 2 | *Speleonectes tulumensis* | Crustacea | Remipedia |
| StuHc3 | FM863711 | hemocyanin 3 | *Speleonectes tulumensis* | Crustacea | Remipedia |
| OscHc1 | FM999828 | hemocyanin 1 | *Odontodactylus scyllarus* | Crustacea | Hoplocarida |
| OscHc2 | FM999829 | hemocyanin 2 | *Odontodactylus scyllarus* | Crustacea | Hoplocarida |
| OscHc3 | FM999830 | hemocyanin 3 | *Odontodactylus scyllarus* | Crustacea | Hoplocarida |
| OscHc4 | FM999831 | hemocyanin 4 | *Odontodactylus scyllarus* | Crustacea | Hoplocarida |
| NkeHc | GQ279108 | Hemocyanin | *Nebalia kensleyi* | Crustacea | Phyllocarida |
| GpuHc1 | EST data | Hemocyanin | *Gammarus pulex* | Crustacea | Peracarida |
| GroHc1 | AJ937836 | Hemocyanin | *Gammarus roeseli* | Crustacea | Peracarida |
| CcsHc | DQ230983 | Hemocyanin | *Cyamus scammoni* | Crustacea | Peracarida |
| EpuHc1 | GQ153951 | hemocyanin SU 1 | *Eurydice pulchra* | Crustacea | Peracarida |
| EpuHc2 | GQ153952 | hemocyanin SU 2 | *Eurydice pulchra* | Crustacea | Peracarida |
| HamHcA | AJ272095 | hemocyanin SU A | *Homarus americanus* | Crustacea | Decapoda |
| PleHc | AF522504 | hemocyanin SU 1 | *Pacifastacus leniusculus* | Crustacea | Decapoda |
| PleHc2 | AY193781 | hemocyanin SU 2 | *Pacifastacus leniusculus* | Crustacea | Decapoda |
| PciHc1 | EST data | hemocyanin SU 1 | *Petrolisthes cinctipes* | Crustacea | Decapoda |
| PciHc2 | EST data | hemocyanin SU 2 | *Petrolisthes cinctipes* | Crustacea | Decapoda |
| PinHcA | P04254* | hemocyanin SU a | *Panulirus interruptus* | Crustacea | Decapoda |
| PinHcB | P10787* | hemocyanin SU b | *Panulirus interruptus* | Crustacea | Decapoda |
| PinHcC | S21221* | hemocyanin C | *Panulirus interruptus* | Crustacea | Decapoda |
| PvuHc | P80888* | hemocyanin | *Palinurus vulgaris* | Crustacea | Decapoda |
| PelHc1 | AJ344361 | hemocyanin SU 1 | *Palinurus elephas* | Crustacea | Decapoda |
| PelHc2 | AJ344362 | hemocyanin SU 2 | *Palinurus elephas* | Crustacea | Decapoda |
| PelHc3 | AJ344363 | hemocyanin SU 3 | *Palinurus elephas* | Crustacea | Decapoda |
| PelHc4 | AJ516004 | hemocyanin SU 4 | *Palinurus elephas* | Crustacea | Decapoda |
| CmaHc1 | AY861676 | hemocyanin SU 1 | *Cancer magister* | Crustacea | Decapoda |
| CmaHc2 | AY861677 | hemocyanin SU 2 | *Cancer magister* | Crustacea | Decapoda |
| CmaHc3 | AY861678 | hemocyanin SU 3 | *Cancer magister* | Crustacea | Decapoda |
| CmaHc4 | AY861679 | hemocyanin SU 4 | *Cancer magister* | Crustacea | Decapoda |
| CmaHc5 | AY861680 | hemocyanin SU 5 | *Cancer magister* | Crustacea | Decapoda |
| CmaHc6 | U48881 | hemocyanin SU 6 | *Cancer magister* | Crustacea | Decapoda |
| CsaHc | AF249297 | hemocyanin | *Callinectes sapidus* | Crustacea | Decapoda |
| PvaHc1 | AJ250830 | hemocyanin SU 1 | *Penaeus vannamei* | Crustacea | Decapoda |
| PvaHc | X82502 | hemocyanin | *Penaeus vannamei* | Crustacea | Decapoda |
| MjaHcL | EF375711 | hemocyanin L | *Marsupenaeus japonicus* | Crustacea | Decapoda |
| MjaHcY | EF375712 | hemocyanin Y | *Marsupenaeus japonicus* | Crustacea | Decapoda |
| FchHc | FJ594414 | hemocyanin | *Fenneropenaeus chinensis* | Crustacea | Decapoda |
| CjaHc1 | HE574707 | hemocyanin SU 1 | *Caridina japonica* | Crustacea | Decapoda |
| ScuHc1 | FM242638 | hemocyanin SU 1 | *Sinella curviseta* | Hexapoda | Collembola |
| TdoHc1 | FM165288 | hemocyanin SU 1 | *Thermobia domestica* | Hexapoda | Thysanura |
| TdoHc2 | FM165289 | hemocyanin SU 2 | *Thermobia domestica* | Hexapoda | Thysanura |
| MgeHc1 | FM242639 | hemocyanin SU 1 | *Machilis germanica* | Hexapoda | Archaeognatha |
| PgrHc1 | DQ118369 | hemocyanin SU 1 | *Perla grandis* | Hexapoda | Plecoptera |
| PgrHc2 | DQ118370 | hemocyanin SU 2 | *Perla grandis* | Hexapoda | Plecoptera |
| PmaHc1 | AJ555403 | hemocyanin SU 1 | *Perla marginata* | Hexapoda | Plecoptera |
| PmaHc2 | AJ555404 | hemocyanin SU 2 | *Perla marginata* | Hexapoda | Plecoptera |
| SamHc | AF038569 | Hemocyanin | *Schistocerca americana* | Hexapoda | Orthoptera |
| BduHc1 | FM242646 | hemocyanin SU 1 | *Blaptica dubia* | Hexapoda | Blattaria |
| BduHc2 | FM242647 | hemocyanin SU 2 | *Blaptica dubia* | Hexapoda | Blattaria |
| PamHc1 | FM242648 | hemocyanin SU 1 | *Periplaneta americana* | Hexapoda | Blattaria |
| PamHc2 | FM242649 | hemocyanin SU 2 | *Periplaneta americana* | Hexapoda | Blattaria |
| CseHc1 | FM242644 | hemocyanin SU 1 | *Cryptotermes secundus* | Hexapoda | Isoptera |
| CseHc2 | FM242645 | hemocyanin SU 2 | *Cryptotermes secundus* | Hexapoda | Isoptera |
| HmeHc1 | FM242642 | hemocyanin SU 1 | *Hierodula membranacea* | Hexapoda | Mantodea |
| HmeHc2 | FM242643 | hemocyanin SU 2 | *Hierodula membranacea* | Hexapoda | Mantodea |
| CmoHc1 | FM242640 | hemocyanin SU 1 | *Carausius morosus* | Hexapoda | Phasmatodea |
| CacHc1 | FM242641 | hemocyanin SU 1 | *Chelidurella acanthopygia* | Hexapoda | Dermaptera |
