# Supplementary material for: The diversity and evolution of chelicerate hemocyanins
Source: BMC Evol Biol. 2012 Feb 14;12:19. doi: 10.1186/1471-2148-12-19 (PMC3306762; doi:10.1186/1471-2148-12-19)
Supplement: Additional file 2 — Multiple sequence alignment of chelicerate, crustacean, myriapod and insect hemocyanins, and selected arthropod phenoloxidases. [file 1471-2148-12-19-S2.DOC]

**Additional file 2.** Multiple sequence alignment of chelicerate, crustacean, myriapod and insect hemocyanins, and selected arthropod phenoloxidases.

#NEXUS

Begin data;

Dimensions ntax=143 nchar=912;

Format datatype=protein gap=- missing=X interleave;

Matrix

PlaPPO SKITQD-QYKAL-----ITLLYQFSRPL------DKQKQH---PYPEVDL

AfrPPO MAGRNINSQEKL-----FLLLETPSEPVFKPKKYKNEEVYIDVPDKYLQG

DmaPPO MSDLEL-QQRQV-----FNLFERVSQPFSPKPNITFEFPNFVSP-TDLSS

DmePPOA1 NT-----DLKAL-----ELMFQRPLE-----------------P------

DmePPO2 MA-----DKKNL-----LLLFDHPTEPV----------------------

PseHc SDAVSTTSEGNVCSMSYLSALEPLENPTEDDGCSIASFTLEDGTIGPVD-

EpiHc1 MPSTS--QQKEV-----LDFFNIPETRRVSRAKQKDKPSVIFKPL-----

EspHc1 MA-LLD-KQKKI-----IDLFYHLSTPP----------------------

PimHc2 XX-XXX-XXXKI-----LPLFKHLTS------------------------

PimHc3A MT-LHQ-KQVQV-----LKLFEFLNEV-----------------------

PimHc3B MV-LHE-KQIRI-----LKLFKQLSVA-----------------------

PimHc3C MS-TKD-LEKRI-----LPLFEFAPL------------------------

PimHc4 XX-XXX-XXXXV-----LKLFEHLTS------------------------

PimHc5A MT-VQD-KQKQL-----LPLFKHLTS------------------------

PimHc5B AD-VVE-KQKRV-----TPLFQFVVL------------------------

PimHc6 MP-AQE-KQAKL-----LPLFEHLTA------------------------

AauHc6 -T-VAD-KQARL-----MPLFKHLTA------------------------

LpoHcII MT-LHD-KQIRV-----CHLFEQLSS------------------------

LpoHcIIIa MT-VKE-KQSRL-----LPLFKHLTS------------------------

LpoHcIIIb MT-IQE-KQNHI-----LSLLEHLNN------------------------

LpoHcIV MT-LKE-KQDRI-----LVLFEHLTS------------------------

LpoHcVI LG-ALE-KQLRV-----LPLFEYASI------------------------

CroHcVI LD-TVE-KQLRV-----LPLFEYASI------------------------

CroHcV LS-VLQ-KQLRV-----LPLFECATV------------------------

CroHcIV MT-LKE-KQDRI-----LALFEHLTS------------------------

CroHcIIIb MT-IQE-KQNKI-----LSLLEHLNN------------------------

CroHcIIIa MT-VKE-KQSRL-----LPLFKHLTS------------------------

CroHcII MT-LHD-NQVRV-----CHLFEQLSS------------------------

CroHcI MT-IKE-KQASI-----LALFEHLTS------------------------

AgoHcA MTILHD-KQVQA-----LKLFEKLSVA-----------------------

AgoHcB MPSTAE-KQRRI-----LPFFQFTSL------------------------

AgoHcC SD-ANE-MQARL-----LQLFEHSTL------------------------

AgoHcD MT-IAD-HQARI-----LPLFKKLTS------------------------

AgoHcE MP-DKQ-KQLRV-----ISLFEHMTS------------------------

AgoHcF MT-VQD-KQRQI-----LPLFEHLTS------------------------

AgoHcG MASIPE-KQALI-----LPLFEKLTS------------------------

AgoHcX VP-LRQ-TQDLL-----L-IFSHVGYPV----------------------

EcaHcA MTILHD-KQVQA-----LKLFEKLSVA-----------------------

NinHcA MSLLHE-KQVRV-----LKLFERLSVA-----------------------

EcaHcB MPSTAE-KQRRI-----LPFFQFTSL------------------------

NinHcB MSSASE-KQKRV-----LPLFQYVSF------------------------

EcaHcC SD-ANE-MQARL-----LQLFEHSTL------------------------

EcaHcD MT-IAD-HQARI-----LPLFKKLTS------------------------

NinHcD MS-VKD-KQAQI-----LPLFKNLTA------------------------

EcaHcE MP-DKQ-KQLRV-----ISLFEHMTS------------------------

NinHcE MS-VKE-KQQRV-----CSLFTHLTS------------------------

EcaHcF MT-VQD-KQRQI-----LPLFEHLTS------------------------

NinHcF MP-AQD-KQRRI-----LPLFEHLAS------------------------

EcaHcG MASIPE-KQALI-----LPLFEKLTS------------------------

NinHcG MS-IKE-KQDQI-----LPLFEKLTT------------------------

TtrHcA -T-IKE-KQASI-----LALFEHLTS------------------------

EbaHcA MS-LHG-KQVVV-----LKLFDRLSLA-----------------------

MgiHcA MT-IHD-RQIAV-----LKLYKYLSVAT----------------------

EbaHcB SS-AAE-KQRRI-----LPLFQKIEY------------------------

MgiHcB MP-VKD-KQDRI-----LPLFEYVAL------------------------

EbaHcC SE-TGE-KQKRV-----VGLFEYVAL------------------------

MgiHcC SS-VAD-KQKRI-----IPLFTYATS------------------------

EbaHcD MA-LKD-KQLRI-----LALFKKLTS------------------------

MgiHcD MT-VKD-KQKEI-----LTLFKGLTS------------------------

EbaHcE MT-VKE-KQARL-----LPLFEKLTA------------------------

MgiHcE MT-VKE-QQKRI-----LDLFHHLTS------------------------

EbaHcF MT-VKE-RQARI-----LPLFEHLAS------------------------

MgiHcF MA-SQD-HQKRI-----LHFFKHLAY------------------------

EbaHcG MT-VKE-RQDLL-----LPLFEHMTT------------------------

MgiHcG MS-QQE-KQLQV-----LALLEGLTS------------------------

CsaHc1 MA-IKD-KVEKL-----LPLFEKLTS------------------------

CsaHc2 MT-IKE-KADRI-----LALFEQLTS------------------------

CsaHc3 MT-VKE-MYDRI-----LPLFEKLTS------------------------

CsaHc4 MT-IKE-KEDRI-----LPLFENLTS------------------------

CsaHc5a MT-VKE-KADRI-----LPLFEQLTT------------------------

CsaHc5b MT-VKE-KADRI-----LPLFEQLTT------------------------

CsaHc6a MT-IKE-KEDRI-----LPLFENLTS------------------------

CsaHc6b MT-IKE-KEDRI-----LPLFENLTS------------------------

CsaHc6c MT-IKE-KEDRI-----LPLFENLTS------------------------

HauHcB -TDVEA-KQAKV-----KEVIQYINRPHIHSGGSCDAEASHLTA------

HauHcA -TDVEA-KQAKV-----KEVIMHINRPHVHAGGSCEPPPTKLSA------

ScoHcA PADTKD-KLEKI-----LELIGHVNRPLTPETPEPAGYDE----------

ScoHcD VNDYKL-KQKEI-----QHLVDTINKPVYPDFKD---TRGIIDE------

ScoHcX GTAEEK-HQKEI-----YDLVQRINRPLIPQFKEPNFPTSFLIKGKDPK-

ScoHcC DEDSQV-KERQI-----LHIVDSINKPISPDFRA---PRGVIDE------

ScoHcB -TDQEA-KQKRM-----LEVLQHVNKPYVAETKDPKIPAGSE--------

SpiHc1 -VNVEA-KQKMV-----DTLISYINKPANPVPPKPPIDP-----------

AgiHc1 -VNVEA-KQKMV-----DTLISYINKPANPVPPKPPIDP-----------

PanHc1 -TNVEA-KQAMV-----DNLLTFINKPDNPPAPVPNVDP-----------

PanHc2 -TNTKL-KQLQI-----TQIISKINKPVPAEIRS---SENIIEK------

PciHc1 SRSRAE-RQLGL-----NRLVNKITSPLSSSYAELKEAATNYDPTAHDDH

PciHc2 ADEDAH-KQQVI-----NRLLNRITSPIPSAYAELKNAAETYDPGHDDSH

NkeHc AGTPVD-RQQAV-----LQLLFKPTEPIRDRFTDLKSAAASFDPTADKSQ

GpuHc1 APSLAV-RQQAV-----NHLLYRVTEPLRF--DDLKAAAASFNPVADKSI

GroHc1 APSLAV-RQQAV-----NHLLYRVTEPLRF--DDLKAAAASFNPVADKSI

CcsHc TDSLTQ-RQHDI-----NQILTRITEDLT---GDAKALAESFDPIADTSI

EpuHc1 APPVAK-RQQDV-----LRLLFKVTEPIRARFEDLKNSAKSFNPIADLTH

EpuHc2 PVSSAK-KQQDI-----LRLLYKVTEHIRERFTDLKDASKNFDPLADLTH

OscHc1 ATDLAH-KQQTI-----NQLLYKSTEPIRSKFSELKKISETWQPLDHKHH

OscHc3 AEDLAH-KQQAI-----NQLLYKSTEPIRSKFAELKEISKSWKPLDHKHH

OscHc2 XXXXXX-XXXXX-----XXXXXXXTEPIRSKFTDLKEASKSFTPTG----

OscHc4 -TDVAH-KQQAI-----NRLLYKVTEPIRSKFTDLKEASKSFTPTG----

PleHc DTDVAH-QQQAI-----NRLLYKVTSHIKSSFTDLKEAAETWNPRDHTDK

PleHc2 DATLAK-RQQVV-----NHLLDYIYDHLHY--TDLKGIAGTFSPEADTSI

HamHcA GDSTAK-RQQDV-----NHLLDRIYDHLHY--SDLKQISETFSPEADTSM

PinHcB GTGNAN-KQQDI-----NHLLDKIYEPTKY--PDLKDIAENFNPLGDTSI

PinHcA GTGNAQ-KQQDI-----NHLLDKIYEPTKY--PDLKEIAENFNPLGDTSI

PvuHc SSDNAH-KQHDV-----NHLLDKIYEPIKD--EKLHNTAHTFNPVADTSI

PelHc1 SSDNAH-KQQDV-----NHLLDKIYEPIKD--EELHNIAQTFDPVADTSI

PelHc2 SSDNAH-KQQDV-----NHLLDKIYEPIKD--EELHNIAQTFDPVADTSI

PelHc3 SSDNAH-KQQDV-----NHLLDKIYEPIKD--EKLHNTAQTFDPVADTSI

PelHc4 SSDNAH-KQQDV-----NHLLDKIYEPIKD--EELHNIAQTFDPVADTSI

CmaHc6 ----AQ-KQHDV-----NSALWKVYEDIQD--PHLIQLSQNFDPLS--GH

CmaHc1 GADLAH-RQQAV-----NRLLYKIYSPIPSAFAKLKEHATTFNPRDHISH

CmaHc2 GADLAH-RQQAV-----NRLLYKIYSPIPSAFAKLKEHATTFNPRDHISH

CmaHc3 HVSLAH-KQHDV-----NSALWKVYEDIRD--THLKELSQSFDPLS--GQ

CmaHc4 ----AH-KQHDV-----NSVLWKVYEDIRD--PHLKELSQSFDPLS--GH

CmaHc5 ----VQ-KQHDV-----NSALWKVYEDIRD--SNLIQLSQSFDPLS--GH

CsaHc ----AQ-KQHDV-----NSILWKVYEEIRD--PHLKELSQSFDPLS--GH

PinHcC DKLLAQ-KQHDV-----NYLVYKLYGDIRD--DHLKELGETFNPQGDLLL

PvaHc1 GESDAQ-KQHDV-----NFLLHKIYGNIRY--SDLKAKADSFDPAGRFGS

PvaHc ASADVQ-QQKDV-----LYLLNKIYGDIQD--GDLLATANSFDPVGNLGS

FchHc1 DVSDAQ-KQHDI-----NFLLHKIYGEIRD--PNLKGKADSFDPEADLSH

MjaHcL GVSDAQ-KQHDI-----NFLLHKIYGDIRD--DALKAKADSFDPEADLSH

MjaHcY GADDVQ-KQKDI-----LYLVHKIYGDIQD--ADLKATANSFDPVADLGI

CjaHc1 DASNAQ-KQHDV-----NFLLWKVNEDLRD--DKLKGLSTSFNPEGDLSH

PmaHc2 DDTFYK-HQTQV-----LHLVEHIYEPISSHYEDLTTIKKTYDPLQHLDN

StuHc2 DEVFLK-RQRDI-----LLLLLRLHQPNAI--PEQKSISESYDPLMHLSH

PgrHc2 DDTFYK-HQTQV-----LHLVEHIYEPISSHYEDLIYIKKTYDPLQHLDL

CseHc2 DAIFLK-RQLQV-----LNLLRHLNQPLID--PELLVFKNSFSPLNNLNK

PamHc2 DMTFLT-RQLQV-----LKLLRHLNQPLSD--PELTAIKNSFSPLNHLNK

BduHc2 DGVFLN-RQISV-----LKLLQHLNQPLVD--SDLTAYKESFSPLNNLNR

HmeHc2 DATFLK-RQLHV-----LKLLRHISEPITD--PELHAKKQSGDPANHLNK

TdoHc2 DETFLK-RQMLI-----LKLLRHISEPLED--PELVQIKDSYSPLNSLSV

StuHc3 DKGFLK-RQKDV-----LTLLNHVFSHFQD--AAHQEDGRTYEPLNHLTS

StuHc1 DADFLQ-KQSKI-----LKLFNKIHEHNRY--NDQVDIATSFNPLDHLGD

PmaHc1 DQDFLT-RQRDV-----IRLCMKVHEHNHY--QEQVDLVKDYDPSVA-GK

SamHc1 DKPFLM-KQREV-----LRLFNKVHEPNRY--KEQVELGNAYEPLNSLPR

BduHc1 DREFLT-RQNEI-----LHLLNKVHEPNRF--KDQSELGRSYDPTAHFDK

PamHc1 DKEFLV-RQREV-----LRLLNKVHEPNRF--KEQAELGRSYDPTAHFSK

CseHc1 DKIFLS-KQREV-----LKLLNKVHEPNRF--KDQADLGRSYDPTQHLSK

HmeHc1 DKDFLV-KQRQI-----LRLCNKVHEPNRF--KEQAELGKNYEPLKHLDK

CmoHc1 DKDFLL-KQREV-----LKLLNKPHEPNRY--KDQADSGSKYEPSNNLNR

CacHc1 DSGLVV-RQYEI-----LKLLNKVHEPNRI--KEQIEIGNSFDLSTALTG

TdoHc1 DKEFLL-KQKEI-----LQLLNKVHEPNRF--KEQVNLGNSYDPLANLGN

PgrHc1 DHEFLV-RQRDI-----IRLCMKVHQHNHY--EEQVDLVKDYDPSVA-GK

ScuHc1 DQEFLT-KQKEI-----VKLLNKVHELNFY--QDQATIGKDWDPLAHLDS

MgeHc1 DHEFLV-KQREI-----LKLLFRAHEPNIS--EDQVDIAAKFDIEANLDK

PlaPPO SFVPPENELEEYRQRID---------------------------------

AfrPPO GFKDLKPILTGGIGLRDGRPGKVPSPLSVRGVEL----------------

DmaPPO KPGLATPASAPTDGLNI---------------------------------

DmePPOA1 -----------AFTTRDSGK--TVLELPDSFYTDRYRNDTEEVGNRFSKD

DmePPO2 --------------FMDKGKRVTVFDVPDSFLTDRYRPISNEVQSRVGDK

PseHc --------------------------------------------------

EpiHc1 --------------------------------------------------

EspHc1 ----MTEGAGEMGEHMD---------------------------------

PimHc2 ----LTRTQLS-EGLRD---------------------------------

PimHc3A ----AYHRDAP-H---D---------------------------------

PimHc3B ----TVGGGVP-KERRD---------------------------------

PimHc3C ----ETKEKFSLFAERT---------------------------------

PimHc4 ----LVEEQIK-PEDRD---------------------------------

PimHc5A ----LTTEQLP-ADERD---------------------------------

PimHc5B ----STRQKFSLRAEKD---------------------------------

PimHc6 ----LTREKLP-EDQRD---------------------------------

AauHc6 ----LTREKLP-LDQRD---------------------------------

LpoHcII ----ATVIGNGDKHKHS---------------------------------

LpoHcIIIa ----LTRDQLP-VGLRD---------------------------------

LpoHcIIIb ----LTKHQLP-VDQRD---------------------------------

LpoHcIV ----LTKHQLP-EDQRD---------------------------------

LpoHcVI ----PTKEKFALKAHRD---------------------------------

CroHcVI ----STKEKFALKVHRD---------------------------------

CroHcV ----PTKEKFALKAYRD---------------------------------

CroHcIV ----LTKHQLP-EEQRD---------------------------------

CroHcIIIb ----LTKHQLP-VDERD---------------------------------

CroHcIIIa ----LTRDQPP-VSQRD---------------------------------

CroHcII ----ATVTGNGEKHKHS---------------------------------

CroHcI ----VPKQHIP-EKERD---------------------------------

AgoHcA ----ATGDAIP-ADQID---------------------------------

AgoHcB ----STKDKFGILVQRD---------------------------------

AgoHcC ----STKAKFGLRVIRD---------------------------------

AgoHcD ----LSPDPLP-EAERD---------------------------------

AgoHcE ----IN-TPLP-PDQID---------------------------------

AgoHcF ----LTRGGLN-RTESD---------------------------------

AgoHcG ----LTKETPP-RAQWD---------------------------------

AgoHcX ----RLVDERR-LDRDN---------------------------------

EcaHcA ----ATGEPVP-ADQID---------------------------------

NinHcA ----ASGEKIP-ADQID---------------------------------

EcaHcB ----STKDKFGILVQRD---------------------------------

NinHcB ----STKDKFGMRVQRD---------------------------------

EcaHcC ----STKAKFGLRVIRD---------------------------------

EcaHcD ----LSPDPLP-EAERD---------------------------------

NinHcD ----LAPETLP-EAERD---------------------------------

EcaHcE ----IN-TPLP-RDQID---------------------------------

NinHcE ----ISRTAVP-LADRD---------------------------------

EcaHcF ----LTRGGLD-RTESD---------------------------------

NinHcF ----LTRAVLP-PEERD---------------------------------

EcaHcG ----LTKETPP-RAQWD---------------------------------

NinHcG ----LTRQQIP-PEQRD---------------------------------

TtrHcA ----VPKQHIP-EKERD---------------------------------

EbaHcA ----AVGDKLP-KEERD---------------------------------

MgiHcA ----VTGGVHE-DDDVD---------------------------------

EbaHcB ----NTSNKFSILVRDD---------------------------------

MgiHcB ----DTSDKFGLRVQRD---------------------------------

EbaHcC ----TTRSKFGLRVQRD---------------------------------

MgiHcC ----KTKDKFGMRVQRE---------------------------------

EbaHcD ----VTGDPLP-EGDRD---------------------------------

MgiHcD ----LSHTPLP-EAERD---------------------------------

EbaHcE ----LTHERIP-EDQLD---------------------------------

MgiHcE ----LPKERTP---PTD---------------------------------

EbaHcF ----QTRAQLP-VDERD---------------------------------

MgiHcF ----VTHVALP-EDERD---------------------------------

EbaHcG ----LSRESIP-PDQRD---------------------------------

MgiHcG ----LSHESLP-VDQRG---------------------------------

CsaHc1 ----LTKQQLA-PDQRD---------------------------------

CsaHc2 ----LTKRQLP-VDQRD---------------------------------

CsaHc3 ----LTRLQLP-VEERD---------------------------------

CsaHc4 ----LTRHQLP-VDQRD---------------------------------

CsaHc5a ----LTRHQLP-PEERD---------------------------------

CsaHc5b ----LTRHQLP-PEERD---------------------------------

CsaHc6a ----LTRQQLP-EDQRD---------------------------------

CsaHc6b ----LTRQQLP-EDQRD---------------------------------

CsaHc6c ----LTRQQLP-EDQRD---------------------------------

HauHcB --------------------------------------------------

HauHcA --------------------------------------------------

ScoHcA --------------------------------------------------

ScoHcD --------------------------------------------------

ScoHcX --------------------------------------------------

ScoHcC --------------------------------------------------

ScoHcB --------------------------------------------------

SpiHc1 --------------------------------------------------

AgiHc1 --------------------------------------------------

PanHc1 --------------------------------------------------

PanHc2 --------------------------------------------------

PciHc1 FSDGGAAAHNLVEEIQD---------------------------------

PciHc2 YTDGGEAARHLVQEIND---------------------------------

NkeHc YSDGGAAAAHLMEEIND---------------------------------

GpuHc1 YTDGGVAAQHLVDEIAD---------------------------------

GroHc1 YTDGGAAAQHLVDELAD---------------------------------

CcsHc YKDDGKAAKKLVQEIKD---------------------------------

EpuHc1 YTDGGEAVKRLQQEVDD---------------------------------

EpuHc2 YTDGGASVKTLMGEIDD---------------------------------

OscHc1 CSDGGKALERLTREISD---------------------------------

OscHc3 CTDGGHALERLVKEVED---------------------------------

OscHc2 ------AGAKLDKEHKD---------------------------------

OscHc4 ------AGAKLDKEHKD---------------------------------

PleHc SSDGGAAIKHLMDELDD---------------------------------

PleHc2 YTDDGAAAHVLMEELRD---------------------------------

HamHcA YTDGGTAAHHLMEELND---------------------------------

PinHcB YNDHGAAAEALMKELND---------------------------------

PinHcA YNDHGAAVETLMKELND---------------------------------

PvuHc YGDDGAAAKTLMQKLND---------------------------------

PelHc1 YGDVGAAAKTLMQELND---------------------------------

PelHc2 YVDDGAAAKTLMQELND---------------------------------

PelHc3 YGDDGAAAKTLMQELND---------------------------------

PelHc4 YVDDGAAAKTLMQELND---------------------------------

CmaHc6 YDDDGVAAKRLMKELNE---------------------------------

CmaHc1 CNDGGNSVNHLMDEIEA---------------------------------

CmaHc2 CNDGGNSVNHLMDEIEA---------------------------------

CmaHc3 YDDNGVAAKRLMKELNE---------------------------------

CmaHc4 YDDDGVAAKRLMKELNE---------------------------------

CmaHc5 YDDDGVAAKRLMKELNE---------------------------------

CsaHc YDDDGVAAKRLMKELND---------------------------------

PinHcC YHDNGASVNTLMADFKD---------------------------------

PvaHc1 YSDGGEAVQKLVREVKD---------------------------------

PvaHc YSDGGAAVQKLVQDLND---------------------------------

FchHc1 YSDSGEAVHKLIRDLKD---------------------------------

MjaHcL YSDDGEAVHTLIRDLKD---------------------------------

MjaHcY YSDGGAAAQRLVKDLND---------------------------------

CjaHc1 YDDNGAAVHRLVQEYKD---------------------------------

PmaHc2 FKD-GDHVKHFVHDVTH---------------------------------

StuHc2 FKK-PELVQELVDEITH---------------------------------

PgrHc2 FKD-GEQVKHFVHDVTH---------------------------------

CseHc2 FKD-PTAVKNFLKLVTE---------------------------------

PamHc2 FKE-PETVKLLVGLITK---------------------------------

BduHc2 YRD-AEVVKQYVGLVES---------------------------------

HmeHc2 FND-PKAVRHFVNLVRK---------------------------------

TdoHc2 YKD-PQLVQKVVRKLEN---------------------------------

StuHc3 YQD-PTPVKKLVRMYKM---------------------------------

StuHc1 FKH-RDCVLKLVKKYKA---------------------------------

PmaHc1 FKD-VTPIKRLMKYYNA---------------------------------

SamHc1 YRN-PAPVKQLVRLYRA---------------------------------

BduHc1 YKN-ASPVKSLVKLYTS---------------------------------

PamHc1 YKN-AVPVKRMVKEYMA---------------------------------

CseHc1 YKN-ALPVKTLVKQYTS---------------------------------

HmeHc1 YKH-PRSVKTLVKYYNS---------------------------------

CmoHc1 YKN-PVPVKTLVKRYNS---------------------------------

CacHc1 FKN-PNAAKALLKGYKA---------------------------------

TdoHc1 YKK-AAAVHQLVKLYNN---------------------------------

PgrHc1 FKD-VTPIKKLMKYYNA---------------------------------

ScuHc1 YKN-VRVVKELVKELKN---------------------------------

MgeHc1 FKE-VDSVKELYELYVK---------------------------------

PlaPPO -------------LEGHHVGQ-LPRRQPCELFDPQVMNEATTLAQIFTEQ

AfrPPO -------------PDLSPVLT-VPRGRPWSVFYDVDKKAGNALVKMMNTF

DmaPPO -------------PELDLVKT-IPRGRLFSNFHPGHRVAAYTLVKIFKDA

DmePPOA1 VDLKIPIQELSNVPSLEFTKK-IGLKNQFSLFNNRHREIASELITLFMSA

DmePPO2 VEQRVPVREISI-PDLRIPMS-LGRDEQFSLFLPKHRRIAGRLIDIFMNM

PseHc ---------------VPGIGN-LKKDDLFSTNVREHRKEAQLLFNLFLDC

EpiHc1 ---------------PAGIGK-LKKGNLFSPFIKRHREESTKVIEFLLDS

EspHc1 -------------ARLHNLGS-TPRRIVFSVFHEKHLKEATELFEILIAA

PimHc2 -------------PRLKSVGK-LDRGSLFSCFHADHLHEAQTLAETLYAA

PimHc3A -------------ERLKHVGT-LSPKSIFSCFHPVHLAEARRLVEVYLAA

PimHc3B -------------PRLASHVIVVEPGNIFSCFHPDHLEEARQLYEVFYEA

PimHc3C -------------GK--HLGI-LGRGQLFSCFHRDHLQEATEFYEMLIGA

PimHc4 -------------PRLHGVGI-LPRGTLFSCFHAKHLDEATKLYETLYAA

PimHc5A -------------PRLKDVGV-LKRGKLFSCFHEEHLLEAERLFTVLYQA

PimHc5B -------------ERFRGLGI-LGRGKLFSCFHRDHLEEARNLYELLIEA

PimHc6 -------------ERLKGIGI-LPRGTLFSCFHAKHLAEATKLYETLYAA

AauHc6 -------------ERLKGVGI-LPRGTLFSCFHARHLAEATELYVALYGA

LpoHcII -------------DRLKNVGK-LQPGAIFSCFHPDHLEEARHLYEVFWEA

LpoHcIIIa -------------DRLKDVGH-LPRGTLFSLFHAKHLEEATHLYEILYGA

LpoHcIIIb -------------SRLKDVGH-LHRGELFSLFHMEHLIEATHLYETLFKA

LpoHcIV -------------PRLHDLGH-LHRGELFSCFHKEHLEEATHLYETLYKA

LpoHcVI -------------PKLQGLGV-LGRGQLFSLFHAEHLAAATKLYEVLIGA

CroHcVI -------------PKLQGLGV-LGRGQLFSLFHAEHLAEATKLYEVLIGA

CroHcV -------------HKFEGVGI-LGRGQLFSLFHAEHLAEATKLYEVLIAA

CroHcIV -------------ARLHDLGH-LLRGELSSCFHKEHLEEATHLYEALYKA

CroHcIIIb -------------PRLKDVGH-LLRGGLFSLFHMEHLTEATHLYETLYNA

CroHcIIIa -------------DRLKDVGH-LPRGTLFSLFHSKHLEEATHLYEILYGA

CroHcII -------------DRLKNVSK-LQPGAIFSCFHPEHLEEARHLYEVFWEA

CroHcI -------------NRLHDVGH-LSRGKLFSLFHREHLEEATHLYEILHAA

AgoHcA -------------ERLRNITT-LGPHEFFSCFYPDHLEQAKRVYEVFCRA

AgoHcB -------------PRLAGLGV-LGRGVLFSCFHEAHLKEATQLYEVLIQC

AgoHcC -------------PKLAGIGI-LGRGKIFSCFHEDHLQEASHLAEVLVGA

AgoHcD -------------PRLKGVGF-LPRGTLFSCFHEEHLAEAETLAERLVEA

AgoHcE -------------PRLHHLGH-LHQGELFSCFHEDDLAEATELYKILYTA

AgoHcF -------------VRLRRVGR-LPRGTLFSCFHSEHLKEATELYQILYKA

AgoHcG -------------VRLSGVGV-LPRGTLFSCFHEKHLLEATKLFKILYSA

AgoHcX -------------PRLRRLGR-LSRREIFSTFNAEHIAEAKELYTILLGA

EcaHcA -------------ERLRNITT-LGPNEFFSCFYPDHLEQAKRVYEVFCHA

NinHcA -------------ARLSTVGE-LPNSAFFSCFLPAHLEEAKRLIEIFYSA

EcaHcB -------------PRLAGLGV-LGRGVLFSCFHEEHLKEATQLYEVLIEC

NinHcB -------------PRLSGLGV-LGRGVLFSCFHEDHLKEATQLYEVLITA

EcaHcC -------------PKLAGIGI-LGRGKIFSCFHEDHLEEASRLAEVLVGA

EcaHcD -------------PRLKGVGF-LPRGTLFSCFHEEHLAEAETLAEALVEA

NinHcD -------------PRLKGVGF-LPRGRLFSCFHEDHLGEAQALYEVLYEA

EcaHcE -------------ARLHHLGR-LPQGELFSCFHEEDLEEATELYKILYTA

NinHcE -------------PRLHGIGK-LAQGELFSCFHEKGLAEATKLYETLYAA

EcaHcF -------------VRLRRVGR-LPRGTLFSCFHSEHLKEATELYQILYKA

NinHcF -------------PRLKRLGR-LPRGTLFSCFHTEHLIEAEELFETLYAA

EcaHcG -------------PRLAGVGV-LPRGTLFSCFHEKHLLEATKLFKVLYSA

NinHcG -------------PRLIGVGV-LPRGTLFSCFHERHLKEATKLFEVLYKA

TtrHcA -------------NRLHDVGH-LSRGKLFSLFHREHLEEATHLYEILHAA

EbaHcA -------------PRLKNVGI-LKPTEIFSCFKPEHLEEARHLYETFYSA

MgiHcA -------------ARLHGIGKGVSTSEIFSCFYPKHLEAAEKLYKVLYEA

EbaHcB -------------PRFSGLGV-LGRGQLFCCFHEEHLKEATKLYELLIAA

MgiHcB -------------DKLRGLGV-LGRGQIFSCFFEPHLKEATQLYEALISA

EbaHcC -------------PSLIGLGI-LGRGELFNCFHPEHLNEARILYEALIAA

MgiHcC -------------PKLQGLGI-LGRGQLFSCFHQAHLEEAIKLAEVFILA

EbaHcD -------------DRLKSFPT-LQRGHLFSCFNEIHLQESQHLYETLYGA

MgiHcD -------------PRLHDVGI-LERGHLFSCFHEAHLHEAQELYELLWEA

EbaHcE -------------PRLKGLAH-LPRGTLFSCFHEEHLEEATHLYEILFGA

MgiHcE -------------DRLKGVGH-LERGVLFSCFHETHLEEATDLYKILYGA

EbaHcF -------------PRLKTIGK-LPRGTLFSNFHEEHLKEAEALYEVLYAA

MgiHcF -------------PRLKKLGR-LHRGTLFSCFHAEHLEEAKELYEALYKA

EbaHcG -------------ERLREVGI-LHRGTLFSCFHEKHLAEATRLYEILHDA

MgiHcG -------------DLPKSVGI-LPRGTLFSCFHEVHLKEATVLYQYLHDA

CsaHc1 -------------PRLASVGI-LPRGTLFSCFHEKHLNEATELFKILYRA

CsaHc2 -------------PRLAEAGR-LPRGTLFSCFHEKHLKEATELFVILYSA

CsaHc3 -------------PRLAHVGR-LPRGTLFSCFHEKHLAEATELFEILFAA

CsaHc4 -------------PRLADAGH-LPRGTLFSCFHEKHLEEATELFEVLFGA

CsaHc5a -------------PRLVHAGR-LPRGTLFSCFHEKHLEEATELFEILYAA

CsaHc5b -------------PRLVNAGR-LPRGTLFSCFHEKHLEEATELFEILYAA

CsaHc6a -------------PRLAHAGH-LPRGTLFSCFHEKHLEEATELFEILYAA

CsaHc6b -------------PRLAHAGH-LPRGTLFSCFHEKHLEEATELFEILYAA

CsaHc6c -------------PRLAHAGH-LPRGTLFSCFHEKHLEEATELFEILYAA

HauHcB -------------ASLKEIGH-LDTNSVFSLFDERTWPEAKEAVRLMMKA

HauHcA -------------ESLKEIGH-LDTNSVFSLFDERNWPEAKEALTLMMKA

ScoHcA -------------TKLKGLGI-LPQHEIFSLFDERTWPEATKAAEFLMEA

ScoHcD -------------SKLKGLGT-LPRREVFSLFDERNWAEAAKVVELLLEP

ScoHcX -------------EFFQAIGH-LPKKEVFSLFDERHWDEAMTAYEYLYEA

ScoHcC -------------HKLRGLGT-LKKREIFSLFDERNWDEASKVVRLLLDA

ScoHcB -------------DVFKHLGI-LEKHEVFSLFDERQWDEATTAAVYMLTA

SpiHc1 -------------KKLIGIGI-LPKDQVFSLFDERTWDEVSEVLKRFMAA

AgiHc1 -------------KKLIGIGI-LPKDQVFSLFDERTWDEVSEVLKRFLAA

PanHc1 -------------SKLKGVGY-LPKREVFSMFDEREWPEAIEVLKRFLTP

PanHc2 -------------SRLRTLGQ-LPRREVFSLFDARYWNETSQVLDLLIEA

PciHc1 -------------HHV------LEQHHWFSLFNTRQREEALMLVDVLLHA

PciHc2 -------------HRV------LEQHHWFSLFNTRQREEALMLVDVLLHA

NkeHc -------------HRV------LEQHHWFSLFNTRQREEALMLFDVLMSC

GpuHc1 -------------HRV------LEKHHWFSLFNTRQREEALLLFDVLIHS

GroHc1 -------------HRL------LEKHHWFSLFNTRQREEALMLFDVFMHC

CcsHc -------------ERV------LERHHWFSLFNTRQREEALMLFDVFMNC

EpuHc1 -------------HRV------LEHHHWFSLFNNRHREEALMLFDVFMHC

EpuHc2 -------------HRV------LEQHHWFSLFNNRHREEALMLFDALMHC

OscHc1 -------------GHM------LEKKHWFSLFNARHREEAIMLVDVLLSC

OscHc3 -------------GNM------LEKKHWFSLFNTRQREEAIMLIEVLLSC

OscHc2 -------------HRL------LQQKHWFSLFNPRQREEALMLVDALLES

OscHc4 -------------HRL------LQQKHWFSLFNPRQREEALMLVDALLES

PleHc -------------HRL------LEQHHWFSLFNDRQREEALLLVDVLLHS

PleHc2 -------------GRL------LEQHHWFSLFNTRQREEAIMLFEVLIHC

HamHcA -------------HRF------LEQHHWFSLFNPRQREEALMLFDVLMHC

PinHcB -------------HRL------LEQRHWFSLFNTRQREEALMLFAVLNQC

PinHcA -------------HRL------LEQRHWYSLFNTRQRKEALMLFAVLNQC

PvuHc -------------HRL------LEEHHWFSLFNTRQREELAMLFTVLNQC

PelHc1 -------------HRL------LEQHHWFSLFNTRQREEALMLFTVLNQC

PelHc2 -------------HRL------LEQHHWFSLFNTRQREEALMLFTVLNQC

PelHc3 -------------HRL------LEQHHWFSLFNTRQREEALMLFTVLNQC

PelHc4 -------------HRL------LEQHHWFSLFNTRQREEALMLFTVLNQC

CmaHc6 -------------NRL------LKQNHWFSLFNTRQREEALMLYDVLEHS

CmaHc1 -------------HRV------LEQKHWFSLFNERQREEALMMVDVLLNC

CmaHc2 -------------NRV------LEQKHWFSLFNERQREEALMMVDVLLNC

CmaHc3 -------------NRL------LEQHHWFSLFNTRQREEALMLYDILEHS

CmaHc4 -------------NRL------LEQNHWFSLFNTRQRKEALMLYDVLEHS

CmaHc5 -------------NRL------LEQHHWFSLFNTRQREEALMLYDVLEHS

CsaHc -------------HRL------LEQKHWFSLFNTRQREEALMLYDVLEHS

PinHcC -------------GRL------LQKKHWFSLFNTRQREEALMMHRVLMNC

PvaHc1 -------------GKL------LQQRHWFSLFNPRQRHEALMLFDVSIHC

PvaHc -------------GKL------LEQKHWFSLFNTRHRNEALMLFDVLIHC

FchHc1 -------------HRL------LEQNHWFSLLSPRQRHEALMLFDVLIRC

MjaHcL -------------DRL------LQQKHWFSLFNSRQRHEALMLFDVLIHC

MjaHcY -------------GKL------LQQKHWFSLFNTRHRHEALLLFDVLIHC

CjaHc1 -------------HRL------LEQKHWFSLFNERQREEAIMLFEVLIHC

PmaHc2 -------------GKY------LAHDEIFNIFHPHHRRQMVYLFEILYGA

StuHc2 -------------GTT------LPRGEIFNLFNTEHRTSMIHVFEVLFFA

PgrHc2 -------------GKF------LAHDEIFNIFHPQHRRQMIYLFEILYGA

CseHc2 -------------GKL------LERDAIFSLFDARQRAQMVTLFEVLYGA

PamHc2 -------------GKV------LQRDTVFSLFHPRHRYEMVTLFETLYGA

BduHc2 -------------GKL------LHRDGIFGLFNPEHRQQMIVLFETLYGA

HmeHc2 -------------GSL------LSRDTVFSLFNPKHREQMVALFEVLYSA

TdoHc2 -------------GQM------LNRDNIFVLFDPVHRRQMIDVFEILYFA

StuHc3 -------------GDC------LKQSEIFCLFTDHHRHEMILLFEALYYA

StuHc1 -------------HRL------LPRGHVFNLFRPRDREEMVMFFEALFYA

PmaHc1 -------------KTL------LPRGDIFSLFHKEHREEMILLFESFLFA

SamHc1 -------------GSL------LPRGAIFTLFDDTHREQMILLFESLLYA

BduHc1 -------------GQL------LPRGHIFTLFNDKHREQMITVFESLFYA

PamHc1 -------------GHM------LPRGAIFTLFNNKHREEMITVFESFFFA

CseHc1 -------------GRL------LPRGRIFTLFNDRQREQMITIFESLFYA

HmeHc1 -------------GLT------LPRGAIFTLFNDKHREQMITVFESLFFA

CmoHc1 -------------HSL------LPRGQIFTLFDDKHREEMVLLFESMFYA

CacHc1 -------------GSL------LPRGSIFTLFNEKHRSEMILLFETFFYA

TdoHc1 -------------GAL------LPRGEIFTLFRERDLEEMIYLFEALFYA

PgrHc1 -------------KTL------LPRGDIFSLFHKEHREEMILLFESFLFA

ScuHc1 -------------GKL------IKRGEIFNLFNEEHRREMILLFETLFFA

MgeHc1 -------------KQL------LHRGEIFNTFEDKHLEQAILLIETLLSA

PlaPPO QNFDEFLRVATA--VRDVVNEELFVFALLSAILKRPD--TTDLPLPPIQE

AfrPPO KNIDDLFSFAAL--VRDRVNEEMFVYAVMATVFRRPELQASAIKMNNIFE

DmaPPO PTVADMLNLAAHPDVHDAVNEHIFVYALSAALIQRKD--ARSLRLPPIYE

DmePPOA1 PNLRQFVSLSVY--TKDRVNPVLFQYAYAVAVAHRPD--TREVPITNISQ

DmePPO2 RSVDDLQSVAVY--ARDRVNPVLFNYALSVALLHRPD--TQGLDLPSFSQ

PseHc KTFVDFKNAAMH--YKNIVNPMLYAFVFSAAIINRRD--TDTFPVPSLDQ

EpiHc1 KDYKGFLENAAS--LRPVVNEILFVYCFSVALYQSPH--TTNLLAPPIWQ

EspHc1 KDFDDFYKLSQQ--ARSFVNVELFAYSLSVAILHRDD--CKGIQLPSIQE

PimHc2 KDFDDFLNLSQQ--AKDIVNEGLFVFAVSVALLHRED--TRGIHIPPIQE

PimHc3A KDFDDFIHLASQ--AKEIVNATLFAFATEVAVLHRED--CKGLSVPPIQE

PimHc3B KDFDDFIHLAEQ--ARTFVNEGLFAFACEVAILHRSD--CNGLSIPPIQQ

PimHc3C ETFDEFLDLCHQ--ARDFLNEGLYVYAVSVAILHRDD--CRGITLPPIQE

PimHc4 KDFDDFLLLAKQ--ARDFVNEGLFAYAVSVAILHRDD--CKGVTVPPIQE

PimHc5A KDFADLIQLSQQ--ARDIVNEGLFVFAVSVAVLHRED--CKGVTVPPIQE

PimHc5B ETFDEFMDLCHQ--ARDFVNEGLYVYAVSVAILHRAD--CRHVTLPPIQE

PimHc6 KDFDDFIHLSEQ--ARQIVNEGLFVYAVSVAVLHRKD--CVGVSVPPIEE

AauHc6 KDFNDFIHLCEQ--ARQIVNEGMFVYAVSVAVLHRED--CKGITVPPIQE

LpoHcII GDFNDFIEIAKE--ARTFVNEGLFAFAAEVAVLHRDD--CKGLYVPPVQE

LpoHcIIIa KDFDDLMHLLEQ--ARNTVNEGMFVYAATVAVLHRDD--CRGVTVPPIEE

LpoHcIIIb KDFDDFIHLCEQ--AKEVVNEGMFAYAVSVAVLHRDD--CKGLAVPPIQE

LpoHcIV KNFDDFIHLCEE--ARQIVNEGMFVYAASVAVLHRDD--CKGLAVPPIQE

LpoHcVI KTFEEFLDLCHQ--VRDFVNEGLYVYAVSVAILHRPD--CKGVSLPPIQE

CroHcVI KTFEEFLDLCHQ--VRDFVSEGLYVYAVSVAILHRPD--CKGVSLPPIQE

CroHcV DTFEEFLDLCHQ--VRDFVNESLYVYALSVALLHRPD--CKGITLPPIQE

CroHcIV KNFDDFIHLCEE--ARQIVNEGMFVYAVSVAILHRDD--CKGLAVPPIQE

CroHcIIIb EDFDDFIHLCEQ--AKDVVNEGMFAYAISVAVLHRND--CKGLALPPIQE

CroHcIIIa KDFDDLIHLLEQ--ARDTVNEGMFVYAATVAVLHRDD--CRGVTVPPIEE

CroHcII GDFDDFIEVAKE--ARTFVNEGLFAFACEVAVLHRDD--CKGLHVPPVQE

CroHcI KNFDDFLLLCKQ--ARDFVNEGMFAYAVSVALLHRDD--CRGLIIPPVEE

AgoHcA ADFDDFVSLAKQ--ARSFMNSTLFAFAAEVALLHRED--CRGIIVPPVQE

AgoHcB ESFEEFLDLCHQ--AREYVNEGLYVYAVSVAILHRQD--CRGVSLPPVQE

AgoHcC ETFDEFIDLCHQ--CRDFVNEALFVYSLSVAILHRPD--CHGITLPPIQE

AgoHcD KDFDDFIAVATN--ARAIVNEGLYAFALSVAILNRDD--CNGVVLPPIQE

AgoHcE KDFDEMINLAKQ--ARTFVNEGLFVYAVSVAILHRDD--CKGIVVPAIQE

AgoHcF DSFADFIHLAQQ--ARDLVNEGLFVYSASVAILHRDD--CRGVTVPPIQE

AgoHcG ASFNDFIHLARE--ARDVVNEGLFAYALSVAVIHRDD--CKGVTLPPIQE

AgoHcX RDFDEFLNLAFS--ARESVNEGLFVYAVSWSVLNCSL--CQGLLVPPIHE

EcaHcA ANFDDFVSLAKQ--ARSFMNSTLFAFSAEVALLHRED--CRGVIVPPVQE

NinHcA KDFDDFVLLAEQ--ARTFVNSTLFAFAAEVAILHRAD--SRGIIVPPIQE

EcaHcB ESFEEFLDLCHQ--AREYVNEGLYVYAVSVAILHRQD--CRGVSLPPVQE

NinHcB PTFEEFLDLCHQ--CRDYVNEGLFAYAVSVAILHRKD--CRGVNLPPVQE

EcaHcC ETFDEFIDLCHQ--CRDFVNEALFVYSLSVAILHRPD--CHGISLPPIQE

EcaHcD KNFDDFIALATN--ARAVVNEGLYAFAMSVAILSRDD--CNGVVLPPIQE

NinHcD KDFGDFINLAKQ--AREIVNEGLFAFALSVTVLHRDD--CRGVILPPIQE

EcaHcE KDFDEVINLAKQ--SRTFVNEGLFVYAVSVALLHRDD--CKGIVVPAIQE

NinHcE KDFEDFINLAKQ--ARSFANEGLFVYAASVAILHRDD--CRGVTVPPIQE

EcaHcF DSFADFIHLAQQ--ARDIVNEGLFVYSVSVAILHRDD--CRGVTVPPIQE

NinHcF KDFEDFMRLAEQ--ARDIVNEGMFVYSTSVALLHRDD--CRGVTVPPIQE

EcaHcG ESFNDFLQLARE--ARDVVNEGLFAYAFSVAVIHRDD--CKGVTLPPIQE

NinHcG ADFADFLNLAKQ--ARDVVNEGLFVYALSVAVVHRDD--CRGVTLPPIQE

TtrHcA KNFDDFLLLCKQ--ARDFVNEKMFAYAVSVALLHRDD--CRGLIIPPVEE

EbaHcA KDFDDFIHLAEQ--ARQFVNSTLFAFAAEVALLHRQD--CKGLSVPPIQE

MgiHcA KSFDDFIHLAEQ--ARTIVNPSLFAFATEVALLHRPD--CKGLSVPNIQE

EbaHcB ETFDEFLDLCHQ--SRDFVNEGLYIYAVSVAVLHRSD--CHGITLPPIEE

MgiHcB ETFDEFLELCHQ--ARDFVNEGLYTYAVSVAVLHRPD--CRGVSLPPLQE

EbaHcC HSFDDFLDLSHQ--ARDYVNQGLFLYAVSVAILHRDD--CRGVSLPPIQE

MgiHcC KTFSEFLDLAYQ--ARDYVNEGLYVFSLSVAVLHRDD--CRGVSLPPIQE

EbaHcD KDFEDFMHLCEQ--ARDIVNEGLFVYSVSVAVLHRDD--CKGVTVPPIQE

MgiHcD KDFSDFIHLCKQ--AREIVNEGLFVFAVSVAVLHRDD--CHGVRVPPIQE

EbaHcE KDFEDFMILAKQ--AHDLVNEGLFVYAISVAILHRED--CHGVVIPPIEE

MgiHcE KDFDDFMGLAKQ--ARDFVNEGLFTYAATVAITHRED--CRKLKVPPIQE

EbaHcF KDFEDFIHLAEQ--ARNVVNEGLFVFAVSIAVLHRDD--CRGVTVPPIQE

MgiHcF KDFNDFYHLAEE--AREVVNEGLYVFAMTVAILHRSD--CDGIIVPPIQE

EbaHcG KDFEDFFALAKQ--ARDIVNEGLFVYAVSVAVLHRDD--CKGVTVPPIQE

MgiHcG KDFDEFIKRAKL--ARDTVNEGLFTYSLSVALLHRDD--CKGVHIPPIQE

CsaHc1 NDFDDFLKLATQ--ARDVANEGLYVYALSVAVAHRDD--CKGITLPPIQE

CsaHc2 NDFDDFIKLATQ--AREIVNEGLYVYVLSVAVVHRDD--CRGITLPPIQE

CsaHc3 NDFDDFIKLATQ--ARNIVNEGLFVYVLSVAVVHRDD--CRGITLPPIQE

CsaHc4 NDFDDFIKLATQ--ARDIVNEGLFVYAFSVAVVHRDD--CKGVTLPPIQE

CsaHc5a KDFDDFIKLSTQ--ARDVVNEGLFVYVFSVAVVHRDD--CRGVTLPPIQE

CsaHc5b KDFDDFIKLSTQ--ARDVVNEGLFVYVFSVAVVHRDD--CRGVTLPPIQE

CsaHc6a NDFDDFVHLARQ--ARDIVNEGLFVYVLSVAVAHRDD--LKGVTLPPIQE

CsaHc6b NDFDDFVHLASQ--ARDIVNEGLFVYVLSVAVAHRDD--LKGVTLPPIQE

CsaHc6c NDFDDFVHLASQ--ARDIVNEGLFVYVLSVAVAHRDD--LKGVTLPPIQE

HauHcB ANFDEFITIATD--LSHHMNDDMFLFCFNVAIVHRDD--TSGVRPEFHFN

HauHcA ATFEDFITVAKD--LSHHMNDDMFLFVFNVAIVHRDD--TSGVRPEFHFK

ScoHcA TDFEHFIQRADV--LRHRINEDMFMYALNVAVLHRKD--TRGVQVPRIHK

ScoHcD KTFREFIHLADI--IHHRVNEDLFLYALSVAIAHRPD--CQGVQVPRVLD

ScoHcX ETLDDFIDIAKI--LYLHLNEDMFYYVFSFAVLYRKD--TRNVRLPQVHD

ScoHcC KDFDDFIDVAEV--IRLRVNEELFLYAFSVAVMHRGD--TQGLQVPRIHD

ScoHcB PSFDEFIDRAEI--VRHRINEDMFYYAFSVAAVHRDD--TRGINLPRIHE

SpiHc1 PTFEDFIKVAET--LYPHVNEDLFLFGLSVAIVHRPD--ANGVHVPRVHE

AgiHc1 PTFEDFIKVAET--LYPHVNEDLFLFGLSVAIVHRPD--ANGVHVPRVHD

PanHc1 PTFEEFIKVAET--LYPRMNEDLFFYCFSVAIVNRPD--AQGVRVPRVQD

PanHc2 KTFDDFIKRSEV--IQPLVNEELFFYAFSVAFLHRQD--THGLHVPRVHY

PciHc1 TDSDTFFHCAAY--FREKMNEGEFIYAIYVAVTHSPV--TSDLILPPLYE

PciHc2 ADFDTFEHSAAY--FRERINEGEFVYAIYVAVTHSPL--TDHVVLPPLYE

NkeHc NSWDCFKNNAAF--FREKMNEGEFIYAIYVAVIHSPL--TDGVALPPLYE

GpuHc1 KTWETALNNAAY--FREKMNEGEFVYALYAAVIHSKL--GAGIVLPPLYE

GroHc1 KTWEAALNNAAY--FREQMNEGEFVYALYAAVIHSEL--GAGIVLPPLYE

CcsHc KTWKAAMGNAAY--FRHSMNEGEFVYAFYAAVIHSAL--GKGIVLPPLYE

EpuHc1 KDWATAIANAAY--FREKMNEGVFVYAVYTAVIHHDL--GKDLVLPPLYE

EpuHc2 NDWHTAVSAAAY--FREKLNEGVFVYGLYTAVIHADL--GQGLVLPPLYE

OscHc1 TDFEYFKKHAAY--FREHMNEGEFVYALYVAVTHSEV--TSGVILPPLYE

OscHc3 TDFEYFRNHAAY--FREHMNEGEFVYALYVAVTHSET--TTGVILPPLYE

OscHc2 TDFDHFKNTAAY--FRERMNEGEFVYALYVAVTHSDL--CHGVILPPLYE

OscHc4 TDFDHFKNTAAY--FRERMNEGEFVYALYVAVTHSDL--CDGVILPPLYE

PleHc TDLEAFKNNAAY--FREHTNEGEFVYALYVAVTHSDL--TPHVVLPPLYE

PleHc2 KSWVCFVKNAAY--FREHMNEGEFVYALYVAVIHTDL--GHGIVLPPLYE

HamHcA KSWECFVDNAAF--FRERMNEGEFAYALYTAVIHSEL--GQGIVLPPLYE

PinHcB KEWYCFRSNAAY--FRERMNEGEFVYALYVSVIHSKL--GDGIVLPPLYE

PinHcA KEWYCFRSNAAY--FRERMNEGEFVYALYVSVIHSKL--GDGIVLPPLYE

PvuHc KEWDFLNNNAAF--FRERMNEGEFVYALYVSVIHSKL--GDGIVLPPLYQ

PelHc1 KDWNCFLNNAAF--FRERMNEGEFVYALYVSVIHSKL--GDGIVLPPLYE

PelHc2 KNWNCFRNNAAF--FRERMNEGEFVYALYVSVIHSKL--GDGVVLPPLYE

PelHc3 KDWNCFLNNAAF--FRERMNEGEFVYALYVSVIHSKL--GDGIVLPPLYE

PelHc4 KDWNCFLNNAAF--FRERMNEGEFVYALYVSVIHSKL--GDGIVLPPLYE

CmaHc6 TDWSTFAGNAAF--FRVSMNEGEFVYALYAAVIHSEL--TQHVVLPPLYE

CmaHc1 MDFETFQGNAAY--FREHMNEGEFVYAIYVAVTHSEL--MQGVVLPPMYE

CmaHc2 MDFETFQGNAAY--FREHMNEGEFVYAIYVAVTHSEL--MQGVVLPPMYE

CmaHc3 TDWDTFAGNAAF--FRVRMNEGEFVYALYAAVIHSEL--TQHVVLPPIYE

CmaHc4 TDWSTFAGNAAF--FRVHMNEGEFVYALYAAVIHSEL--TQHVVLPPLYE

CmaHc5 TDWSTFAGNAAF--FRVRMNEGEFVYALYAAVIHSEL--TQHVVLPPLYE

CsaHc TDWETFAGNAAY--FRVRMNEGEFVYAIYAAVIHSPL--TEHVVLPPLYE

PinHcC KNWHAFVSNAAY--FRTNMNEGEYLYALYVSLIHSGL--GEGVVLPPLYE

PvaHc1 KDWNTFVSNAAY--FRQKMNEGEFVYALYVAVIHSPL--TEHVVLPPLYE

PvaHc KDWASFVGNAAY--FRQKMNEGEFVYALYVAVIHSSL--AEQVVLPPLYE

FchHc1 KDWDTFVSNAAY--FRQRMNEGEFVYALYVAVIHSPL--AEHVVLPPLYE

MjaHcL KDWDTFVSNAAY--FRQRMNEGEFVNALYVAVIHSSL--AEYVVLPPLYE

MjaHcY NDWAGFVGNAAY--FRQKMNEGEFVYAVYVAVIHSPL--AEHVVLPPLYE

CjaHc1 KDWDTLVGNAAY--WRHIMNEGEFVYAIYVTVIHHPL--AEHVVLPPLYE

PmaHc2 KDFDSFFNMAVW--ARDHMSPRMFLYAFSVAVLHRTD--CKGITLPPAYE

StuHc2 KDFDTFFKTAVY--ARDRVNEFLFYYAFSVAAVRRSD--CEGLQLPPPYE

PgrHc2 KDFDTFFNMAVW--ARDHMSPRMFLYAFSVAVLHRAD--CKGITLPPAYE

CseHc2 TDYDTFLKAAAW--ARDRTNPRQFLYAFSVALLHRED--CRGVILPPAYE

PamHc2 TDYDTFFRTAVY--ARDRLNPRQFLYAFSVALLHRQD--CRGICLPPAYE

BduHc2 SDWDTFFKTAVW--ARDRTNPRQFLYAFSVALLHRED--CRGIKLPPQYE

HmeHc2 VDYDTFFNTAVW--ARDRMNPRQFLYSFSVALLHRPD--CRGLTLPPAYE

TdoHc2 NDYDTFFKTAVW--LRDRMNPRQFLYAFSVALLHRKD--CRGFTLPPAYE

StuHc3 KDWGTFMKMARW--ARVHLNGALFVYSVKVALLHRQD--TYGIRLTPAYE

StuHc1 KTWDTFYKIACW--ARDKINENQFLYAMYVAVHHRHD--CKGVLLPPQYE

PmaHc1 QDWDTFFKTAVW--ARDRINEGQFVYALSVAVLHRED--CKGIILPPAYE

SamHc1 NDWETFLRTAAW--ARDRVNEGQFVYALCVAVLHRED--TRGVVLPPPYE

BduHc1 QDWDTFYRTACW--ARDRVNEGQFVYALSVAVLHRED--TRGIVLPPAYE

PamHc1 EDWDTFYRTACW--ARDRVNEGQFIYALSLAVLHRED--TRGIVLPPAYE

CseHc1 EDWDTFYRTACY--VRDRVNEGQFVYALSVAVLHRED--TRGMVLPPAYE

HmeHc1 QDWDTFLRTACW--CRDRVNEGQFVYALSVAVLHRED--TRGIVLPPPYE

CmoHc1 LDWDTFYKTACW--ARDRINEGQFIYALSVAVIHRDD--AKGIVLPPSYE

CacHc1 NDWDLLFKTACW--ARDRINEGQFVYALSVAVLHRSD--THGIVLPPPYE

TdoHc1 NDWNTFLSTACW--ARDRINEGQFVYALSVAVLHRED--TKGVVLPPAYE

PgrHc1 QDWDTFFKTAVW--ARDRINEGQFVYALSVAVLHRDD--CKGIILPPAYE

ScuHc1 KDWDTFHKTACW--ARDKINEGQFVYALSVATLHRAD--TRGIRLPPAYE

MgeHc1 NSWDTLYRTACW--ARDKVNEGQFVYALSVTVMHSDF--LRGIILPPPYE

PlaPPO IIPYWYFPSEVFTRAL----------------------------------

AfrPPO LFPDRIIGSDAIAKA-----------------------------------

DmaPPO IFPGKFFETKVRSEA-----------------------------------

DmePPOA1 IFPSNFVEPSAFRDAR----------------------------------

DmePPO2 TFPDRFIDSQVIRKMR----------------------------------

PseHc ITPSHFYNEKIIHKSRRKLRKRRRKEMRKFVKRRRDYKKSRGRKEHFNAR

EpiHc1 TMPYDFFDQRVENQI-----------------------------------

EspHc1 IFPDRFVPFETIYNAF----------------------------------

PimHc2 IFPDRFITTETINVAL----------------------------------

PimHc3A VFPDKFINAVTLNKAY----------------------------------

PimHc3B IFPDRFVPANTMIEVL----------------------------------

PimHc3C IFPEKFVNVETLNSAH----------------------------------

PimHc4 VFPDRFIPCETINDAI----------------------------------

PimHc5A IFPDRFIPAETVNQAL----------------------------------

PimHc5B VFPEKFIPAETLFKAF----------------------------------

PimHc6 IFPDRFIPTETINRAT----------------------------------

AauHc6 VFPDRFVPAETINRAN----------------------------------

LpoHcII IFPDKFIPSAAINEAF----------------------------------

LpoHcIIIa VFPDRFIPVETIRHAD----------------------------------

LpoHcIIIb IFPDRFIPAETVTEAI----------------------------------

LpoHcIV IFPDRFIPAETITQAM----------------------------------

LpoHcVI IFPDKFVTVQTLYDAF----------------------------------

CroHcVI IFPDKFMPVQTMYDAY----------------------------------

CroHcV IFPDKFVPVQTFNNAL----------------------------------

CroHcIV IFPDRFIPAETITQAM----------------------------------

CroHcIIIb IFPDRFIPAETITEAI----------------------------------

CroHcIIIa VFPDRFIPAETIRHAD----------------------------------

CroHcII IFPDKFIPSIAINEAF----------------------------------

CroHcI VFPDRFIPVETIREAQ----------------------------------

AgoHcA VFADRFIPADTLIRAF----------------------------------

AgoHcB VFPDKFVPSETLFTAF----------------------------------

AgoHcC IFPDKFVPVETIYKAF----------------------------------

AgoHcD VFPDRFVPAETINRAL----------------------------------

AgoHcE IFPDRFVPTETINLAV----------------------------------

AgoHcF IFPDRFVPAETVNQAV----------------------------------

AgoHcG VFPDRFVPAETINLAM----------------------------------

AgoHcX IFPDKFVPSVTINRAI----------------------------------

EcaHcA VFADRFIPADSLIKAF----------------------------------

NinHcA IFADRFVPADTLIRAF----------------------------------

EcaHcB VFPDKFVPSETLFKAF----------------------------------

NinHcB IFPDKFVPVETLYEAY----------------------------------

EcaHcC IFPDKFVPVETIYKAF----------------------------------

EcaHcD VFPDRFVPAETINRAL----------------------------------

NinHcD VFPDKFIPAETINRAL----------------------------------

EcaHcE IFPDRFVPTETINLAV----------------------------------

NinHcE IFPDRFVPSETISLAL----------------------------------

EcaHcF IFPDRFVPAETVNQAV----------------------------------

NinHcF IFPDRFIPSETINQAI----------------------------------

EcaHcG VFPDRFVPSETINLAM----------------------------------

NinHcG VFPDRFIPAETINLAS----------------------------------

TtrHcA VFPDRFIPVETIREAQ----------------------------------

EbaHcA VFAERFIPAASLIRAF----------------------------------

MgiHcA VFSDKFIPAATLIRAF----------------------------------

EbaHcB VFPDRFVPVEALFKTF----------------------------------

MgiHcB VFPDKFIPGETLNKAY----------------------------------

EbaHcC VFPNKFIPAETLSKAL----------------------------------

MgiHcC VFPDKFVPVETMYNAY----------------------------------

EbaHcD IFPDRFIPAETINLAL----------------------------------

MgiHcD IFPDRFIPAETINRAL----------------------------------

EbaHcE IFPDRFVSAETINHAV----------------------------------

MgiHcE IFPDRFVSAESIVHAA----------------------------------

EbaHcF VFPDRFIPAETINQAQ----------------------------------

MgiHcF IFPDRFIPAESLNRAL----------------------------------

EbaHcG IFPDRFIPAETINLAS----------------------------------

MgiHcG IFPDRFIPAETINLAR----------------------------------

CsaHc1 VFPDRFIPSETINLAM----------------------------------

CsaHc2 VFPDRFIPAETINLAA----------------------------------

CsaHc3 VFPDRFVPAETINLAI----------------------------------

CsaHc4 VFPDRFVPAETINLAT----------------------------------

CsaHc5a VFPDRFIPAETINLAT----------------------------------

CsaHc5b VFPDRFIPAETINLAT----------------------------------

CsaHc6a VFPDRFVPAETINLAI----------------------------------

CsaHc6b VFPDRFVPAETINLAI----------------------------------

CsaHc6c VFPDRFVPAETINLAI----------------------------------

HauHcB SFPDKYIRHDQIVEIR----------------------------------

HauHcA TFPDKYIRHDQIVEMR----------------------------------

ScoHcA IYPDKFLKQDILVEVR----------------------------------

ScoHcD IYPDKFLRKEVIHKIK----------------------------------

ScoHcX VYPDKFLKTDIINKIK----------------------------------

ScoHcC IFPDKFLKEDVIHRLL----------------------------------

ScoHcB IYPDKFLKHKVIVEVK----------------------------------

SpiHc1 VYPDKFLTNDVLINIR----------------------------------

AgiHc1 VYPDKFLTNDVLINIR----------------------------------

PanHc1 VYPDKFFEHYLLVSIK----------------------------------

PanHc2 VFPDKFLKTEVIQKMK----------------------------------

PciHc1 VTPHMFTNSEIINKAY----------------------------------

PciHc2 VTPHMFTNSEIINKAY----------------------------------

NkeHc VTPHLFTNTEVIQQAY----------------------------------

GpuHc1 VTPHLFTNSEVIQKAY----------------------------------

GroHc1 VTPHSFTNSEVIQKAY----------------------------------

CcsHc VTPHMFTNSEVISKAY----------------------------------

EpuHc1 ITPHLFTNSEVINKAY----------------------------------

EpuHc2 VTPHLFTNSEVINKAY----------------------------------

OscHc1 VTPHLFTNSEVIQRAY----------------------------------

OscHc3 VTPHMFTNSEAIQRAY----------------------------------

OscHc2 VTPHMFTNSEVIQKAY----------------------------------

OscHc4 VTPHMFTNSEVIQKAY----------------------------------

PleHc VTPHLFTNSEVIDQAY----------------------------------

PleHc2 VTPHLFTNSEVINKAY----------------------------------

HamHcA ITPHMFTNSEIIHKAY----------------------------------

PinHcB ITPHMFTNSEVIDKAY----------------------------------

PinHcA ITPHMFTNSEVIDKAY----------------------------------

PvuHc ITPHMFTNSEVIDKAY----------------------------------

PelHc1 VTPHMFTNSEVIDKAY----------------------------------

PelHc2 VTPHMFTNNEVIDKAY----------------------------------

PelHc3 VTPHMFTNNEVIDKAY----------------------------------

PelHc4 VTPHMFTNSEVIDKAY----------------------------------

CmaHc6 VTPHLFTNSEVIQEAY----------------------------------

CmaHc1 VTPHMFTNSEVIDKAY----------------------------------

CmaHc2 VTPHMFTNSEVIDKAY----------------------------------

CmaHc3 VTPHLFTNSEVIQEAY----------------------------------

CmaHc4 VTPHLFTNSEVIQEAY----------------------------------

CmaHc5 VTPHLFTNSEVIQEAY----------------------------------

CsaHc VTPHLFTNSEVIQAAY----------------------------------

PinHcC VTPHMFTNSEVIHEAY----------------------------------

PvaHc1 VTPHLFTNSEVIESAY----------------------------------

PvaHc VTPHLFTNSEVIEEAY----------------------------------

FchHc1 VAPHLFTNSEDIEAAY----------------------------------

MjaHcL VTPHLFTNSEVIEAAY----------------------------------

MjaHcY ITPHPFTNSEVIEEAY----------------------------------

CjaHc1 VTPHMFTNSEVIQEAY----------------------------------

PmaHc2 ITPDMFLTTDVMRKAY----------------------------------

StuHc2 IFPHFFVTSDVIRSAY----------------------------------

PgrHc2 ITPDMFLTTDVMRKAY----------------------------------

CseHc2 ITPHMFLTTDVVRKAY----------------------------------

PamHc2 ITPHMFLTTDVVRKAY----------------------------------

BduHc2 ITPHVFLTTDVVRKAY----------------------------------

HmeHc2 ITPHMFLTTDVVREAY----------------------------------

TdoHc2 ITPHMFLTTDVVRQAY----------------------------------

StuHc3 LNPHMFVTNDAITKAY----------------------------------

StuHc1 IYPQLFVNNDVIQEAY----------------------------------

PmaHc1 IYPHMFVNSEVINSAY----------------------------------

SamHc1 IYPHLFVNSEVIHAAY----------------------------------

BduHc1 IYPHLFVNSEVIHAAY----------------------------------

PamHc1 IYPHLFVNSEVIHAAY----------------------------------

CseHc1 VYPHLFVNSDVIHAAY----------------------------------

HmeHc1 IYPHLFVNSEVIHAAY----------------------------------

CmoHc1 IYPHLYVNSEVIHAAY----------------------------------

CacHc1 IYPHLFVNSEVIHAAY----------------------------------

TdoHc1 IYPHLFVNSEVIHQAY----------------------------------

PgrHc1 IYPHLFVTSEVIDSAY----------------------------------

ScuHc1 TYPHLFVTSQVIHEAY----------------------------------

MgeHc1 IYPHLFVNSEVIHEAY----------------------------------

PlaPPO -------------------------------------------RRVRDAQ

AfrPPO -------------------------------------------KETIKQS

DmaPPO -------------------------------------------QINVQQK

DmePPOA1 -------------------------------------------QEASVIG

DmePPO2 -------------------------------------------EESF--V

PseHc HGKKSHNDKSIDHEKKAKEGHHQGSKNQREHEGSSHHGKHRHKRQADVGS

EpiHc1 -------------------------------------------NGIIEST

EspHc1 -------------------------------------------RVAQ--G

PimHc2 -------------------------------------------KADS--R

PimHc3A -------------------------------------------FDAY--S

PimHc3B -------------------------------------------KKAS--T

PimHc3C -------------------------------------------SKVYDVH

PimHc4 -------------------------------------------KEVA--N

PimHc5A -------------------------------------------KSDR--Q

PimHc5B -------------------------------------------KEAK--L

PimHc6 -------------------------------------------KEAS--N

AauHc6 -------------------------------------------KEAS--N

LpoHcII -------------------------------------------KKAH--V

LpoHcIIIa -------------------------------------------KEAA--A

LpoHcIIIb -------------------------------------------KADH--N

LpoHcIV -------------------------------------------KDDH--H

LpoHcVI -------------------------------------------KEFW--Q

CroHcVI -------------------------------------------KEAW--L

CroHcV -------------------------------------------KEAF--T

CroHcIV -------------------------------------------KDDH--H

CroHcIIIb -------------------------------------------KDDH--N

CroHcIIIa -------------------------------------------KEVT--A

CroHcII -------------------------------------------KKAH--I

CroHcI -------------------------------------------KEVE--L

AgoHcA -------------------------------------------TLAT--T

AgoHcB -------------------------------------------KEVR--L

AgoHcC -------------------------------------------KEAT--R

AgoHcD -------------------------------------------KVDK--L

AgoHcE -------------------------------------------KEAA--N

AgoHcF -------------------------------------------KADL--K

AgoHcG -------------------------------------------KEAK--N

AgoHcX -------------------------------------------KQYL--D

EcaHcA -------------------------------------------TLAT--T

NinHcA -------------------------------------------SVAT--T

EcaHcB -------------------------------------------KEVR--L

NinHcB -------------------------------------------KETK--L

EcaHcC -------------------------------------------KEAT--R

EcaHcD -------------------------------------------KVDK--V

NinHcD -------------------------------------------KADK--K

EcaHcE -------------------------------------------KEAA--N

NinHcE -------------------------------------------KEVT--N

EcaHcF -------------------------------------------KADL--K

NinHcF -------------------------------------------KADL--R

EcaHcG -------------------------------------------KEAK--N

NinHcG -------------------------------------------KEAR--N

TtrHcA -------------------------------------------KEVE--L

EbaHcA -------------------------------------------TEAT--T

MgiHcA -------------------------------------------IEAS--V

EbaHcB -------------------------------------------HEST--L

MgiHcB -------------------------------------------YEST--K

EbaHcC -------------------------------------------VDAE--Q

MgiHcC -------------------------------------------KQAT--I

EbaHcD -------------------------------------------KADK--R

MgiHcD -------------------------------------------KADL--Q

EbaHcE -------------------------------------------KEAA--N

MgiHcE -------------------------------------------KEAI--N

EbaHcF -------------------------------------------KADL--K

MgiHcF -------------------------------------------KADI--N

EbaHcG -------------------------------------------KEAT--L

MgiHcG -------------------------------------------KEVY--R

CsaHc1 -------------------------------------------KEAK--N

CsaHc2 -------------------------------------------KEAK--N

CsaHc3 -------------------------------------------KESK--N

CsaHc4 -------------------------------------------KEAK--N

CsaHc5a -------------------------------------------KEAK--N

CsaHc5b -------------------------------------------KEAK--N

CsaHc6a -------------------------------------------KESK--N

CsaHc6b -------------------------------------------KESK--N

CsaHc6c -------------------------------------------KESK--N

HauHcB -------------------------------------------HAVECGQ

HauHcA -------------------------------------------HAVECGK

ScoHcA -------------------------------------------EKVN---

ScoHcD -------------------------------------------EVSN--E

ScoHcX -------------------------------------------QANY---

ScoHcC -------------------------------------------ELSN--R

ScoHcB -------------------------------------------NSIN---

SpiHc1 -------------------------------------------KQII---

AgiHc1 -------------------------------------------KQII---

PanHc1 -------------------------------------------DKIM---

PanHc2 -------------------------------------------QLTY--N

PciHc1 -------------------------------------------TAKM---

PciHc2 -------------------------------------------TAKM---

NkeHc -------------------------------------------TAKM---

GpuHc1 -------------------------------------------TAQM---

GroHc1 -------------------------------------------TAQM---

CcsHc -------------------------------------------TAQM---

EpuHc1 -------------------------------------------SAKM---

EpuHc2 -------------------------------------------SAKM---

OscHc1 -------------------------------------------TAKM---

OscHc3 -------------------------------------------TAKM---

OscHc2 -------------------------------------------TAKM---

OscHc4 -------------------------------------------TAKM---

PleHc -------------------------------------------AAKM---

PleHc2 -------------------------------------------SAKM---

HamHcA -------------------------------------------TAKM---

PinHcB -------------------------------------------SAKM---

PinHcA -------------------------------------------SAKM---

PvuHc -------------------------------------------SAKM---

PelHc1 -------------------------------------------SAKM---

PelHc2 -------------------------------------------SAKM---

PelHc3 -------------------------------------------SAKM---

PelHc4 -------------------------------------------SAKM---

CmaHc6 -------------------------------------------KAKM---

CmaHc1 -------------------------------------------AAKM---

CmaHc2 -------------------------------------------TAKM---

CmaHc3 -------------------------------------------KAKM---

CmaHc4 -------------------------------------------KAKM---

CmaHc5 -------------------------------------------KAKM---

CsaHc -------------------------------------------KAKM---

PinHcC -------------------------------------------KAQM---

PvaHc1 -------------------------------------------RAKQ---

PvaHc -------------------------------------------RAKQ---

FchHc1 -------------------------------------------RAKQ---

MjaHcL -------------------------------------------RAKQ---

MjaHcY -------------------------------------------RAKQ---

CjaHc1 -------------------------------------------HAKM---

PmaHc2 -------------------------------------------QAKM---

StuHc2 -------------------------------------------KAKM---

PgrHc2 -------------------------------------------QAKM---

CseHc2 -------------------------------------------QAKM---

PamHc2 -------------------------------------------KAKM---

BduHc2 -------------------------------------------QAKM---

HmeHc2 -------------------------------------------KAKM---

TdoHc2 -------------------------------------------QAKM---

StuHc3 -------------------------------------------SAKM---

StuHc1 -------------------------------------------SAKM---

PmaHc1 -------------------------------------------KAKM---

SamHc1 -------------------------------------------KAKM---

BduHc1 -------------------------------------------RAKM---

PamHc1 -------------------------------------------KAKM---

CseHc1 -------------------------------------------RAKM---

HmeHc1 -------------------------------------------KAKM---

CmoHc1 -------------------------------------------KAKM---

CacHc1 -------------------------------------------KAKM---

TdoHc1 -------------------------------------------KAKM---

PgrHc1 -------------------------------------------KAKM---

ScuHc1 -------------------------------------------AAKM---

MgeHc1 -------------------------------------------SAKM---

PlaPPO N---DQEEI-V-LDHP---FAGNELDPERRLAYFREDVDVNSHHWHWHLV

AfrPPO TGDEDEETV-LEINQE---FSALDKNPENRLAYFREDLGVNSHHWHWHLV

DmaPPO KEPATQEPI-I-IDKN---FAATNRVVENKVSYFREDLGINSHHWHWHLI

DmePPOA1 E-SGARVHV-D-IPQN---YTASDREDEQRLAYFREDIGVNSHHWHWHLV

DmePPO2 VQPGSRMPI-T-IPRD---YTASDLDPEHRLWYFREDLGINLHHWHWHLV

PseHc NQEPDQNDI-TQMPLD---HTGDNRNPEHRVSYFREDIGINSHHWGWHLV

EpiHc1 KGAPDKQPI-Q-VILE---PTGNNRDPEFKLSYFREDIAINSHHWHWHVI

EspHc1 G-GGGKDGI-L-VKIA---HTGNILDPEYKLAYYREDLGINGHHWHWHLV

PimHc2 R-T-GEEPI-K-IPIK---ETGNILDREYNLAYFREDVEVNAHHWHWHIV

PimHc3A RTAADDRPL-I-EEEI---HVGNVLDPEYKLAYFREDVGANAHHWHWHVV

PimHc3B KPAADESPI-V-VDVE---STGNILDPEYNLAYYREDVGINAHHWHWHLV

PimHc3C A-EKGKDDI-I-VDWVMPFEHGEVQDPESRLFYYREDIDLNAHHWHWHLV

PimHc4 H---PDKDN-V-VSVQ---ETGNILDPEYKLAYFREDIGTNAHHWHWHII

PimHc5A R-E-SNDPV-V-VKIQ---ETGNILDPEYNLAYFREDIETNAHHWHWHLV

PimHc5B H-E-DEEEV-V-VEVE---QTGNVLDPEYNLAYYREDVGINAHHWHWHLV

PimHc6 H---PDKDT-V-VPVE---ETGNILDPEYKLAYFRENVGINAHHWHWHIV

AauHc6 H---PDQQSIV-VEAE---ETGNILDPEYKLSYFREDIGINAHHWHWHIV

LpoHcII RPEFDESPI-L-VDVQ---DTGNILDPEYRLAYYREDVGINAHHWHWHLV

LpoHcIIIa H---PDKDI-V-VEFE---ETGNILDPEYRLAYYREDVSINRHHWHWHIV

LpoHcIIIb R-Q-DHEAL-T-VVAH---DTGNILNPEQHLAYFREDIETNAHHWHWHIV

LpoHcIV R-K-EHEDL-V-VDIE---ETGNILNPEQRLAYFREDIEVNAHHWHWHLV

LpoHcVI N-PHKEEDV-I-VDMQ---TTGNILDPEYNLAYYREDIGINAHHWHWHLV

CroHcVI N-PNKEEDI-I-VDMQ---STGNILDPEYNLAYYREDIGINAHHWHWHIV

CroHcV E-PDKEKEM-I-VNMQ---ATGNIHDPEYNIAYYREDIGINAHHWHWHLV

CroHcIV R-K-EHEAL-V-VDME---ETGNILNPEQRLAYFREDIEINAHHWHWHLV

CroHcIIIb R-H-DHEVL-T-VEAR---DTGNILNPEQHLAYFREDIETNAHHWHWHIV

CroHcIIIa H---PDKDI-V-VEFE---ETGNILDPEYRLAYYREDVSINRHHWHWHIV

CroHcII RPEFDESPI-L-VDVH---DTGNILDPEYRLAYYREDVGINAHHWHWHLV

CroHcI H---PGKDI-V-VEVE---NTGNILDPEYRLCYFREDLGVNAHHWHWHIV

AgoHcA TQPGDESDI-V-VDVK---DTGNILDPEYKLAYFREDIGVNAHHWHWHIV

AgoHcB H-P-EDEEI-I-VDME---KTGNIKDPEYNLAYYREXIGINAHHWHWHLV

AgoHcC H-MDKNDDI-I-VDME---ATGTIMDPEYNLAYYREDIGINAHHWHWHVV

AgoHcD S-D-PNKDT-V-VPIQ---KTGNIRDPEYNVAYFREDIGINSHHWHWHLV

AgoHcE H---PDQDV-S-VHVV---ETGNILDEEYKLAYFREDVGTNAHHWHWHIV

AgoHcF R-QSPDEDV-V-VEIQ---ETGNILDPEHKLAYFREDIGANAHHWHWHIV

AgoHcG D---PNSDI-V-VDVQ---ETGNILDPEYKLFYFREDVGCNAHHWYWHVV

AgoHcX R-T-TDNPI-I-VTAD---PTGTPLDEEQRLTYFREDIGLNSHHWHWHLV

EcaHcA TQPGDESDI-I-VDVK---DTGNILDPEYKLAYFREDIGVNAHHWHWHVV

NinHcA KPAGDESDV-I-IDVQ---ETGNILDPEYKLAYYREDIGVNAHHWHWHVV

EcaHcB H-P-DDEEI-I-VDIE---KTGNVKDPEYNLAYYREDIGVNAHHWHWHLV

NinHcB H--KEDEDV-I-INIQ---KTGNIMDPEYNLAYYREDIGINAHHWHWHLV

EcaHcC H-ADKTDDI-I-VDME---ATGTIMDPEYNLAYYREDIGINAHHWHWHVV

EcaHcD S-D-PNKDT-V-VPIQ---KTGNIRDPEYNVAYFREDIGINSHHWHWHLV

NinHcD S---TDESK-V-IEIQ---KTGNIIDPEYNLAYFREDIGINAHHWHWHLV

EcaHcE H---PDQDI-S-VHVV---ETGNILDEEYKLAYFREDVGTNAHHWHWHIV

NinHcE H---PDKDI-E-VEIE---STGNILDPEYKMSYFREDVGTNAHHWHWHIV

EcaHcF R-QSSDEDV-L-VEIQ---ETGNILDPEHKLAYFREDIGANAHHWHWHIV

NinHcF R-A-DDSPV-I-VDVI---QTGNILDPEYKLAYFREDIGANAHHWHWHIV

EcaHcG D---PNSDI-V-VDVQ---ETGNILDPEYKLAYFREDIGANAHHWYWHVV

NinHcG N---PTEDV-V-VEIE---DTGNILEPEYKLAYFREDIGINAHHWYWHVV

TtrHcA H---PGKDI---VEVE---NTGNILDPEYRLCYFREDLGVNAHHWHWHIV

EbaHcA RQPGDERDI-V-VDVE---DTGNILDPEYPLAYYREDIAVNAHHWHWHIV

MgiHcA KTPGDESDV-V-VDVK---EHGTILDPEHKLAYYREDVEVNAHHWHWHIV

EbaHcB A-P-PEQDV-E-VHIE---DTGNVLDPEYHLAYFREDVGINAHHWYWHVV

MgiHcB I-P-EDEDV-I-HDMQ---ETGNILDPEYKLAYYREDIGVNVHHWHWHLV

EbaHcC H-PDTEVDI-I-TDME---FVGNILDPEYLLSYYREDIGINAHHWHWHLV

MgiHcC T-D-PEKEI-I-VESE---TTGNVLDPEYNVAYYREDVGINAHHWHWHIV

EbaHcD R-DDAAGGI-V-IPIQ---HTGNILDKEYHLAYFREDIEVNAHHWHWHLV

MgiHcD R---GDQDV-VSVEIE---HTGNIIDPEFKLAYFREDIETNAHHWHWHLV

EbaHcE H---PDKDI-V-VKSE---ETGNILDEEYKLAYFREDVGANAHHWHWHIV

MgiHcE Q---PDQDI-V-VERE---ETGNILDPEHSLTYFREDVGVNAHHWHWHIA

EbaHcF R-T-SDDSI-V-IKIE---QTGNILDSEYKVAYFREDIGINAHHWHWHIV

MgiHcF R-T-SQEPV-V-VEIQ---ETGNIFDPEYHLAYFREDIGANAHHWHWHIV

EbaHcG K---PNEDV-V-VDVE---ETGNILDPEHTLAYYREDIGINAHHWHWHIA

MgiHcG N---PKEDI-V-VPVE---KTGNILDPEYKLAYYREDIETNAHHWHWHIV

CsaHc1 K---TYDDV-V-VEIE---DTGNIRDPEYKLAYFREDVAVNAHHWYWHVV

CsaHc2 H---PGDDI-V-VESE---TTGNIMDPEYKLAYFREDVKVNAHHWYWHVV

CsaHc3 H---PDIDI-E-VEIQ---QTGNIMEPEYKLAYFREDIATNAHHWYWHVV

CsaHc4 H---PGLDV-V-VEIE---KTGNIRDKEYKLAYFREDIGINAHHWYWHVV

CsaHc5a H---SNDDV-V-VPSQ---TTGNILDPEYKLAYFGEDIKVNAHHWYWHVV

CsaHc5b H---PNDDV-V-VPSQ---TTGNILDPEYKLAYFREDIKVNAHHWYWHVV

CsaHc6a H---PDQDI-E-VEIQ---QTGNIMEPEYKLAYFREDIGVNAHHWYWHVV

CsaHc6b H---PDQDI-E-VEIQ---QTGNIMEPEYKLAYFREDIGVNAHHWYWHVV

CsaHc6c H---PDQDI-E-VEIQ---QTGNIMEPEYKLAYFREDIGVNAHHWYWHVV

HauHcB D---------N-IKVLANPHVFNDTDDAHKLLYWLEDLGLNAHHYHWHTV

HauHcA D---------N-IKVLANPHVFNDTDDAHKLLYWLEDLGLNAHHYHWHTV

ScoHcA --HGEEKPV---VDATE--LHQNQLDPNYRLSYFLEDIGMNSHHYHWHVV

ScoHcD GAYLDKVPV---IDATE--VSDNHLDPNQELLYFLEDLGMNSHHHHWHVI

ScoHcX --QGKQHPV---IDATK--EFHDLRNPVSYLHYFLEDIGMNSHHYHWHVM

ScoHcC GEHYDRIPI---IDATQ--ISHNYLDPNSELEYFLEDLGLNSHHHHWHVI

ScoHcB --SGQEDPL---IDATH--EFTDLRDPNSKLHYFLEDVGLNSHHYHWHVI

SpiHc1 --KGHKNPV---VKDTH--YYHNDFDPFRRVDYFTEDLGMNSHHYHWHVL

AgiHc1 --KGHKNPV---VKDTH--YYHNDFDPFRRVDYFTEDPGMNSHHYHWHVL

PanHc1 --TGHKNPV---VNDTH--DFHNHYDPYRRVDYFTEDMGMNSHHYHWHVT

PanHc2 AERYGKSAI---VEGNDLSHIHNYYDTHDELSYFTEDVGMNAHHYHWHVV

PciHc1 ----TQTPG-N-FNME---FSSSKKNPETRVAYFGEDIGMNSHHVHWHMD

PciHc2 ----TQTPG-D-FNME---FTGTNKNEEQRVAYFGEDIGINSHHVHWHMD

NkeHc ----TQTPG-N-FEMG---YTGSQKNPEQRVAYFGEDVGMNSHHTHWHMD

GpuHc1 ----TQTPG-T-FKQD---FTGSKKNPEQRVAYFGEDIGMNVHHVTWHLD

GroHc1 ----TQTPG-N-FKMD---FTGSKKNPEQHVAYFGEDIGMNVHHVTWHLD

CcsHc ----TQTPG-K-FSME---FTGSQKNPEQRVAYFSEDIGLNVHHVTWHMD

EpuHc1 ----TQTPG-K-FHMS---FTGTKKNPEQRIAYFGEDIGLNVHHVTWHMD

EpuHc2 ----TQTPG-K-FRMS---FTGTKKNPEQRVAYFGEDIGLNVHHVTWHMD

OscHc1 ----THTPA-K-LKME---FTGSKKNPEQRVAYFGEDIGMNSHHVHWHMD

OscHc3 ----THTPA-K-LQME---FTGSKKNPEQRVAYFGEDIGMNSHHVHWHMD

OscHc2 ----TQTPT-K-LTME---FTGSKKNPEQRVAYFGEDIGMNSHHVHWHMD

OscHc4 ----TQTPA-K-FEME---FTGSQKNPEQRVAYFGEDIGMNSHHVHWHMD

PleHc ----TQTPG-N-FKME---FTGSKKNREQRGAYFGEDVGLNSHHVHWHMD

PleHc2 ----TQTPG-K-FNMD---FTGTKKNKEQRVAYFGEDIGMNIHHVTWHMD

HamHcA ----TQTPG-R-FEMK---FTGTKKNKEQRVAYFGEDIGLNIHHVTWHMD

PinHcB ----TQKPG-T-FNVS---FTGTKKNREQRVAYFGEDIGMNIHHVTWHMD

PinHcA ----TQKQG-T-FNVS---FTGTKKNREQRVAYFGEDIGMNIHHVTWHMD

PvuHc ----THKEG-T-FNMS---FTGTQKNREQRVAYFGQDIGMNIHHVTWHMD

PelHc1 ----THKEG-T-FNMS---FTGTKKNKEQRVAYFGEDIGMNIHHVTWHMD

PelHc2 ----THEGN-T-FDMS---FTGTKKNREQRVAYFGEDIGMNIHHVTWHMD

PelHc3 ----THKEG-T-FNMS---FTGTKKNKEQRVAYFGEDIGMNIHHVTWHMD

PelHc4 ----THKEG-T-FNMS---FTGTKKNKEQRVAYFGEDIGMNIHHVTWHMD

CmaHc6 ----TQTAA-K-IESH---FTGSKSNPEQRVAYFGEDIGMNTHHVTWHLE

CmaHc1 ----TQTAG-E-FYMS---FTGSKKNPEQRVAYFGEDIGMNSHHVHWHMD

CmaHc2 ----TQTAG-E-FYMS---FTGSKKNPEQRVAYFGEDIGMNSHHVHWHMD

CmaHc3 ----TQTRA-K-IESH---FTGSKSNPEQRVAYFGEDIGMNTHHVTWHLE

CmaHc4 ----TQTAA-K-IESH---FTGSKSNPEQRVAYFGEDIGMNTHHVTWHLE

CmaHc5 ----TQTAA-K-IESH---FTGSKSNPEQRVAYFGEDIGMNTHHVTWHLE

CsaHc ----TQTAS-K-IKSH---FTGSKSNPEQRVAYFGEDIGMNTHHVTWHLE

PinHcC ----TNTPS-K-FESH---FTGSKKNPEQHVAYFGEDVGMNTHHVLWHME

PvaHc1 ----TQKPG-K-FESS---FTGTKKNPEQRVAYFGEDIGMNTHHVTWHME

PvaHc ----KQTPG-K-FKSS---FTGTKKNPEQRVAYFGEDIGLNTHHVTWHME

FchHc1 ----TQTPG-K-FQSS---FTGTKKNPEQRVAYFGEDIGMNTHHVTWHME

MjaHcL ----TQKPG-K-FKSS---FTGTKKNPEQRVAYFGEDIGMNTHHVTWHME

MjaHcY ----TQTPG-K-FKST---FTGTKKNPEQRVAYFGEDIGLNTHHVTWHME

CjaHc1 ----TQTPG-K-FKSS---FTGTKKNKEQRVAYFGEDIGMNTHHVVWHME

PmaHc2 ----TATKT-V-IPMK---FTGSIKNPEQRVAYFGEDIGVNSHHSHWHMD

StuHc2 ----MHTPT-I-IDMH---WTGSIHNPEQRVAYYGEDVGLNSHHSHWHKD

PgrHc2 ----TAQKT-V-IPMK---FTGSIKNPEQRVAYFGEDIGINSHHSHWHMD

CseHc2 ----MRTPT-V-IPMK---FTGSVNNPEQRVAYFGEDIGMNSHHSHWHMD

PamHc2 ----TRTPT-V-IPMK---FTGSVNNPEQRVAYFGEDIGMNSHHSHWHMD

BduHc2 ----TRTPT-V-IPMK---FTGSANNPEQRVAYFGEDIGLNSHHAHWHMD

HmeHc2 ----TRQPV-L-IPMK---FTGSVNNPEQRVAYFGEDIGMNSHHAHWHMD

TdoHc2 ----TRTPK-V-IPMQ---FTGSVRNQEQRVAYFGEDIGMNSHHSHWHMD

StuHc3 ----RNKDA-V-VRVE---FTGTIHNPEQHVAYYGEDIGMNSHHSHWHMD

StuHc1 ----KQVPA-T-IKMH---WTGTIRNPEQHVAYFGEDLGLNSHHSHWHMD

PmaHc1 ----TQTPA-I-IHMN---FTGTIRNPDQWIAYLGEDVGLNSHHAHWHMD

SamHc1 ----RQEPA-V-VHMN---FTGTIRNPEQRVAYLGEDLGMNSHHSQWHMD

BduHc1 ----RQEPA-V-VRMN---FTGTVRNPEQRVAYLGEDVGMNSHHSHWHMD

PamHc1 ----RQEPA-V-VRMN---FTGTIRNPEQRVAYLGEDVGMNSHHSHWHMD

CseHc1 ----RQEPA-V-VRMN---FTGTVRNPEQRVAYLGEDVGINSHHSHWHMD

HmeHc1 ----RQEPA-I-VRMN---FTGTIRNPEQRVAYLGEDVGMNAHHSHWHMD

CmoHc1 ----RQEPA-V-VRMN---FTGTIRNPEQRVAYLGEDLGMNSHHAHWHMD

CacHc1 ----RQEPA-I-VRMN---FTGTIKNPEQRVAYLGEDIGMNAHHAHWHMD

TdoHc1 ----RQEAA-V-VRMN---FTGTIRNPEQRVAYFGEDIGMNSHHSHWHMD

PgrHc1 ----TQTPA-T-IYMN---FTGTVRNPEQWVAYLGEDVGLNSHHAHWHMD

ScuHc1 ----RQEPA-V-IHMN---FTGTVRNPEQRVAYFGEDVGMNSHHAVFHMD

MgeHc1 ----RHHPV-V-IPMN---FTGTIRNPEQRVAYFGEDIGLNTHHHYWHMN

PlaPPO -------YPFTW-------VPQQGEV--KD----RKGELFYYMHQQIMTR

AfrPPO -------FPD---------DEEFK----RD----RRGEMFFYMHHQIIAR

DmaPPO -------FPVEA-------TGEIINP--PD----RRGELFYYMHQQILAR

DmePPOA1 -------YPTTGP------TEVVN----KD----RRGELFYYMHHQILAR

DmePPO2 -------YPFEA-------SDRSIVA--KD----RRGELFYYMHQQVIAR

PseHc -------YPLFWI------NKAGRIL--KD----RKGELFFYMHNQIVKR

EpiHc1 -------YPVGS------------NP--SDKKINRKGELFYYMHEQMLAR

EspHc1 -------YPASWR------SEVMGKK--KD----RKGELFFYMHQQMVAR

PimHc2 -------YPAIWR------SEVTHKK--KD----RKGELFFYMHQQMCAR

PimHc3A -------YPSIWD------PKVLGKK--KD----RRGELFYYMHQQMCAR

PimHc3B -------YPGTWS------ASYFHKD--KD----RKGELFYYMHQQMCAR

PimHc3C -------YPATWN------AAVTGKK--KN----RKGELFYYMHQQMCAR

PimHc4 -------YPATWK------ESVMKKA--KD----RKGELFYYMHQQMCAR

PimHc5A -------YPAKWR------TDVMGKA--KD----RKGELFYYMHQQMCAR

PimHc5B -------YPATWR------AEKLGRR--KD----RKGELFYYMHQQMCAR

PimHc6 -------YPATWD------PAVMGHE--KD----RKGELFFYMHQQMCAR

AauHc6 -------YPATWN------PTVMGKE--KD----RKGELFFYMHQQMCAR

LpoHcII -------YPSTWN------PKYFGKK--KD----RKGELFYYMHQQMCAR

LpoHcIIIa -------YPATWR------PEFMHKE--KD----RKGELFYYMHQQMCAR

LpoHcIIIb -------YPATWD------SKVMHKK--KD----RKGELFFYMHQQMCAR

LpoHcIV -------FPATWR------PEVMGKV--KD----RKGELFYYMHQQMCAR

LpoHcVI -------YPATWR------PEVIGKV--KD----RKGELFYYMHQQMCAR

CroHcVI -------YPATWR------PEVIGKI--KD----RKGELFYYMHQQMCAR

CroHcV -------YPATWR------PEVTGKV--KD----RKGELFYYMHQQMCAR

CroHcIV -------FPATWR------PEVMGKV--KD----RKGELFYYMHQQMCAR

CroHcIIIb -------YPATRN------SKVMHKV--KD----RKGELFFYMHQQMCAR

CroHcIIIa -------YPATWR------PEVMHKE--KD----RKGELFYYMHQQMCAR

CroHcII -------YPSTWD------PKYFSKK--KD----RKGELFYYMHQQMCAR

CroHcI -------YPATWR------SEVMGKT--KD----RKGELFFYMHQQMCAR

AgoHcA -------YPSTYD------PGFFGKV--KD----RKGELFYYMHQQMCAR

AgoHcB -------YPATWR------PEIVHRI--KD----RKGELFFYMHQQMCAR

AgoHcC -------YPSTWD------SVKMHMN--KD----RKGELFFYMHQQMCAR

AgoHcD -------YPAFYD------ADIFGKI--KD----RKGELFYYMHQQMCAR

AgoHcE -------YPATWD------PAFMGRM--KD----RKGELFYYMHQQMCAR

AgoHcF -------YPATWD------VSVMSKV--KD----RKGELFYYMHQQMCAR

AgoHcG -------YPANWD------ADFIGKT--KD----RKGELFYYMHQQMCAR

AgoHcX -------YPVN------------GAT--KD----RKGELFYYMHQQMCAR

EcaHcA -------YPSTYD------PAFFGKV--KD----RKGELFYYMHQQMCAR

NinHcA -------YPSVYD------SKFFGKK--KD----RTGELFYYMHQQMCAR

EcaHcB -------YPATWR------PEVVHRI--KD----RKGELFFYMHQQMCAR

NinHcB -------YPATWR------PEVIGRI--KD----RKGELFFYMHQQMCAR

EcaHcC -------YPSAWD------SVKMHMR--KD----RKGELFFYMHQQMCAR

EcaHcD -------YPAFYD------ADIFGKI--KD----RKGELFYYMHQQMCAR

NinHcD -------YPATYR------PDFFGKV--KD----RKGELFYYMHQQMCAR

EcaHcE -------YPATWD------PAFMGRM--KD----RKGELFYYMHQQMCAR

NinHcE -------YPATWR------PEVMGKL--KD----RKGELFYYMHQQMCAR

EcaHcF -------YPPTWD------ASVMSKV--KD----RKGELFYYMHQQMCAR

NinHcF -------YPATWR------PEIMGKV--KD----RKGELFYYMHQQMCAR

EcaHcG -------YPANWD------AVFTGKT--KD----RKGELFYYMHQQMCAR

NinHcG -------YPANWS------VELTGKV--KD----RKGELFYYMHQQMCAR

TtrHcA -------YPATWR------SEVMGKT--KN----RKGELFFYMHQQMCAR

EbaHcA -------YPSVWN------SEYFGKS--KD----RKGELFYYMHQQMCAR

MgiHcA -------YPSTWD------AKYFGKS--KD----RKGELFYYMHQQMCAR

EbaHcB -------YPATWR------AEVIGRP--KD----RKGELFYYMHQQMCAR

MgiHcB -------YPATWR------PEITGIV--KD----RKGELFYYMHQQMCAR

EbaHcC -------YPATWR------ADITGKT--MD----RKGELFYYMHQQMCAR

MgiHcC -------YPSAWN------AEVTGKT--KD----RKGELFYYMHQQMCAR

EbaHcD -------YPASWR------ASVMGKT--KD----RKGELFCYMHQQMCAR

MgiHcD -------YPANWR------SDFFGKA--KD----RKGELFAYMHQQMCAR

EbaHcE -------YPATWK------PLVMKQT--KD----RKGELFYYMHQQMCAR

MgiHcE -------YPATWD------ASVMGRE--KD----RKGELFYYMHQQMCAR

EbaHcF -------YPTTWR------PEVMHKV--KD----RKGELFYYMHQQMCAR

MgiHcF -------YPGTWD------PEVMHIT--KD----RKGELFYYMHQQMCAR

EbaHcG -------YPATWN------PSHMGAV--KD----RKGELFFYMHQQMCAR

MgiHcG -------YPATWD------AEKMGHI--KD----RKGELFYYMHQQMCAR

CsaHc1 -------YPANWD------ESLTGKV--KD----RKGELFYYMHQQMSAR

CsaHc2 -------YPANWD------EGLTAKV--KD----RKGELFYYMHQQMSAR

CsaHc3 -------YPANWD------EGLTGKV--KD----RKGELFYYMHQQMCAR

CsaHc4 -------YPANWS------EELTGKI--KD----RKGELFYYMHQQMCAR

CsaHc5a -------YPANWN------EELTDKV--KD----RKGELFYYMHQQMCAR

CsaHc5b -------YPANWN------EELTDKV--KD----RKGELFYYMHQQMCAR

CsaHc6a -------YPANWN------EELTHKV--KD----RKGELFYYMHQQMCAR

CsaHc6b -------YPANWN------EELTHKV--KD----RKGELFYYMHQQMCAR

CsaHc6c -------YPANWN------EELTHKV--KD----RKGELFYYMHQQMCAR

HauHcB -------HPAIW-------TEELGRG--KE----RRGELFYWMHQQMIAR

HauHcA -------HPAIW-------TEELGRG--KE----RRGELFYWMHQQMIAR

ScoHcA -------HPAVW-------LPKHGPR--KD----RKGELFYYMHHQMVAR

ScoHcD -------HPAIW-------LPKHGGV--KD----RKGELFFYMHKQMVAR

ScoHcX NSALRKAYPTE-----------------GEKKFYRKGELFYHMHHQMLNR

ScoHcC -------HPAIW-------VSELGNE--KD----RKGEFFYWMHHQMLAR

ScoHcB -------HPAVWQESLEELTHQH-----KD----RKGELFYFMHHQMVNR

SpiHc1 -------HPSIW-------TQDIGE---KS----KLGELFYWMHRQMVAR

AgiHc1 -------HPSIW-------HQGVGE---KA----KLGELFYCMHRQMVAR

PanHc1 -------HPLFLP------DVIEGVH--KD----RIGELFYWMHRQMVAR

PanHc2 -------NPAVW-------KNKYGNY--SD----RKGELFYYMHSQMVAR

PciHc1 -------FPFWWH----------GEH--ID----RKGELFFWAHHQLTAR

PciHc2 -------FPFWWN----------GEH--ID----RKGELFFWAHHQLTAR

NkeHc -------FPFWWE------DKY-GHH--ID----RKGELFFWMHHQLTVR

GpuHc1 -------FPFWWE------DSY-GYH--LD----RKGELFFWAHHQLTVR

GroHc1 -------FPFWWE------DSY-GYH--LD----RKGELFFWAHHQLTVR

CcsHc -------FPFWWD------DSY-GYS--LD----RKGELFFWVHHQLTAR

EpuHc1 -------YPFWWK------DSY-GYH--LD----RKGELFFWAHHQLTVR

EpuHc2 -------YPFWWK------DSY-GYH--LD----RKGELFFWVHHQLTVR

OscHc1 -------FPFWWQ----------GYR--ID----RKGELFFWAHHQLTAR

OscHc3 -------FPFWWE----------GYR--ID----RKGELFFWAHHQLTAR

OscHc2 -------FPFWWH----------GEK--ID----RKGELFFWAHHQLTAR

OscHc4 -------FPFWWH----------GEK--ID----RKGELFFWAHHQLTAR

PleHc -------FPFWWN----------GAK--ID----RKGELFFWAHHQLTAR

PleHc2 -------FPFWWK------DSY-GYH--LD----RKGELFFWVHHQLTAR

HamHcA -------FPFWWK------DSY-GYH--LD----RKGELFFWAHHQLTVR

PinHcB -------FPTWWQ------DSY-GYH--LD----RKGELFFWVHHQLTAR

PinHcA -------FPFWWE------DSY-GYH--LD----RKGELFFWVHHQLTAR

PvuHc -------FPFWWD------DSY-GYH--LD----RKGELFFWVHHQLTAR

PelHc1 -------FPFWWD------DSY-GYH--LD----RKGELFFWVHHQLTAR

PelHc2 -------FPFWWQ------DSY-GYH--LD----RKGELFFWVHHQLTAR

PelHc3 -------FPFWWD------DSY-GYH--LD----RKGELFFWVHHQLTAR

PelHc4 -------FPFWWD------DSY-GYH--LD----RKGELFFWVHHQLTAR

CmaHc6 -------FPFWWD------DAHENHH--IE----RKGESFFWVHHQLTVR

CmaHc1 -------YPFWWH----------GQE--ID----RKGELFFWAHHQLTAR

CmaHc2 -------YPFWWH----------GQE--ID----RKGELFFWAHHQLTAR

CmaHc3 -------FPFWWD------DAHENHH--IE----RKGENFFWVHHQLTVR

CmaHc4 -------FPFWWD------DAHENHH--IE----RKGESFFWVHHQLTVR

CmaHc5 -------FPFWWD------DAHENHH--IE----RKGESFFWVHHQLTVR

CsaHc -------FPFWWD------DSHENHH--IN----RKGESFFWVHHQLTVR

PinHcC -------FPFWWE------DSS-GRH--LD----RKGESFFWVHHQLTVR

PvaHc1 -------FPFWWD------DKY-SHH--LD----RKGGNFFWVHHQLTVR

PvaHc -------FPFWWN------DAY-GHH--LD----RKGENFFWIHHQLTVR

FchHc1 -------FPFWWQ------DEY-SHH--LD----RKGESFFWVHHHLAVR

MjaHcL -------FPFWWQ------DKY-SHH--LD----RKGENFFWVHHQLTVR

MjaHcY -------FPFWWD------DKY-GHH--LD----RKGENFFWVHHQLTVR

CjaHc1 -------FPFWWK------DSY-GHK--LD----RKGENFYWVHNQLTHR

PmaHc2 -------FPFWWK------R-SYDVT--KD----RRGELFFYMHHQMVNR

StuHc2 -------FPFWWK------P-EYGIE--LD----RKGELFFYNHHQMTSR

PgrHc2 -------FPFWWK------R-SYDIT--KD----RRGELFFYMHHQMVNR

CseHc2 -------FPFWWK------Q-EYTVD--KD----RKGELFFYMHHQMVAR

PamHc2 -------FPFWWK------Q-EYAGY--KD----RKGELFYYMHHQMVAR

BduHc2 -------FPFWWK------P-EYDGD--KD----RKGELFFYMHHQAVAR

HmeHc2 -------FPFWWK------P-HYQHNHSKD----RKGELFFYMHHQMVAR

TdoHc2 -------FPFWWK------P-EYGVE--KD----RKGELFYYMHHQLIAR

StuHc3 -------FPFWWK------K-DYPTQ--MD----RKGELFWYAHHQLTTR

StuHc1 -------FPFWWK------K-AYGTE--KD----RKGELFFYSHHELTTR

PmaHc1 -------FPFWWK------AAEYGIE--KD----RKGELFYYMHHQMIAR

SamHc1 -------FPFWWK------EDEYGIR--KE----RKGELFYYMHHQLIAR

BduHc1 -------FPFWWK------EQEYGSH--KD----RKGELFYWMHHQLISR

PamHc1 -------FPFWWK------QEEYGVH--KD----RKGELFYYMHHQLIAR

CseHc1 -------FPFWWK------QDEYGVK--KD----RQGELFYYMHHQLIAR

HmeHc1 -------FPFWWK------QQEYGVE--KD----RKGELFYWMHHQLIAR

CmoHc1 -------FPFWWK------PEEYGID--KD----RKGELFYYMHHQLIAR

CacHc1 -------FPFWWK------EHEYGIH--KD----RKGELFYYMHHQLIAR

TdoHc1 -------FPFWWK------P-EYGVE--KD----RKGELFYYMHHQLIAR

PgrHc1 -------FPFWWK------AAEYGIE--KD----RKGELFYYMHHQMIAR

ScuHc1 -------WPFWWN------EEKYGLT--KD----RKGELFWYMHHQLITR

MgeHc1 -------FPFWWS------P-HYDTK--FD----RAGEMFWYMHHQLVAR

PlaPPO YDAERLSNYMARV--QPLSDLRAPIREGYASQLTSIVSGRHYSGRPAHMV

AfrPPO YDCERLSNGLPLV--RSFHKLDEPIEEAYFSKLTTDNSGKLWGVRPAGMK

DmaPPO YDAERIANGLARV--TSYHDWDEPIMEAYFPKLTNANGAIHWASRPAGLV

DmePPOA1 YNVERFCNNLKKV--QPLNNLRVEVPEGYFPKILSSTNNRTYPARVTNQK

DmePPO2 YNAERFSNNLARV--LPFNNLRDPIAEGYFPKMDSLVASRAWPPRFESTR

PseHc YNGERYAAGLKFVQPIPMTLSDITIEEGYFPNLFSSIIGSDFGGRPANSM

EpiHc1 YDAERLCNGMRRV--KSLHDYDEIIPEGYYPRLHSKISGTEYASRQANTR

EspHc1 YDQERLANGMQRM--KPFHNWDEPM-EGYAPHLTSLVDGQHYGVRPTGMK

PimHc2 YDCDRLSVGLPRM--TPFHNFDEPL-EGYAPHLTSLISGLGYASRPAGLA

PimHc3A YDCERLSNGLHRM--IPFHNFHEFL-EGYAPHLWHQSSGSYYAFRPEEMA

PimHc3B YDCERLSNGLKRT--VPFHNFDEEL-EGYAPHLAHFASGRYYSARPAGLK

PimHc3C YDCERLSNDLPRM--IPFHNFDEPL-DGYSPHLFSCINGLPYASRPSGLT

PimHc4 YDCERLSNGLNRM--VPFHNFDEKL-EGYAPHLTSLVSGLQYGNRPTGFS

PimHc5A YDCDRLSSGLKRM--TPFHNFHEKL-EGYSPHLTSLISGLNYASRPAGLS

PimHc5B YDCERLSNSLPRM--LPFHNFEDPL-EGYSSHLSSTINGQPYASRPAGVV

PimHc6 YDCERLSNDLQRM--TPFHNFEEPL-EGYAPHLTSLVSGLQYASRPEGYS

AauHc6 YDSERLSNGLQRM--IPFHNFDEPL-EGYAPHLTSLVSGLQYASRPEGYS

LpoHcII YDCERLSNGMHRM--LPFNNFDEPL-AGYAPHLTHVASGKYYSPRPDGLK

LpoHcIIIa YDCERLSNGMHRM--FPFNDFHEEL-EGYSPHLTSLISGLNYGTRPDGLK

LpoHcIIIb YDCERLSVGLQRM--LPFSNFEEEL-EGYSAHLTSLVSGLHYASRPEGMH

LpoHcIV YDCDRMSVGLQRM--IPFQNFEETL-EGYSAHLTSLVSGLHYASRPEGLH

LpoHcVI YDCERLSNGMPRM--EPFHNFHEPL-EGYSSHLSSSINGLPYPSRPDGMT

CroHcVI YDCERLSNGMPRM--EPFRNFHDTL-EGYSSHLSSSINGLPYPSRPEGMT

CroHcV YDCERLSNDMPRM--EPFHNFHSPL-EGYSSHLTSLINGLSYPSRPAGLK

CroHcIV YDCDRMSVGLQRM--IPFQNFEETL-EGYSAHLTSLISGLHYASRPEGLH

CroHcIIIb YDCERLSVGLQRM--LPFQNFEEEL-EGYSAHLTSLVSGLHYASRPEGMH

CroHcIIIa YDCERLSNGMHRM--YPFNNFHEDL-EGYSPHLTSLISGLNYGTRPDGLK

CroHcII YDCERLSNGMHRM--LPFHNFDEPL-AGYAPHLSHIASGKYYSPRPDGLS

CroHcI YDCERLSNHMHRM--SPFSNLHEEL-EGYAPHLTSMISGLHYGVRPSGMK

AgoHcA YDCERLSNGLNRM--IPFHNFNEPL-GGYAAHLTHVASGRHYAQRPDGLA

AgoHcB YDSERLSNGMPPM--VPFHNFHDPM-EGYSSHLSSGINGMPYAFRPHGRI

AgoHcC YDCERLSNGLPRM--IPFHNFHEPL-EGYNPHLSSTQNGLPYASRPEGMT

AgoHcD YDCERLSVGLQRM--IPFQNLDDAL-EGYSPHLSSLVSGLSYGSRPSGMH

AgoHcE YDCERLSNGMRRM--IPFSNFDEEL-EGYSAHLTSLVSGLPYAFRPAGLC

AgoHcF YDCDRLSTGLRRM--IPFHNFDEKL-EGYSPHLTSLVSGLNYASRPAGLH

AgoHcG YDCERLSNGLTRM--IPFHNFKEKL-EGYAPHLTSLVSGLHYASRPAGLC

AgoHcX YDCERLSVGLQLV--DAMYNFDEPM-IGYSPHLMVLETGKSSASRPDGMR

EcaHcA YDCERLSNGLNRM--IPFHNFNEPL-GGYAAHLTHVASGRHYAQRPDGLA

NinHcA YDCERLSNGLTRM--VPFHNFEEPL-EGYAAHLTHIASGRHYAPRPDGLA

EcaHcB YDSERLSNGMAPM--VPFHNFHEPM-EGYSSHLSSGINGMPYAFRPHGRI

NinHcB YDCERLSNHMPQM--IPFHNFHEPL-EGYSAHLSSNINGLPYSSRNAGRK

EcaHcC YDCERLSNGLARM--IPFHNFHEPL-EGYNPHLSSTQNGLPYAFRPEPMT

EcaHcD YDCERLSVGLQRM--IPFQNLDDEL-EGYSPHLRSLVSGLSYGSRPAGMH

NinHcD YDCDRLSVGLQRM--IPFQNFDDKL-EGYSAHLISLISGLNYASRPAGMT

EcaHcE YDCERLSNGMRRM--IPFSNFDEKL-EGYSAHLTSLVSGLPYAFRPDGLC

NinHcE YDCERLSNGMRRM--IPFHNFEEEL-EGYSAHLTSLVSGLQYASRPEGFR

EcaHcF YDCDRLSTGLRRM--IPFHNFDEKL-EGYSPHLTSLVSGLNYASRPAGLH

NinHcF YDCDRLSTGLQRM--IPYHNFNEKL-EGYSPHLTSLVSGHHYANRPAGLS

EcaHcG YDCERLSNGLTRM--IPFHNFKEKL-EGYAPHLTSLVSGLHYASRPAGLC

NinHcG YDSERLSNGLNRM--VAFHNFEEKL-EGYAPHLTSLVSGLHYASRPQGFS

TtrHcA YDCERLSNHMHRM--SPFSNLHEEL-EGYAPHLTSMISGLHYGVRPSGMK

EbaHcA YDCERLSNGLNRM--IPFHNFEEPL-EGYAPHLAHLASGRHYAPRPDHLR

MgiHcA YDCERLSNGLQRM--VPFHNFDEEL-EGYAPHLTHIGSGRYYSSRPDHMT

EbaHcB YDCERLSNNLPKM--RPFHNFHEPM-EGYSAHLIAIINGQPYATRTEGMT

MgiHcB YDCERLSNGLPRM--IPFHNFEEPM-EGYSSHLVASINFMPYAFRTAGST

EbaHcC YDCERLSNGMPRV--IPFHNFTEKL-HGFSSHLSSGISGKHYSSRAEGLI

MgiHcC YDCERLSNDLPRM--VPFHNFHEPL-EGYSAHLSSIINGLPYSSRSKGLA

EbaHcD YDCERLSTGLQRM--KPFHNFDEEL-EGYSAHLTSLVSGLNYGNRPEGLT

MgiHcD YDCERLCTGLQRM--APFQNFEEEL-EGYAPHLVSLVSGAPYGTRSEGMH

EbaHcE YDCERLSIGLQRM--IPFHNFEEPL-EGYAPHLTSLVSGLNYASRPEGFS

MgiHcE YDCERLSCGMQRM--TPFHNFHEEL-EGYSPHLTSLISGLHYASRPEGFS

EbaHcF YDCERLSTELRRM--IPFHDFHQKL-EGYAPHLTSLISGLNYASRPTGLS

MgiHcF YDCDRLSTGLRRL--IPFHNFHEEL-EGYNPHLTSLVSGQNYASRPAGLS

EbaHcG YDCERLSNGMNRM--IPFHNFEEHL-EGYAPHLTSLISGLNYGSRPEGFS

MgiHcG YDCERLSNGMNRM--IPFHNFEEKL-EGYAPHLISLVSGLQYGARPQGFS

CsaHc1 YDCERLCNGLNRM--VPFHNFEEKL-EGYAPHLTSLISGLHYASRPEGFS

CsaHc2 YDCERLSNGLNRM--VPFHNFEEEL-EGYAPHLTSLVSGLHYASRPQGLR

CsaHc3 YDCERLSNGLNRM--IPFHNFEEKL-EGYAPHLTSLVSGLHYASRPTGFS

CsaHc4 YDCERLSNGLNRM--VPFHNFEEHL-EGYAPHLTSLVSGLHYASRPEGVS

CsaHc5a YDCERLSNGLNRM--IPFHNFEEKL-EGYAPHLTSLVSGLHYASRPQGFS

CsaHc5b YDCERLSNGLNRM--IPFHNFEEKL-EGYAPHLTSLVSGLHYASRPQGFS

CsaHc6a YDCERLSNGLNRM--IPFHNFEEKL-EGYAPHLTSLVSGLHYASRPTGFS

CsaHc6b YDCERLSNGLNRM--IPFHNFKEKL-EGYAPHLTSLVSGLHYASRPTGFS

CsaHc6c YDCERLSNGLNRM--IPFHNFKEKL-EGYAPHLTSLVSGLHYASRPTGFS

HauHcB YDAERLSNDMAPT--SVFENFDEPIEEGYASDLTVEHTGYRYMFRPEGLT

HauHcA YDAERLSNDMAPT--SVFENFDEPIEEGYASDLTVEHTGYRYMFRPEGLT

ScoHcA YDSERLSNNLPRT--EPFENWDDPLEEGYAPHLTIHKTGYNYMFRPEGLI

ScoHcD YDTERLSNDLPRV--RPFENWNDPIDEGYSPHLIIDKTGYKYAYRPQGVI

ScoHcX YELERLSNGLPRC--PTFENWDDPIAEGYASHLAVDRTGYRYTFRPDNLH

ScoHcC YEAERMSNGLART--RTFQNWNDPIDEGYAPHISIMKTGYTYAYRPPGYT

ScoHcB YDAERLSNGLPRS--TTFENWNDPIETGYAPHLTIDRTGYRYQFRPDNLV

SpiHc1 FDSELLSVHLPRI--SSLDDWNKKVKIGYAPHLTIQRTGYTYMNRPENLE

AgiHc1 FDSELLSVHLPRI--PSLDDWNKKVKIGYAPHLTIQRTGYTYMNRPENLE

PanHc1 FDSELLSNHLPRV--SAFEHWDEPIHTGYAPHLTIHRTGYRYLYRPDGLI

PanHc2 YDIERLSNGIPRT--VPFQSFNDEIEEGYNPHLTMAKTQYHYAYRPPHFT

PciHc1 FDAERLSNWLSPV--DELH-WDRPIKDGFAPH-TSYKFGGEFPTRPDNKA

PciHc2 FDAERLSNYLSPV--DELH-WDRPIKDGFAPH-TSYKFGGEFPTRPDNKA

NkeHc FDAERLSNNLDIV--DELY-WDKPIYRGFAPH-TTYRHGGEFPSRPDNID

GpuHc1 FDAERLSNHLDLV--DELY-WDRPIKEGFAPH-TTYHYGGEFPTRPDNVH

GroHc1 FDSERLSNHLDLV--DELY-WDRPIEDGFAPH-TTYRYGGEFPTRPDHVN

CcsHc FDAERLSNGLDLV--DELY-WDKPIVEGFAPH-TTYRYGGEFPARPDNVN

EpuHc1 FDAERLSNHLDMV--DELY-WDKPIYEGFAPH-TTYKFGGEFPSRPDNVR

EpuHc2 FDAERLSNHLDMV--DELY-WDKPIYEGFAPH-TTYKFGGEFPSRPDNVR

OscHc1 FDAERLSNYLPVV--DELY-WSRPIEEGFAPH-TTYRYGGEFPSRPDNKA

OscHc3 FDAERLSNYLPVV--DELY-WSRPIEEGFAPH-TTYRYGGEFPSRPDNKA

OscHc2 FDAERLSNYLPVV--DELY-WSRPIKEGFAPH-TTYRYGGEFPSRPDNKI

OscHc4 FDAERLSNYLPVV--DELY-WSRPIKEGFAPH-TTYRYGGEFPSRPDNKI

PleHc YDAERLSNFLPAV--DELY-WDRPIKDGFAPH-TTYKYGGEFPARPDNKE

PleHc2 FDSERLSNWLDVV--DELH-WEDVIHEGFAPH-TSYKFGGEFPARPDNVH

HamHcA FDAERLSNWLDPV--DELH-WERIIHEGFAPH-TSYKYGGEFPARPDNVH

PinHcB FDFERLSNWLDPV--DELH-WDRIIREGFAPL-TSYKYGGEFPVRPDNIH

PinHcA FDFERLSNWLDPV--DELH-WDRIIREGFAPL-TSYKYGGEFPVRPDNIH

PvuHc FDAERFSNWMDPV--DELH-WDDIIHEGFAPH-ASYKYGGEFPTRPDNTH

PelHc1 FDAERLSNWMEPV--DELH-WDDIIHEGFAPH-TSYKYGGEFPVRPDNIR

PelHc2 FDAERLSNWMDPV--DELH-WEEVIHEGFAPH-ASYKYGGEFPTRPDNTQ

PelHc3 FDAERLSNWLDPV--AELH-WVDVIHEGFAPH-ASYKHGGEFPTRPDNIR

PelHc4 FDAERLSNWMEPV--DELH-WDDIIHEGFAPH-TSYKYGGEFPVRPDNIR

CmaHc6 FDAERLSNYLDPV--DELH-WDDVIHEGFVPH-TMYKYGGYFPSRPDNVH

CmaHc1 FDAERLSNHLPLV--DELY-WDRPIKEGFAPH-TSYKYGGEFPTRPDNKN

CmaHc2 FDAERLSNHLSPV--DELY-WDRPIKEGFAPH-TSYKYGGEFPTRPDNKN

CmaHc3 FDAERLSNHLDPV--DELH-WDDVIHEGFDPQ-AMYKYGGYFPSRPDNIH

CmaHc4 FDAERLSNYLDPV--DELH-WDDVIHEGFAPH-TMYKYGGYFPSRPDNVH

CmaHc5 FDAERLSNHMDPV--GELH-WDDVIHEGFAPH-TMYKYGGYFPSRPDNVH

CsaHc FDAERLSNYLDPV--DELH-WDDMIHEGFAPH-TMYKYGGYFPSRPDNVN

PinHcC YDAERLSNHLDPV--EELS-WNKAIDEGFAPH-TAYKYGGYFPSRPDNVH

PvaHc1 FDAERLSNYLDPV--EELS-WDKPIVQGFAPH-TTYKYGGQFPSRPDNVD

PvaHc FDAERLSNYLDPV--GELQ-WNKPIVDGFAPH-TTYKYGGQFPARPDNVK

FchHc1 FDAERLSNYLDPV--DELH-WEKPIVQGFAPH-TTYKYGGQFPSRPDNVN

MjaHcL FDAERLSNYLDPV--DELH-WEKPIVQGFAPH-TTYKYGGQFPSRPDNAR

MjaHcY FDAERLSNYLDPV--GELQ-WHKEIVEGFAPH-TTYKYGGQFPTRPDNVN

CjaHc1 FDAERISNYLEPA--EELD-WHKSIEEGFAPH-TTYKYGGQFPARPDHLE

PmaHc2 FDAERLSNWLPQV--EPLN-WHHEIEEGFAPA-AMYFNGQEFPMRPDGIH

StuHc2 FDLERLSNDLTIS--KPLA-WYRPVVEGFSPD-AIYKHGFQFPMRPDNTK

PgrHc2 FDAERLSNFLPQV--EPLD-WHHEIEEGFAPA-AMYFNGQEFPMRPDGMH

CseHc2 FDAERLSNDLPAV--EPLE-WDQQITEGFAPA-ATYENGQEFPMRPDNMK

PamHc2 FDAERLSNDLPVV--EPLE-WDQKIVEGFAPG-ATYENGQEFPMRPDNMC

BduHc2 FDAERLSNDLPVV--QPLE-WDKKIVEGFAPG-ASYENGQEFPMRPDNMQ

HmeHc2 FDAERLSNNLPVV--QPLE-WNRKIVEGFAPG-AMYDNGQEFPMRPDNIG

TdoHc2 FDAERLSNYLPVV--EPLD-WEKPIVQGFAPG-AMYENGQEFPVRPDNMN

StuHc3 FDLERLSNNLDVV--KPLA-WDKPIEHGFSPE-TVYRTGDEFPVRPDDMM

StuHc1 YDLQRLSNNLPIV--GPLA-WDKPIHNGFYPQ-AAYRNGHEFPARPDDVK

PmaHc1 YDFERLSNWLHFV--EPIS-FEDEIEHGFYPQ-TTYRVGGEFPARPDNFH

SamHc1 FDLERLSNDLPFV--EPLY-WTERIKDGFYPQ-TTYRVGGEFPARPDNFA

BduHc1 FDLERLSNDLPFV--EPLY-WDEKIVDGFYPQ-TTYRVGGEFPARPDNFE

PamHc1 FDAERLSNDLPIV--EPLY-WDEKIVDGFYPQ-TTYRVGGEFPARPDNFE

CseHc1 LDLERLSNDLPFV--KPLY-WEDKIEDGFYPQ-TTYRVGGEFPARPDNFE

HmeHc1 FDLERLSNDLPFV--EPLS-WEDKIVDGFYPQ-TTYRVGGEFPARPDNFE

CmoHc1 FDLERLSNDLPPV--EPLG-WRERIVDGFYPQ-TTYRVGGEFPARPDDFE

CacHc1 FDLERLSNNLPFV--EPLS-WDQKIANGFYPQ-TTYRVGGEFPARPDNYA

TdoHc1 FDLERLSNNLPVV--RPLD-WEKPIVNGFYPQ-TTYRKGGEFPARPDNFY

PgrHc1 YDLERLSNWLPFV--EPLS-FEEKIEHGFYPQ-TTYRVGGEFPARPDNFH

ScuHc1 FDAERLSNDLNEV--EALH-WDKPIVEGFYPQ-TTYRKGGEFPARPDNFM

MgeHc1 YDLERLSNYLPEV--EPLE-WFKPIKSGFAPL-TMYRKGGEFPNRPDNML

PlaPPO LSDTID----------VSLQRIEQAKSRIQEAIHRGYAIDRQ--AKQVPL

AfrPPO IQDMELPEPNEN----YRIMDMEGWRDRIRDAIHRGIARRTD--GTEVRL

DmaPPO LKDINIPDEGVELGIEFQINNLELWRTRILHAIHKGSVAKAN--GEIIYL

DmePPOA1 LRDVDRHDGR------VEISDVERWRDRVLAAIDQGYVEDSS--GNRIPL

DmePPO2 LSDLNRESDQLN----VEIGDLERWRDRIYEAIHQGFVMDER--GNRVPL

PseHc LQNITNHPIA----GDSNIADLLYWYGNVLTAINAGQVTLPD--GGTLPL

EpiHc1 MMDTLD----------NTVIELERFRERIEHAISVGYIELPD--NTKTPL

EspHc1 MSDVKD----------VDIQDMMRWRDRFMDAVNLGYVIDRK--GQRVDL

PimHc2 MRDIAD----------VDVQDMERWRDRILDSIHIGTVIDDD--GNEIPL

PimHc3A MSDMKD----------VDVQDMERWRERILDAIDIGHAINHD--GKEILL

PimHc3B LSDMKD----------VDVQDMERWRERILDAIDLGLIIDEG--GKEIPL

PimHc3C LRDMKE----------VSVLDLERWRDRILSAIHLGHVTKET--GEEITL

PimHc4 LRDLKV----------VDVQEMERWRERILDAIHLGHIHASD--GTEIAL

PimHc5A MHDITD----------IDVQDMIRWRERVLDAIHVGHVQDEH--GKEIPL

PimHc5B LRDLKD----------VSVQELARWRERILDAIHLESVIDEH--GKDTPI

PimHc6 IKDLSD----------VDVQEMERWRERILDAINIHHIVDSH--NKKIPL

AauHc6 IHDLSD----------VDVQDMVRWRERILDAINMHYIVDKD--NNKIPL

LpoHcII LRDLGD----------IEISEMVRMRERILDSIHLGYVISED--GSHKTL

LpoHcIIIa LHDLHD----------VTIQDMERWRERIHDAIDLKMVHDHH--GKEVAV

LpoHcIIIb LHDLKGV---------VNLHDMERWRERIMDAIERGLVYDEH--GHEHKL

LpoHcIV IRDLND----------VSLQEMERWRERILEGINLGHVHDDH--GKEVEL

LpoHcVI LQDLKE----------VSVQDLERWRDRISDAIHIGHVEDEN--GSHVPL

CroHcVI LQDLKE----------VSIQDLERWRDRISDAIHIGHVEDEN--GSHVPL

CroHcV LQDLKD----------VSIQDMERWRSRISDAIHIGHVQDEN--NHEYLL

CroHcIV IRDLND----------ISLQEMERWRGRILDAIHPGHLHDAH--GNEVKL

CroHcIIIb LHDLKGV---------VNLYEMERWRERIMDAIERGLVFDDD--NHEHKL

CroHcIIIa LHDLHD----------VTIQDRERWRERIHNAIDLKMVHDHD--GKEIAI

CroHcII LRDLGD----------IAISDMVRMRERISDSIHLGYVIDSE--GNHKNL

CroHcI LHDIDV----------VTIQDMERWVQRIMEAIDIHMVNDTH--GNKLKL

AgoHcA MHDMHE----------VDVQDMERWTERIMEAIDLRRVISPG--GEYAAL

AgoHcB LKDMRE----------VSVQDLERSRERILDAINLRYVVDPN--GLETPL

AgoHcC LCDMHD----------VSVQDLERWRERILDAINLGEVTDPH--GDEFAL

AgoHcD LRDIND----------CSVQDMERWRERILDAIHTGHVTDSH--GKEIKL

AgoHcE LHDLKD----------IDLKEMFRWRERLLDAIDSGYYIDGQ--GHEVKL

AgoHcF LRDLVDF---------VDVQDMARWRERLLYSIDIGHVFDHE--GQEIPL

AgoHcG LRDLSE----------LEVQDVERWRDRILDAIHLHHVHDRE--NHDVVL

AgoHcX M---------------TEINHLVRWRERIMEAIHQKFLVGRG--VNHIPL

EcaHcA MHDVRE----------VDVQDMERWTERIMEAIDLRRVISPT--GEYIPL

NinHcA MTDLRL----------VDVQDMQRWTERILEAIHLGKVIDSE--GNDIIL

EcaHcB LKDMRE----------VSVQDLERSRERLLDAINLGYVVDPN--GLETPL

NinHcB LADLEE----------CAVQDMERWRERILQSINLGVVVDPN--GHETVL

EcaHcC LCDMHD----------VSVQDLERWRERILDAINLGEVTDPN--GDEYAL

EcaHcD LRDIND----------CSVQDMERWRERILDAIHTGLVTDSH--GKEIKL

NinHcD LRDIRE----------VDVQDMERWRERILSAIHTGQVIDSN--GQEVPL

EcaHcE LHDLKD----------IDLKEMFRWRERILDAIDSGYYIDNE--GHQVKL

NinHcE LHDLKD----------VDVQDMIRWRERILEAIDLGFVEDES--HQQIKL

EcaHcF LRDLVDF---------VDVQDMARWRERLLYSIDIGHVIDHE--GQEIPL

NinHcF LHDLNEL---------VDVQDMARWRERLLEAIHLGVVIDDH--GNEIVL

EcaHcG LRDLSE----------LEVQDVERWRDRILDAYHLNHVHDRE--NNDVVL

NinHcG LRDLTE----------VDVQDMERWRERILEAIDLKHVHDSK--GNEVPL

TtrHcA LHDIDV----------VTIQDMERWVQRIMEAIDLHLVNDTH--GN--KL

EbaHcA LHDVKG----------VDVLDMMRWRERILEAIHLGHVTDSH--GNDIPL

MgiHcA LHDLSE----------VDVLDMIRWRERIMQAINLRHVIDEH--NKEIPL

EbaHcB LRDLDE----------VAVQDLERWRDRILDAIHLGFVEDEN--GVHIPL

MgiHcB LQDTPE----------CAVQDLERWRDRIIGAIQLGHVVDET--GTEFPL

EbaHcC LQDLSD----------VTVQDMERWRDRILDAINLGFVTDEH--GRETVL

MgiHcC LHDMHD----------ISVQELERFRERISGAINLGYVTDTH--GNEIDL

EbaHcD MKDVPQ----------VGVQDMIRWRERILDAIHTGHVHDVN--NKEIPL

MgiHcD MHDVND----------VDVLDLIRWRERILNAIHTGHVTDYN--GKEVKL

EbaHcE LRDLKD----------VDVQEMIRWRERILEGIHLGYVIDST--GTHIPL

MgiHcE LEDTED----------VSLVEMIRWRERILTAIHLGHIIDDE--GKDVKL

EbaHcF MSDLTE----------VDVQDMERWRERILDAIHGGFVYDKE--GNEVPL

MgiHcF MKDIHE----------CDVQDMIRWRERILDAIHIGFLYDDH--GNEIPL

EbaHcG LRDLKD----------VDVQDMERWRERILESIHLGYVHGHG--EERIPL

MgiHcG LRDLKD----------VDVQSMERWRERIIEAIQLGYVIDEH--GEHVNL

CsaHc1 LRDLVD----------VDVQEMERWRERILKAIDLHFVHGAN--GQTLRL

CsaHc2 LCDLPD----------VNVQNMERWRERILEAIDLQHVHAAD--GSELAL

CsaHc3 LRDLPD----------VDVQDMERWRERILEAIDLQHVHAAD--GSELAL

CsaHc4 LQDLPD----------VDVQDMERWRERILEAIDLQHVHAAD--GSEIAL

CsaHc5a LRDLPD----------VNVQNMERWRERILEAIDLQHVHAAN--GSEIAL

CsaHc5b LRDLPD----------VNVQNMERWRERILEAIDLQHVHAAN--GSEIAL

CsaHc6a LRDLPD----------VDVQDMERWRERILEAIDLQHVHAAD--GTELAL

CsaHc6b LRDLPD----------VDVQDMERWRERILEAIDLQHVHAAD--GTELAL

CsaHc6c LRDLPD----------VDVQDMERWRERILEAIDLQHVHAAD--GTELAL

HauHcB LHDLPE----------LTKNQLRLWRIRILNAINKGYAYDKE--GHKVPL

HauHcA LHDLPE----------LTKNQLRLWRIRILNAINKGYAYDKE--GHKVPL

ScoHcA VRDLPE----------LNKNKMRQWKSRILHGIHLNVLYAEN--GTKISL

ScoHcD VHDLPN----------LPKTKMFEWKNRIMVGIRKGSLISAN--KTQVPL

ScoHcX VRDLPE----------LTKDNMRVWRDRIFDAATSCSVLREN--GSFVKI

ScoHcC LRDLPN----------LPKNKMVEWAKRVLYSIHSGIFHFSN--GTDAHL

ScoHcB VRDLPE----------LTKNHMRQWRDRILYAVHRGEALAAN--GSSVSL

SpiHc1 IEDLPE----------LTKGELMQWKNRIMEAIARHNITLKGGPGKKLRT

AgiHc1 IEDLPE----------LTKGELMQWKNRIMEAIARHNITLKGGPGKKLRV

PanHc1 LQDLPE----------LTKSQMQQWKLRILKAIKRSNYVATN--GSYVAL

PanHc2 LQDLPT----------LPKSRLQEWSNRLFHAIHTGQLTRPN--GRKLAL

PciHc1 FDDVDGV---------ARMSDMIITESRIRDAIAHGYIEKED--GTHVNI

PciHc2 FDDVDGV---------ARMRDMIITESRIRDAIAHGYIEKED--GTHVNI

NkeHc FEDVDGV---------ARVRDMKIMENRIRDAIAHGYIENDH--DEHISL

GpuHc1 FEDVDGL---------VRVRDMIIHESRIRDAIAHGYITAAD--GSHINI

GroHc1 FEDVDGL---------VRVRDMIIHETRIRDAIAHGYITAAD--GSHINI

CcsHc FEDVDGA---------VRVRDMIVHETRIRDAIAHGYITSED--GSRVDI

EpuHc1 FEDVDGV---------ARIRDMIIYESRIRDAIAHGYVTAKD--GSHIDI

EpuHc2 FEDVDGV---------ARIRDMIIYESRIRDAIAHGYVTAKD--GSHIDI

OscHc1 FEDVEGV---------ISVQEMLDTESRIRDAIAHGYVTSTD--GKAIYI

OscHc3 FEDVEGI---------VKVHEMLDTESRIRDAIHHGYVTSKD--GKAIYI

OscHc2 FEAVEGV---------ARIRDMKEMESRIRDAISHGYITAKD--GHPIYI

OscHc4 FEAVEGV---------ARIRDMKEMESRIRDAISHGYFTSHS--GDPIYI

PleHc FEDVDGV---------ARIRDLKELESRIRDAVAHGYIINAD--GTKTDI

PleHc2 FEDVDGV---------ARVRDMVILESRIRDAIAHGYIIDHS--GNKIDI

HamHcA FEDVDGV---------AHVRDMIIIESRIRDAIAHGYVTDNH--GDNINI

PinHcB FEDVDGV---------AHVHDLEITESRIHDAIDHGYITDSD--GHTIDI

PinHcA FEDVDGV---------AHVHDLEITESRIHEAIDHGYITDSD--GHTIDI

PvuHc FKNVDGV---------ARVRDMEITENRIRDAIAHGYITATD--GHTIDI

PelHc1 FEDVDGV---------AHVHDMEITENRIRDAIAHGYITATD--GHTIDI

PelHc2 FKNVDGV---------ARVRDMEITESRIRDAIAHGYITATD--GHTIDI

PelHc3 FKNVDGV---------ARVHDMEITESRIRDAIAHGYITATD--GHTIDI

PelHc4 FENVDGV---------AHVHDMEITENRIRDAIAHGYITATD--GHTIDI

CmaHc6 FEDVDGV---------ARVRDMLILESRIRDAIAHGYVTGRD--GSIISI

CmaHc1 FEDVDGV---------ARIRDLKEMENRIRDAIAHGYADNTD--GTHLDI

CmaHc2 FEDVNGV---------ARIRDMKIMESRIRDAIAHGYADNTD--GTHLDI

CmaHc3 FEDVDGV---------ADVRDMLLYEERILDAIAHGYVRDRN--GKIVDL

CmaHc4 FEDVDGV---------ARVRDMLILESRIRDAIAHGYVTGRD--GSIISI

CmaHc5 FEDVDGV---------ARVRDMLILESRIRDAIAHGYVTGRD--GSIISI

CsaHc FEDVDGV---------ARVRDMLILESRIRDAIAHGYFTGRD--GSVISI

PinHcC FSDVDGV---------ARVRDMSMTEDRIRDAIAHGYIDALD--GHSIDI

PvaHc1 FDDMDGV---------ARIRDLLIIESRIRDAIAHGYIVDKV--GNHIDI

PvaHc FEDVDDV---------ARIRDMVIVESRIRDAIAHGYIVDSE--GKHIDI

FchHc1 FEDVDGV---------ARIRDLLIVESRIRDAIAHGYIIDKQ--GNRIDI

MjaHcL FEDVDGV---------ARIRDLLIVESRIRDAIAHGYIVDRE--GKHIDI

MjaHcY FEDVDGV---------ARIRDMTIIESRIRDAIAHGYIVDSH--GKHIDI

CjaHc1 FEDVDGV---------ARIRDLVIIDSRIRDAIAHGYVVKED--GTHIDI

PmaHc2 FHDLPW----------FTVKDTEDYEDRIRNVIAKGYVKASD--GHTIFL

StuHc2 FHDLST----------VTVNHMRAYESRIHESIDFGHVYSTN--GTEVSL

PgrHc2 FHDLPW----------FTVKDTEEYEDRIRDIIAKGYVKAKD--GHVVFL

CseHc2 FCDLPS----------LSVRDMKIVEGRIRDAIASSFVKTAD--SRLLSI

PamHc2 FSDLPF----------LTVQDMKIFEGRIRDAIASGYVKTVD--SRLMSL

BduHc2 FSDLPW----------LTVQDMRLYEGRIRDAIASGFVRTAE--GKLVSL

HmeHc2 FQDLPW----------LTVQDMLMYEGRIRDAITAGFVRAYD--GNYVSL

TdoHc2 FHDLPW----------LTIEDMKGYEDRIRDAIASGFVISVD--DSLVYL

StuHc3 FHDIHGI---------VSVTDMKAMEYRILHTIDANLYVAEN--GTFPKL

StuHc1 FTDLDKDGFH------IKVSQMKEYERRIRDAIAMKAVYGKD--GHAISL

PmaHc1 FHDLEH----------IKIKDMLDYTRRIKEAISKQKVRSKN--GEKIPL

SamHc1 FHDLQN----------IKVQQMIDYTRRVRECISQQAVITET--GDLYNI

BduHc1 FQDLKN----------IKVKDMVDYTRRIREAISQQAVITKN--GDYYSL

PamHc1 FHDLEH----------IKVKDMMDYTRRIREAISQQAVVTRT--GEMVSL

CseHc1 FQDLQD----------IKVKDMVDYTRRIREAISQQSVITRW--GQNLPL

HmeHc1 FQDLEH----------IKVKDMVDYTRRIREAISQMAVVTKS--GEYYSL

CmoHc1 FQDLSS----------IKVQDMVDYERRVREAISQQAVITMS--GDYFSL

CacHc1 FHDLEN----------IKIKDMIDYTRRIREAIAQQAVITKS--GEYFYL

TdoHc1 FQDLETPY--------IKVKHMIAYSQRIREAIARGVVLTKS--GELYSI

PgrHc1 FHDLED----------IKIKDMLDYTRRIKEAIVKHTVINKN--GEHIPL

ScuHc1 FHDLKD----------HRVADLEAYEERIREAISAGVIFDAHDPNTFISL

MgeHc1 LHDLHD----------LKVHDVVVFEERIKEAIDRGFVYNKW-IGEKIRL

PlaPPO -RDP--KGIDTLGDMVE-ATVLSVHREYYGDWHNHGHRIISRIHDPDTRY

AfrPPO -DAK--TGIDILGDMIEPALSFSVNPRFYGQLHNKGHVLIGHCHDPTGAN

DmaPPO -DET--TGTNTLGELIE-ASQWSADKLFYGDLHNFGHIAVCYIHDPDRSH

DmePPOA1 -DEV--RGIDILGNMIEASPVLSINYNFYGNLHNEGHNIISFAHDPDYRH

DmePPO2 -DEA--TGIDTLGNMIE-SSILSPNRVLYGDLHNNGHTFISYAHDPTSKH

PseHc -TEE--KGIDILGSAVE-ASAISPNPDLYGDIHNTGHVLLSFIHDPTGVF

EpiHc1 NNE---QGIDILADLIE-ASNYSLNGAYYGDLHNTGHVMLATAHDPDKRF

EspHc1 -TTE--TGIDVLGALVE-SSYDSINAAYYGSLHNWGHVIMASAHDPDGRY

PimHc2 -DAE--HGVDLLGAIIE-SSEDSKNVDYYGSLHNWGHVMMARLHDPDGRY

PimHc3A -DEE--HGTDILGSIIE-SNAETINLPFYGSLHNWGHVMMAYIHDADGRF

PimHc3B -DEE--HGADILGDLIE-SSYESKNRIFYGNLHNMGHVMIARIHDPDGRF

PimHc3C -TET--FGIDVLGDIVE-SSHDSVNKNFYGSLHNWGHVMMAAIQDPKGKY

PimHc4 -DEQ--HGADLLGDIIE-SSAESRNPEFYGSLHNWGHVLMARIHDPDGRF

PimHc5A -DAE--HGIDILGALLE-SSYDSTNRGYYGTLHNWGHVILARVHDPDGRH

PimHc5B -DEE--HGINVLGNIIE-SNHDSVNEDYYGSLHNWGHVLIAEAHDHDGRY

PimHc6 -DVE--HGTDILGDIIE-SSYESKNPHFYGSLHNWGHVMMANITDPDHRF

AauHc6 -DIE--HGTDILGDIIE-SSDESKNVEYYGSLHNWGHVMMANITDPDHRF

LpoHcII -DEL--HGTDILGALVE-SSYESVNHEYYGNLHNWGHVTMARIHDPDGRF

LpoHcIIIa -DDE--HGIDILGALVE-SSHESVNQGFYGSLHNWGHVLTARAHDPEGKF

LpoHcIIIb -EEP--HGIDILGAMIE-SSHDSLNHEFYGSIHNWGHVMFARLHDPDGRY

LpoHcIV -DEE--HGIDILGALIE-SSHESKNEEYYGSLHNWGHVMLARVHDHDGRF

LpoHcVI -DDT--RGIDILGAMVE-ASYESINQAFYGSIHNWGHVISARVLDPDGRY

CroHcVI -DEA--RGIDVLGALVE-ASYESINQAFYGSIHNWGHVITARVQDPDGRY

CroHcV -DET--HGIDILGAIIE-ASHESINQHFYGSIHNWGHVITARVLDPDGRF

CroHcIV -DEE--HGIDTLGSLIE-ASHESKNEEYYGNLHNWGHVMLARVHDHDGRF

CroHcIIIb -EEP--HGIDMLGAIIE-ASHDSVKHDFYGSIHNWGHVMFARLHDPDGRY

CroHcIIIa -HEE--NGIDILGALVE-SSYESVNQGFYGSLHNWGHVLTARAHDPEGKF

CroHcII -DEH--HGIDILGALVE-SSHESVNKEYYGNLHNWGHVTMARIHDPDGRF

CroHcI -DET--TGIDVLGALVE-ASHDSINPEYYGSLHNWGHVITARAHDPEGKF

AgoHcA -DEE--HGCDILGAAIE-CSYESKNRGYYGSLHNWGHVMMAYIHDPDGRF

AgoHcB -DEI--HGIDILGAIIE-SSNDSVNKSYYGSLHNWGHVIMSSVDDPDGRY

AgoHcC -DET--FGIDALGAIIE-SSRDSKNREYYGSLHNWGHVLMANIVDPDGKY

AgoHcD -TEE--NGLNILGALIE-SSHDSVNKPFFGTLHNWGHVMIARIHDADGRY

AgoHcE -GTD--DGINVLGALIE-SSFETKNKLYYGSLHNWGHVMMARLQDPDHRF

AgoHcF -GPE--HGIDVLGALLE-SSHDSLNVDYYGNLHNSGHVMMARIHDPDGRF

AgoHcG -DAE--HGADILGAIIE-ASSNSVNRRFYGSLHNWGHVMMARMTDPDRSF

AgoHcX -DAE--NGINLLGALVE-ASADSIN-EYYGNLHNMLHNTIASVHDPKGKF

EcaHcA -DEE--HGADILGALIE-SSYESKNRGYYGSLHNWGHVMMAYIHDPDGRF

NinHcA -DEE--TGADILGSLIE-SNLESKNRQFYGNLHNWGHVMMAYIHDPDGRF

EcaHcB -DEL--HGIDILGAIVE-SSNDSVNKSYYGSLHNWGHVIMSAVDDPDGRY

NinHcB -DEI--HGIDILGNIIE-SSYDSVNVPFYGSLHNWGHVLMAAAHDPDGRY

EcaHcC -DET--FGIDVLGAIIE-SSRDSKNRESYGSLHNWGHVLMANIVDPDGKY

EcaHcD -TEE--NGLNILGALIE-SSHDSVNKPFYGTLHNWGHVMIARIHDADGRY

NinHcD -DLE--RGLDILGALIE-SSQESLNKGYYGTLHNWGHVMVAKIHDHDGRF

EcaHcE -DIV--DGINVLGALIE-SSFETKNKLYYGSLHNWGHVMMARLQDPDHRF

NinHcE -TED--NGIDILGSIVE-SSYESKNKLFYGSLHNWGHVMMANITDPDGRF

EcaHcF -DAE--HGIDVLGALLE-SSHDSLNDDYYGNLHNSGHVMMARIHDPDGRF

NinHcF -TPE--HGIDMLGSMLE-SSYESKNREYYGNLHNSGHVMMARIHDPDGRY

EcaHcG -DAE--HGADILGAIIE-SSSDSVNRRFYGSLHNWGHVMMARMTDPDRSF

NinHcG -DET--NGANILGTIIE-ASSDSPNKGFYGSLHNWGHVMMARMHDPDGRF

TtrHcA -DET--TGIDVLGALVE-ASHDSINPEYYGSLHNWGHVITARAHDPEGKF

EbaHcA -DEE--HGVDILGALIE-CSHDSKNPDFYGNLHNLGHVMMAYIHDPDGRF

MgiHcA -DEE--HGVDILGALIE-SSHDSVNKEFYGSLHNWGHIMMSNIHDPDGRF

EbaHcB -DEH--HGTDILGNIIE-SSHDSVNKAYYGSIHNWGHVLMASVNDPDGRY

MgiHcB -DEE--TGINVLGDLIE-SSYDSVNREYYGSIHNWGHVLFAAIKDPDSRF

EbaHcC -DET--SGIDILGDIIE-SSYESVNKSFYGSLHNWGHVMIGDIQDPEGKY

MgiHcC -DEH--NGIDILGDIVE-SSHESVNKEFYGSLHNWGHVLMAAVEDPDGKF

EbaHcD -DEE--HGIDILGALIE-SSEDSQNKRFYGTLHNWGHVILAQAHDPDGRF

MgiHcD -DEE--HGIDILGSLIE-SSHDSMNKRYYGSLHNWSHVVIARIHDPDGRF

EbaHcE -DKD--HGADILGALIE-SSYESKNAGFYGSLHNWGHVMMARVHDPDGRF

MgiHcE -DDE--HGTDILGAIIE-ASHDSKNPFYYGSLHNWGHVMMAAIQDPDGRF

EbaHcF -DAV--HGLDMLGDLLE-SSYESVNREYYGSLHNWGHVILAQVHDPDGRF

MgiHcF -DAE--HGTDLLADLME-SSYESKNREYYGSLHNWGHVIISQCHDPDGRF

EbaHcG -DEK--HGTDILGAIIE-SSFESKNSTFYGSLHNWGHVMMARMHDPDGRF

MgiHcG -DEE--HGADIIGALIE-SSHDSKNSRFYGSLHNWGHVMMARMHDPDGRY

CsaHc1 -DEE--HGADILGALIE-ASSSSPNKGFYGSLHNWGHVMMARMHDPDARF

CsaHc2 -DEA--NGANILGTLIE-ASSSSPNKAFYGSLHNWGHVMMARMHDPDTRF

CsaHc3 -DEA--NGANILGTLIE-ASSSSPNKAFYGSLHNWGHVMMARMHDPDGRF

CsaHc4 -DAE--NGANILGSIIE-ASSSSANKAFYGSLHNWGHVMMARMHDPDGRF

CsaHc5a -DEA--HGADILGTIIE-ASSSSPNKEFYGSLHNWGHVMMARMHDPDGRF

CsaHc5b -DEA--HGADILGTIIE-ASSSSPNKEFYGSLHNWGHVMMARMHDPDGRF

CsaHc6a -DEA--NGANILGTLIE-ASSSSPNKAFYGSLHNWGHVMMARMHDPDGRF

CsaHc6b -DEA--NGANILGTLIE-ASSSSPNKAFYGSLHNWGHVMMARMHDPDGRF

CsaHc6c -DEA--NGANILGTLIE-ASSSSPNKAFYGSLHNWGHVMMARMHDPDGRF

HauHcB -NNK--DGINIISNFVE-SSVDSINRPFYGNIHNYAHVIASRIADPDGKY

HauHcA -NNK--DGINIISNFVE-SSVDSINRPFYGNIHNYAHVIASRIADPDGKY

ScoHcA -DNE--HGIDLLGDAIE-SSLLSVNRAFYGNIHCYAHVMAARIADPDGRY

ScoHcD -NND--HGIDLLGDVVE-SSLLSVNRVFYGNLHCYAHVIAGKVTDPQSTY

ScoHcX CRTRFYGGLNILGNLIE-SNLRSINRMFYGNIHCYAHVIAARVTDPDGKY

ScoHcC -DTE--HGIDELGNIVE-SSLTSLNRDYYGNLHCYAHVIAGRIADPEGKY

ScoHcB -RDE--RGIDVLGNMVE-SSLQSINRPFYGNVHCYAHVIAARIADPDGKY

SpiHc1 -TPE--NGIDIFGHLIA-ATKNSTNRRYYGNLHSYAHVIAARIADSDGEH

AgiHc1 -SPE--TGIDVFGHLIA-ATKNSSNRQYYGNLHSYAHVIAARIADSDGEH

PanHc1 -DKV--NGIDTLAHLII-STTGSVNRRYYGNLHSYAHTIAGKIADASGKY

PanHc2 -NNE--HGIDYLANTIE-ANYDSVNYKLYGNLHCYAHVISAKITDPTTAY

PciHc1 DDV---HGIDVLGDVIE-SSVYSPNVAYYGALHNQAHRVLGAQADPHGKF

PciHc2 DDV---HGIDVLGDVIE-SSVYSPNAAYYGALHNQAHRVLGAQADPHGKF

NkeHc NNT---HGIDILGDLIE-SSVYSPNAQYYGALHNLAHIMLGRQADPHGKY

GpuHc1 NDE---HGIDHLGDIIE-SSLYSPNAQYYGALHNDAHVILGRQADPHGKF

GroHc1 NDE---HGIDHLGDIIE-SSLYSPNAQYYGALHNEAHIILGRQADPHGKF

CcsHc RDE---NGINLLGDIIE-SSVYSLNAGYYGALHNDAHILLGRQSDPHGKF

EpuHc1 RNE---HGIDVLGDVIE-SSTYSPNAGYYGALHNLAHIILGRQGDPHGKF

EpuHc2 RNE---HGIDVLGDVVE-SSTYSPNAGYYGALHNLAHIILGRQGDPHGKF

OscHc1 DND---RGIDFLGDIIE-SSMYSPNIEYYGALHNYAHILLGRQADPKGKF

OscHc3 DND---RGIDILGDIIE-SSMYSPNIPYYGALHNYAHILLGRQADPKGKF

OscHc2 DND---HGIDILGDVIE-SSTSSPNAAYYGALHNYAHIMLGRQADPKGKF

OscHc4 DNE---RGIDILGDVIE-SSTYSPNVEYYGALHNYAHIMLGRQADPKGKF

PleHc NNE---HGIDILGDIIE-SSTYSTNAAYYGALHNQAHRVLGAQSDPKHKF

PleHc2 KNE---HGIDTLGDIIE-SSVYSPNVQYYGALHNTAHIMLGRQGDPHGKF

HamHcA RND---HGIDVLGDIIE-SSVYSPNAQYYGALHNTAHIMLGRQGDPHGKF

PinHcB RQP---KGIELLGDIIE-SSMYSSNVQYYGSLHNTAHAMLGRQGDPHGKF

PinHcA RQP---KGIELLGDIIE-SSKYSSNVQYYGSLHNTAHVMLGRQGDPHGKF

PvuHc RQP---NGIELLGDIIE-SSMYSSNPHYPGSLHNTAHGMLGRQGDPHGKF

PelHc1 RQP---NGIELLGDIIE-SSMYSSNPHYYGSLHNTAHMMLGRQGDPHGKF

PelHc2 RQP---NGIELLGDIIE-SSMYSSNPQYYGSLHNTAHMMLGRQGDPHGKF

PelHc3 RQP---NGIELLGDIIE-SSMYSSNPHYYGSLHNTAHMMLGRQGDPHGKF

PelHc4 RQP---NGIELLGDIIE-SSMYSSNPHYYGSLHNTAHMMLGRQGDPHGKF

CmaHc6 SDS---HGIDVLGDVIE-SSLYSPNPEYYGALHNTAHMMLGRQGDPHGKF

CmaHc1 NND---EGINVLGAAIE-SSTSSIHPSYYGALHNQAHRVLGQQADPHGKF

CmaHc2 NND---EGINVLGAAIE-SSTSSIHPSYYGALHNQAHRVLGQQADPHGKF

CmaHc3 RNN---DGIDVLGDVIE-SSLYSPNPQYYGALHNTAHMMLGRQGDPHGKF

CmaHc4 SDS---HGIDVLGDVIE-SSLYSPNPEYYGALHNTAHMMLGRQGDPHGKF

CmaHc5 SDS---HGIDVLGDVIE-SSLYSPNPEYYGALHNTAHMMLGRQGDPHGKF

CsaHc RDA---HGIDILGDVIE-SSTYSPNPEYYGSLHNTAHVMLGRQGDPHGKF

PinHcC MNS---HGIEFLGDIIE-SSGYSANPGFYGSLHNTAHIMLGRQGDPTGKF

PvaHc1 MNE---RGIDVLGDVIE-SSLYSPNVQYYGALHNTAHIVLGRQSDPHGKY

PvaHc SNE---KGIDILGDIIE-SSLYSPNVQYYGALHNTAHIVLGRQGDPHGKF

FchHc1 MNE---RGIDILGDIIE-SSMYSPNVQYYGALHNTAHIVLGRQADPHGKY

MjaHcL MNE---RGIDVLGDIIE-SSLYSPNVQYYGALHNTAHIVLGRQSDPHGKY

MjaHcY NNE---RGIDILGDIIE-SSLYSPNVQYYGALHNTAHIVLGRQADPHGKY

CjaHc1 NND---HGADILGDIIE-SSNYSPNVHYYGSLHNTAHMVLGRQADPHGKF

PmaHc2 NGT---EGINILGLVVE-SLDHDYNRHYFGKLHSNAHVLLSKVTDPEQKF

StuHc2 NDE---HGINILGEIVE-ASEHSINPDYYGSLHNLAHVMLGRITDPEGKF

PgrHc2 NGT---EGIDVLGLVVE-SLDHDFNSHYFGRLHLNAHVLLSKITDPEQKF

CseHc2 NNT---AGVNLLGEIIE-SSANSVNPVYYGQPHNDGHVMLSKVTDPLQRY

PamHc2 NNT---AGINLLGEIVE-SSTHSVNPVFYGKLHNDGHVMLSKVTDPHLRY

BduHc2 NNT---VGINYLGEVVE-SSVHSVNPVFYGKLHNDAHVLLSKVTDPKLRY

HmeHc2 NNT---EGINVLGEIVE-SSAHSVNPAFYGQLHNDGHVMLSKVTDPKLRY

TdoHc2 NDT---RGIDVLGAVIE-SSEHSQNKELYGSLHNNAHVLLGKVTDYSLKY

StuHc3 EVD---TGINTLGEIIE-ASEHSVNPGFYKSIHTLSHVMLGKITDPEHKY

StuHc1 NNT---HGINTLAEIIE-ASAFSVNPDFYGSIHNMAHIMLGELDDPQGKY

PmaHc1 DAV---HGIDILGDLME-PSVESPHEDYYGSLHNDAHVLLGQITDPLGKF

SamHc1 SSP---EGINVLGELIE-PSTGSKHREYYGALHNYGHIMLGQITDPKRKF

BduHc1 NDT---TGIDTLGALIE-PSVDSKHPEYYGALHNYGHIMLGQITDPKGKF

PamHc1 NNT---QGIDILGCMVE-PSHDSKHPEYYGALHNYGHIMLGQITDPKGKF

CseHc1 NDT---MGINILGSLVE-PSQESPNPQYYGALHNYGHILLGQITDPKGKF

HmeHc1 NNT---KGINTLGEIME-PSFDSKHPEYYGALHNYGHIMLGQIVDPKGKF

CmoHc1 NDT---TGINTLGEMME-PSTTSKHRDYYGALHNYGHILLGKITDPKGKF

CacHc1 NDS---KGIEVLGDLME-PSFDSKHPEYYGALHNYGHILLGQITDPKGKF

TdoHc1 NDT---TGINTLGELIE-PSVFSRHREYYGALHNYGHILLGRITDPKGKF

PgrHc1 DAV---HGIDILGDLME-PSVESPHEDYYGSLHNYAHILLGKVTDPLGKF

ScuHc1 NNT---EGIDKLGAIIE-ASSCSINPSFYGSLHNLGHIILGRVVDPLGKF

MgeHc1 NNSI--EGIETLGRMIE-ASRLSPDIDYYGSIHNLGHILLGEIMDPDHKF

PlaPPO GGDRGVMAFTGTAMRDPIFYRWHKEIDDVFNEYKLTQTPYTNEELI--WN

AfrPPO KENGGPMTDSMTAMRDPIFYRWHKHIDELFYEFKETLGAYTKDELG--FR

DmaPPO SENMGVMGDSVTAMRDPIFYRWHKFIDSLFQQFKATLNPYTAQQLT--LE

DmePPOA1 LEDFGVMGDVTTAMRDPIFYRWHGFIDTVFNKFKTRLNPYNAGELN--FD

DmePPO2 LESFGVMGDVSTAMRDPVFYKWHSYIDRIFQEHKSRLPAYTENQLN--YP

PseHc KAPQGVMGFTETALRDPAFWRWHKMVDNLFNVHKAQLPPYSDNDLT--VS

EpiHc1 NSSDGVMGFVQTALRDPLFYRWHKHIDNLLQNHKRTLQPYTDKELI--AD

EspHc1 QENPGVMCDTSTALRDPIFYRWHKWMDEMFQTYKSTLPSYSKTQLM--FP

PimHc2 KTNPGVMSDTGTSLRDPIFYRWHRFIDNIFQEYKQTLPAYTKDELE--FP

PimHc3A QETPGVMTDTATSLRDPIFYRYHRFIDNMFQEYKSTLPTYTHEQLN--FP

PimHc3B QETPGVMSDTATSLRDPIFYRWHRFIDNIFQEYRRTLPPYTKGQLD--FP

PimHc3C QTNPGVMSDTATSLRDPIFYRWHRFIDNLFQEYKNAQHPYDIDHLG--FT

PimHc4 NENPGVMSDTSTSLRDPIFYRWHRFVDNIFQDYRSSLPVYTREQLD--FP

PimHc5A RENPGVMSDTSTSLRDPIFYRFHRFMDNIFQEYKATLPSYTEQELD--FP

PimHc5B QTNPGVMSDTATSLRDPIFYRWHRFIDDMIQEYKENLPVYDKRQLG--FT

PimHc6 QENPGVMSDTSTSLRDPIFYRWHRFIDNIFQEYKCSLHSYTKEELS--FP

AauHc6 QENPGVMSDTSTSLRDPIFYRWHRFIDNIFQEHKKSFHPYTKEELS--FP

LpoHcII HEEPGVMSDTSTSLRDPIFYNWHRFIDNIFHEYKNTLKPYDHDVLN--FP

LpoHcIIIa HENPGVMSDTSTSLRDPIFYRWHRTLDNLFQEYKESLSPYTKEELS--FP

LpoHcIIIb HDNPGVMSDTATSLRDPIFYRFHRYIDNMFQAYKATLHSYEHHQLI--FN

LpoHcIV HENPGVMSDTSTSLRDPIFYRYHRFIDNMFQEYKASLHHYTKTELT--FP

LpoHcVI HLNPGVMSDTATSLRDPIFYRWHRFIDNMFQDYKETLPHYNHSDLE--FP

CroHcVI HLNPGVMSDTATSLRDPIFYRWHRFIDNMFQNYKETLPHYNHSDLE--FA

CroHcV HLNPGVMSDTATSLRDPIFYRWHRFIDNLFQDYKETLPHYRKDELE--FP

CroHcIV HENPGVMSDTSTSLRDPIFYRYHRFIDNMFQEYKASLHHYTKKELT--FP

CroHcIIIb HDNPGVMSDTATSLRDPIFYRFHRYIDNMFQTYKATLHSHEHHQLT--FS

CroHcIIIa YENPGVMSDTSTSLRDPIFYRWHRTLDNLFQEYKESLSPYTKEELS--FP

CroHcII HEEPGVMSDTSTSLRDPLFYNWHGFIDDLFQEYKNTLKPYDHGMLN--FT

CroHcI HEDPGVMSDTSTSLRDPIFYRWHRFIDNVFQDYKESLHPYTKDELS--FT

AgoHcA RETPGVMTDTATSLRDPIFYRFHRFIDNVFQEYKKTLPVYNKDNLD--FP

AgoHcB QLNPGVMSDTATSLRDPIFYRWHRFIDDMFQEYKKSLTPYS-SQLQ--FK

AgoHcC QTNPGVMDDTATSLRDPIFYRWHRFIDDMFQEFKRKLKPYTKDELS--FT

AgoHcD RTNPGVMDDTSTSLRDPIFYRYHRWMDNIFQEYKHRLPSYTHQQLD--FP

AgoHcE NENPGVMSDTSTSLRDPIFYRYHRFIDNIFQKYIATLPHYTPENLT--CP

AgoHcF RENPGVMSDTSTSLRDPIFYRYHRFIDNMFQEYKATLPCYEKKDLE--FN

AgoHcG EENPGVMSDTSTSLRDPIFYRWHRFVDNIFQQYKATLPHYTPDDLN--FP

AgoHcX QQLGGVIGDTTTAIRDPIFFKLHRFVDNLFTEHKISLPPYTKEELN--FA

EcaHcA RETPGVMTDTATSLRDPIFYRYHRFIDNVFQEYKKTLPVYSKDNLD--FP

NinHcA RETPGVMTDTATSLRDPIFYRFHRFIDNVFQEYKKTLPVYSRDNLT--FP

EcaHcB QLNPGVMSDTATSLRDPIFYRWHRFIDDMFQEYKKSLTPYS-SQLQ--FK

NinHcB KLNPGVMDDTATALRDPIFYRWHRFIDDLFQEYKKTLPPYTKDELS--FG

EcaHcC QTNPGVMDDTATSLRDPIFYRWHRFIDDMFQEFKRKLKPYTKDQLS--FT

EcaHcD RTNPGVMDDTSTSLRDPIFYRYHRWMDNIFQEYKHRLPSYTHQQLD--FP

NinHcD KENPGVMDDTSTALRDPIFYRYHRWMDNIFQEYKRRLSSYTPQDLS--FP

EcaHcE NENPGVMSDTSTSLRDPIFYRYHRFIDNIFQKYIATLPHYTPEDLT--CP

NinHcE NENPGVMSDTSTSLRDPIFYRYHRFIDNIFQDYKQTLKPYDKAQLD--FP

EcaHcF RENPGVMSDTSTSLRDPIFYRYHRFIDNIFQEYKATLPCYEKKDLE--FS

NinHcF KENPGVMSDTSTSLRDPIFYRYHRFIDNIFQEYKATLPVYDKKDLD--FP

EcaHcG EENPGVMSDTSTSLRDPIFYRWHRFVDNIFQEYKATLPSYTADDLN--FP

NinHcG QENPGVMSDTSTSLRDPIFYRWHRFVDNIFQQYKSTLQPYTWEQLS--FP

TtrHcA HENPGVMSDTSTSLRDPIFYRWHRFIDNVFQDYKESLHPYTKHELS--FT

EbaHcA QETPGVMTDTATSLRDPIFYRFHRFIDNMFLEYKATLPSYTKEQLS--FA

MgiHcA QETPGVMTDTATSLRDPIFYRFHRFIDNVFQEYKVSLPEYTREQLN--FP

EbaHcB KVNPGVMSDTATSLRDPIFYRWHRFIDDMFQEFKKSLHSYTKDELE--FH

MgiHcB QLNPGVMSDTATSLRDPIFYRWHRFIDDMLLEYKKSLPHYTKHQLG--FT

EbaHcC QTPSGVMSDTATSLQDPIFYRWHRFIDNIFLEYKSKLHSYNKDDLS--FN

MgiHcC QCNPGVMDDTATSLRDPIFYRWHRFLDDLFQEFKSKFHPYNKKELE--FP

EbaHcD KENPGVMDDTSTALRDPIFYRLHRFMDNIFQEYKHSLKPYDRHDLE--FP

MgiHcD MEAPGVMDDTTTSLRDPIFYRLHRFMDNIFQDYKHRLHPYTHDELD--FP

EbaHcE QENPGVMSDTSTSIRDPIFYRYHRFVDNIFQDFKLSLNPYTKEELD--FP

MgiHcE KENPGVMSDTSTSLRDPIFYRYHRFVDNIFQQYKDTLHHYTKDDLL--FS

EbaHcF KENPGVMSDTSTALRDPIFYRYHRFVDNIFQEYKISLPHYQKKDLD--FP

MgiHcF KENPGVMSDTSTSLRDPIFYRYHRFVDNIFQELKASLPPYKQSELD--FT

EbaHcG QENPGVMSDTSTALRDPIFYRWHRFIDNIFQDYKATLPHYTKDDLS--FD

MgiHcG QENPGVMSDTSTSLRDPIFYRWHRFVDNIFKDYKSTLHPYEKEEYS--FD

CsaHc1 QENPGVMSDTSTSLRDPIFYRWHRFVDNIFQQYKATLHPYTKEELS--FA

CsaHc2 QENPGVMSDTSTSLRDPIFYRWHRFVDNIFQQFKATLHSYTPEDLS--FR

CsaHc3 QENPGVMSDTSTSLRDPIFYRWHRFVDNIFQQYKATLHQYTKEQLS--FA

CsaHc4 QENPGVMSDTSTSLRDPIFYRWHRFVDNIFQQYKATLKPYTKEELS--FE

CsaHc5a QENPGVMSDTSTSLRDPIFYRWHRFVDNIFQQYKATLHPYTQEDLS--FP

CsaHc5b QENPGVMSDTSTSLRDPIFYRWHRFVDNIFQQYKATLHPYTQEDLS--FP

CsaHc6a QENPGVMSDTSTSLRDPIFYRWHRFVDNIFQQYKATLHPYTKEELS--FE

CsaHc6b QENPGVMSDTSTSLRDPIFYRWHRFVDNIFQQYKATLHPYTKEELS--FE

CsaHc6c QENPGVMSDTSTSLRDPIFYRWHRFVDNIFQQYKATLHPYTKEELS--FE

HauHcB GEDNGVMYDVSTSARDPLFYSWHKFINSLFIEHKDMLPAYTHDDLE--FK

HauHcA GEDNGVMYDVSTSARDPLFYSWHKFINSLFIEHKDMLPEYTHDDLE--FK

ScoHcA GEDNGVMHDVATSARDPLFYRWHKFIDNIFLEYKDNLDPYTQYELT--WP

ScoHcD GEKNGAMYDVATSARDPLFYSWHKFIDNIFQEHKETLQPYNKDELN--FP

ScoHcX GQGNGVMYDVATSARDPLFYQWHKFLDHFFYEHLTKLPTNHLFHLQ--NP

ScoHcC GEDNGVMYDVATSARDPLFYRWHKYIDNIFQEYKNTLPPYTTEELTPQNS

ScoHcB GEDNGAMYDVATSARDPLFYQWHKFIDNLFHEYKDALKAYSSEDLT--YN

SpiHc1 MEDNGAMYDVATSARDPLFYSWHKFIDKLFTEYQMTLTPYTPYQLT--WP

AgiHc1 KEDNGAMYDVATSARDPLFYSWHKFIDKIFTEYQNTLTPYTPYQLT--WP

PanHc1 AEDAGVMIDVTTSARDPIFYQWHKYIDGLFQTYQKTLKPYNKYELS--FP

PanHc2 NEDYGVMYDVATSARDPLFYRWHKFINKFFSEHKKMLKPYTKDELD--FP

PciHc1 NMSPGVMEHFETATRDPAFFRLHKYMDNIFKEHKDKLPAYSKEELQ--YT

PciHc2 NMPPGVMEHFETATRDPAFFRLHKYMDNIFKEHKDKLHPYTKEELQ--YA

NkeHc KMPPGVMEHFETATRDPSFFRLHKYMDNIFKEHKDSLPAYSSDELG--FD

GpuHc1 NLPPSVMEHFETATRDPAFFRLHKYMDNIFKEHKDSLPPYTAEEIG--FP

GroHc1 NLPPSVMEHFETATRDPAFFRLHKYMDNMFKEHKDSLAPYTAEEIG--FP

CcsHc NLPPGVMEHFETATRDPAFFRLHKYMDGIFKEHKDTLPAYTKEEIE--FS

EpuHc1 NMPPGVMEHFETATRDPSFFRLHKYMDGIFKEHKDSLPPYTKEEIE--FP

EpuHc2 NMPPGVMEHFETATRDPSFFRLHKYMDGIFKEHKDSLPPYTKEEIE--FP

OscHc1 NMPPGVMEHFETATRDPSFFRLHKYMDNIFKEHKDKLCPYKTNDLL--YS

OscHc3 NMPPGVMEHFETATRDPSFFRLHKYMDNIFKEHKDKLPPYKTNDLL--YS

OscHc2 NMPPGVMEHFETATRDPSFFRLHKYMDGLFKLHKDKLKPYTHDDLA--YD

OscHc4 NMPPGVMEHFETATRDPSFFRLHKYMDNIFKLHKDKLTPYTHEELA--YD

PleHc NMPPGVMEHFETATRDPAFFRLHKYMDGIFKEHKDNLPPYTEEDLL--YS

PleHc2 DMPPGVMEHFETATRDPSFFRLHKYMDNIFKEHKDTLPPYTKEDIA--FP

HamHcA NMPPGVMEHFETATRDPSFFRLHKYMDNIFKEHKDSLPPYTKADIE--FT

PinHcB NLPPGVMEHFETATRDPSFFRLHKYMDNIFKKHTDSFPPYTHDDLE--FA

PinHcA NLPPGVMEHFETATRDPSFFRLHKYMDNIFKKHTDSFPPYTHDNLE--FS

PvuHc NMPPGVMEHFETATRDPSFFRLHKYMDNIFKEHTDSFPPYTHEDLE--FP

PelHc1 DMPPGVMEHFETATRDPSFFRLHKYMDNIFKEHTDSFPPYTHEDLE--FP

PelHc2 DMPPGVMEHFETATRDPSFFRLHKYMDNIFKEHTDSFPPYTHEDLE--FP

PelHc3 NMPPGVMEHFETATRDPSFFRLHKYMDNIFKEHTDSFPPYTHEDLE--FA

PelHc4 DMPPGVMEHFETATRDPSFFRLHRYMDNIFKEHTDSFPPYTHEDLE--FP

CmaHc6 DLPPGVLEHFETATRDPAFFRLHKYMDNIFREHKDSLTPYTKDELE--FP

CmaHc1 NMPPGVMEHFETATRDPSFFRLHKYMDNIFKEQKDKLPPYTTDDLK--YD

CmaHc2 NMPPGVMEHFETATRDPSFFRLHKYMDNIFKEQKDKLPPYTTDDLK--YD

CmaHc3 DLPPGVLEHFETATRDPAFFRLHKYMDNIFRKHKDSLPPYTKEELN--FE

CmaHc4 DLPPGVLEHFETATRDPAFFRLHKYMDNIFREHKDSLTPYTKDELE--FP

CmaHc5 DLPPGVLEHFETATRDPAFFRLHKYMDNIFREHKDSLTPYTKDELE--FP

CsaHc ALPPGVLEHFETATRDPAFFRLHKYMDNIFREHKDSLTPYTKDDLE--FS

PinHcC DLPPGVLEHFETSTRDPSFFRLHKYMDNIFREHKDSLTPYTRDELE--FN

PvaHc1 ALPPGVLEHFETATRDPSFFRLHKYMDNIFKEHKDSLPPYTVEELT--FA

PvaHc DLPPGVLEHFETATRDPSFFRLHKYMDNIFKEHKDNLPPYTKADLE--FS

FchHc1 ALPPGVLEHFETATRDPSFFRLHKYMDNIFKEHKDSLPPYSKEELT--FT

MjaHcL DLPPGVLEHFETATRDPSFFRLHKYMDNIFKEHKDTLPPYTAEELT--FA

MjaHcY DLPPGVLEHFETATRDPSFFRLHKYMDNIFKEHKDTLTPYTKADLE--FA

CjaHc1 GLPPGVMEHFETATRDPGFFRLHKYMDNIFREHKDSLPPYKTEHLL--FD

PmaHc2 GTPPGVMEHFETATRDPAFFRLHKHIDNLFKQHKDMLTPYTKEELD--FP

StuHc2 DAPPGVMEHFETATRDPSFFRLHKYIDNIFKSHKDHLTPYTHKELE--FP

PgrHc2 GTPPGVMEHFETATRDPAFFRLHKHIDNLFKMHKEMLSPYTKEELD--FP

CseHc2 GVPPGVMEHFETATRDPAFFRLHKHVDNLFKLHKDLLLPYDVSELD--FP

PamHc2 GVPPGVMEHFETATRDPAFFRLHKHVDNLFKFHKDLLVPYSFHELE--FP

BduHc2 GVPPGVMEHFETATRDPAFFRLHKHVDNIFKLHKDLLPPYDVSELE--FP

HmeHc2 GLPPGVMEHFETATRDPAFFRLHKHVDNLFKMHKDFLPPYQTHELL--FP

TdoHc2 GLPPGVMEHFETATRDPAFFRLHKLVDNLFKEHKDLLPPYTKEDLA--FP

StuHc3 GMPPGVMEHFETSTRDPAFFRLHKYIDNLFRTYKEHLPPYTKEELA--VD

StuHc1 GTPPGVMEHFETATRDPSFFRLHKYIDSFFKEHKDSLPPYTTDELD--FH

PmaHc1 DLPPGVMEHFETATRDPAFFRLHKHIDNLFKMYKDLLPPYTKAELE--FP

SamHc1 NMPPGVMEHFETATRDPAFFRLHKYIDNLFYEHKDLLPRYTSEELE--LP

BduHc1 NMPPGVMEHFETATRDPAFFRLHKYIDNLFKLHKDLLPKYTQAELS--FP

PamHc1 NMPPGVMEHFETATRDPAFFRLHKYIDNLFKLHKDLLPHYTMEELG--FN

CseHc1 DMPPGVMEHFETATRDPAFFRLHKYIDNLFKLHKDMLPPYAREELE--FP

HmeHc1 NMPPGVMEHFETATRDPAFFRLHKYIDNLFKIHKDLLPVYTHEELD--FP

CmoHc1 NMPPGVMEHFETATRDPAFFRLHKHIDNLFKLHKDLLPPYKQEELV--LP

CacHc1 NMPPGVMEHFETATRDPAFFRLHKYIDNLFKLHKDLLPSYSKEELS--LD

TdoHc1 NMPPGVMEHFETATRDPAFFRLHKYIDNLFKEHKDHLPHYTRDELL--LP

PgrHc1 DLPPGVMEHFETATRDPAFFRLHKHIDNLFKMHKDLLPPYTKAELE--FP

ScuHc1 GMPPGVMEHFETATRDPAFFRLHKHIDNIFKEHKDSLVPYTHEELD--VE

MgeHc1 NLPPGVMEHFETAMRDPVFFSLHKHIDYIFKHYKDTLVPYKREELD--FP

PlaPPO QVKVTGVSVA----------------------------GEISTSPNL--L

AfrPPO GITVMDVSV-------------------------------SN--GNR--I

DmaPPO GITVNNVEVR---------------------RNATSDNPRSNPPKNL--I

DmePPOA1 GITVDYIEAK---------------------------IGKSNTKANT--L

DmePPO2 GVSIAGIQVD-----------------------------TNGGRPNN--L

PseHc DITISDVSIK------------------------SGKSPRKTYSLNE--L

EpiHc1 KIEIKEASIT---------------------------SSQTKDPKNK--L

EspHc1 SVKVNNVTLN----------------------------ASI---PNV--V

PimHc2 GVNVVGVSVN----------------------------AKN---ANS--I

PimHc3A EVEVTGVTVT----------------------------ART---DDV--I

PimHc3B NVKVTDIKIN----------------------------ART---PNV--I

PimHc3C GIEIKSVAVE----------------------------TDG---HRLHDV

PimHc4 GVEITNVTVN----------------------------AKE---AGK--I

PimHc5A DVHVAGVTVR----------------------------AKV---PDV--V

PimHc5B GVEVKSINVK----------------------------GAA---PNV--V

PimHc6 GIIVEGVTID----------------------------AKA---QNL--I

AauHc6 GVEVVGVSIN----------------------------SKT---ANV--I

LpoHcII DIQVQDVTLH----------------------------ARV---DNV--V

LpoHcIIIa GVEVLSATVK----------------------------AKT---DNV--I

LpoHcIIIb GVQIDNVTVK----------------------------AKH---TNV--I

LpoHcIV EISISHVTIK----------------------------AKV---PNI--I

LpoHcVI GIEITKVLLN----------------------------AEH---TNN--I

CroHcVI GVEITKVLVR----------------------------AKH---TNN--I

CroHcV GVEITNVIVS----------------------------AEH---ANN--I

CroHcIV EISVSHVTVK----------------------------AKV---PNI--I

CroHcIIIb GVQIDNVTVK----------------------------AKQ---PNV--I

CroHcIIIa GIEVLSASVK----------------------------TKT---NNV--I

CroHcII DIQIQNVTLH----------------------------ARV---DNV--I

CroHcI GIKVVNVSVN----------------------------AKV---KNV--I

AgoHcA QVTITEVKVK----------------------------AKI---PNV--V

AgoHcB GVAVKSVCVR----------------------------AKT---ADV--V

AgoHcC GVEIKTVKVH----------------------------ALQ---EDV--I

AgoHcD GVRISRVTVK----------------------------SKV---PNL--I

AgoHcE GVHVVNVTVN----------------------------AKV---PNV--V

AgoHcF GVEIVNVTVN----------------------------AKA---PNV--I

AgoHcG GLRIVSVQVN----------------------------AKS---QNR--V

AgoHcX GVVIDEVVVE----------------------------GRE---PNV--I

EcaHcA QVTITDVKVK----------------------------AKI---PNV--V

NinHcA DIEITESKVN----------------------------AKI---TNV--I

EcaHcB GVIVKSVCVR----------------------------AKT---ADV--V

NinHcB NVFVKSLNVK----------------------------AEQ---PNT--V

EcaHcC GVEIKNVKVH----------------------------AHE---ENV--I

EcaHcD GVRISRVTVR----------------------------SKV---PNL--I

NinHcD GVRVVNVTVN----------------------------AKV---PNL--I

EcaHcE GVHVVNVTVN----------------------------AKV---PNV--V

NinHcE GVQVVNVTVN----------------------------AKV---PNL--V

EcaHcF GVEIVNCTVN----------------------------AKA---PNV--I

NinHcF GVSVVNVTVN----------------------------AKL---PNI--V

EcaHcG GLRIVSVQVN----------------------------AKS---QNR--V

NinHcG GVKAVSCEVK----------------------------AKQ---NNV--I

TtrHcA GIKVVNVSVN----------------------------AKVITARNV--I

EbaHcA DVEVVGVKVN----------------------------AID---TDV--V

MgiHcA DVEVVGARVH----------------------------SDI---DNV--I

EbaHcB GVEVKSVSVK----------------------------AST---TNT--I

MgiHcB GVEIKSTCIR----------------------------AKK---TDE--I

EbaHcC GVEIVNVQLK----------------------------ENP---DKI--V

MgiHcC GVKIESVAIE----------------------------SKE---HDV--I

EbaHcD GIRIQNVTVK----------------------------ARV---ENI--V

MgiHcD GIHILSTTVK----------------------------SKV---ANV--I

EbaHcE GIEVVNVTVT----------------------------AKV---PNL--V

MgiHcE GIEVVSVAVN----------------------------AKV---QDL--V

EbaHcF GVKVVNVTVN----------------------------AKV---PNV--I

MgiHcF GIDVVNVTVN----------------------------AKA---PNL--V

EbaHcG GIKVTDVVVK----------------------------AKS---DNV--I

MgiHcG GIEVLEVTVK----------------------------AKT---DNV--L

CsaHc1 GVKIVNAYVK----------------------------AKK---TNV--V

CsaHc2 GIKVLSAQVN----------------------------AKQ---ANV--V

CsaHc3 GIKILSTQVN----------------------------AKQ---ANV--V

CsaHc4 GIKVASIQVN----------------------------AKQ---ANV--V

CsaHc5a GIKVLSAQVN----------------------------ARQ---ANV--V

CsaHc5b GIKVLSAQVN----------------------------ARQ---ANV--V

CsaHc6a GIKVLSAQVN----------------------------AKQ---PNV--V

CsaHc6b GIKVLSAQVN----------------------------AKQ---PNV--V

CsaHc6c GIKVLSAQVN----------------------------AKQ---PNV--V

HauHcB DVVVDTISVK----------------------------GKEETIPNI--L

HauHcA DVVVNTVAVK----------------------------GKEETQENI--L

ScoHcA DVVLNDVTVK----------------------------PHKGDYDDE--V

ScoHcD DVQVDSLRIN----------------------------VANGTYENI--V

ScoHcX DVSITNLEII------------------------------SNGRKNE--I

ScoHcC EFRVQGISVV-----------------------------GETSARDT--V

ScoHcB DITIEEVNVQ----------------------------GEGGSPANT--V

SpiHc1 DVVVDGIHIE----------------------------NLKTHEENM--I

AgiHc1 DVVIDGIHIE----------------------------NLKTHEENM--I

PanHc1 GVSIEDVHLETESDRKSEHHDDHHDDKHDDKHNESHDDHHDDHHEDV--I

PanHc2 GVKVEKVSVQ-----------------------------TSTSEPNL--I

PciHc1 NVQITGVDV------------------------------------GE--L

PciHc2 NVQITEVDV------------------------------------GE--L

NkeHc GVSLDSVAI-----------------------------------DGT--L

GpuHc1 GVHVTGVSI-----------------------------------EGE--L

GroHc1 GVHLTSVSI-----------------------------------EGE--L

CcsHc GISLEKISV-----------------------------------DGP--L

EpuHc1 DIAINAVGV-----------------------------------KGE--L

EpuHc2 DVAITNVGV-----------------------------------KGD--L

OscHc1 NIEITDVSI-----------------------------------EGE--L

OscHc3 NIEIKDVSI-----------------------------------DGE--L

OscHc2 NAHITEVHI-----------------------------------EGE--M

OscHc4 NAHITDVKI-----------------------------------DGE--L

PleHc NVKITGVDV------------------------------------TE--L

PleHc2 GVVLDSVTI-----------------------------------DGE--L

HamHcA GVAVEEVEI-----------------------------------VGQ--L

PinHcB GMVVDGIAI-----------------------------------DGE--L

PinHcA GMVVNGVAI-----------------------------------DGE--L

PvuHc GVSVDNIAI-----------------------------------EGH--L

PelHc1 GVSVDNIAI-----------------------------------DGH--L

PelHc2 GVSVENIAI-----------------------------------DGH--L

PelHc3 GVSVDNIAI-----------------------------------DGH--L

PelHc4 GVSVENIAI-----------------------------------DGH--L

CmaHc6 GVKIDSISL-----------------------------------SNR--L

CmaHc1 NVEITDVDI------------------------------------DE--L

CmaHc2 NVEIKSVDV------------------------------------DE--L

CmaHc3 GVNIDNFYI-----------------------------------KGN--L

CmaHc4 GIEIDSFSL-----------------------------------SNR--L

CmaHc5 GVAVESISL-----------------------------------KNR--L

CsaHc GISVDSLDI-----------------------------------SNR--L

PinHcC GVSIDSIAI-----------------------------------EGT--L

PvaHc1 GVSVDSVAI-----------------------------------EGE--L

PvaHc GVSVTELAV-----------------------------------VGE--L

FchHc1 GVNVENLSV-----------------------------------DGE--L

MjaHcL GVSVDSIAI-----------------------------------EGA--L

MjaHcY GVSIDNVAV-----------------------------------EGE--L

CjaHc1 GIVVDNFGV-----------------------------------DGA--L

PmaHc2 GVTVDAVKVV---------------------------GKSEDSTANQ--I

StuHc2 GVTVTAAKVV---------------------------GLSHASTPNM--L

PgrHc2 GVIVDAVKVV---------------------------GKSEDSTANQ--I

CseHc2 GVKIEAVKVV---------------------------GSCKASTPNQ--L

PamHc2 GVKIEAVKVI---------------------------GTCKASTPNH--L

BduHc2 GVKVEAVKVI---------------------------GTCKASTPNQ--L

HmeHc2 EVTVEAVKVI---------------------------GVCKASIPNH--L

TdoHc2 GVSIDAVKVV---------------------------GTCKASTPNQ--L

StuHc3 GLKVNDVEV------------------------------------TE--L

StuHc1 GVEIVDVEV------------------------------------DK--L

PmaHc1 GVKVLDWEI------------------------------------GN--L

SamHc1 GTSIEEVQI------------------------------------DP--L

BduHc1 GVKVSDVTV------------------------------------DE--L

PamHc1 GVTIQDLTV------------------------------------DD--L

CseHc1 GVAIQDVTV------------------------------------DD--L

HmeHc1 GLKVVDLDV------------------------------------DV--L

CmoHc1 GVKITDVAV------------------------------------EP--L

CacHc1 GVQIEDVQI------------------------------------DE--L

TdoHc1 GVSINSVEV------------------------------------DE--L

PgrHc1 GVKVLDWEI------------------------------------GN--L

ScuHc1 GVDIKNVEV------------------------------------DD--L

MgeHc1 GVKVENVEV------------------------------------DR--L

PlaPPO RTFWQEKRVPLSRGVDFGRP-N--PVYVLTRHLQHEPFLYNIAVHNSTDK

AfrPPO TTGWGKSRLNLAQGIDLQGR-R--NVQAELTHLIHEPFAYRFTVRNDTRA

DmaPPO ITGWQESIVELDRGLDFSSL-R--PVQARVTHLQHEDFSYRIQVTNSTKT

DmePPOA1 LTYWQKSSADLAAGLDFGPT-TDRNIFASFTHLQNAPFTYTFNVTNN-GA

DmePPO2 TTFWQQSDVDMSRGFDFLPR-G--NVFARFTHLQHLPFTYTISLNNDSGA

PseHc ATFFKQRVIDISRGLDFEADSS--TATVNLTHLDHLPFTYTIKVTNNADN

EpiHc1 YTFFDTKQLNLTKGLDFGNLEQ--SILVTITHLQHEEFTYNIKVNNNTGK

EspHc1 GTFWKTAELEMSEGINFGKK-G--SVKCQYQHIDHEPFSYLIEVDNANFK

PimHc2 ETFMKEDELELSYGIHL--Q-G--SVKVLHKHLDHEEFHYTINIDNTTGT

PimHc3A NTFNREAELVVTHAQHIGK--G--PVKVRYEHLDHESFSYAIDALNNGNA

PimHc3B DTFMTKDELEMSYGFAFGHA-G--SYKARYEHLDHEHFSYTINVNSTLPA

PimHc3C HTSFATSILNISSAYPLDVE-V--PIKVRWPHLTHEPFTYNISVVNHTGR

PimHc4 HTFMKDAELELSHGIKFPSD-H--SVKVHYKHLDHEPFSYTIECNNKGAS

PimHc5A TTFMNEEELELSNGINT--K-G--SVKVKFEHLDHESFDYEIAVENKSHD

PimHc5B NTFFTEDVLDVSHSINFDRK-G--AVKVRHHHLDHERFTYKIEVFNQGTK

PimHc6 NTFTKEAELELSHGINFGTN-S--SVKVKYHHLDHEPFSYKISVNNETGG

AauHc6 TTLIKESLLELSHGINFGTD-Q--SVKVKYHHLDHEPFTYNIVVENNSGA

LpoHcII HTFMREQELELKHGINPGNA-R--SIKARYYHLDHEPFSYAVNVQNNSAS

LpoHcIIIa ITSMVESELELTHGINFGTD-H--SVKVKYNHLDHESFSYQIKVENTSGS

LpoHcIIIb NTYTTESELELSHGIDM--K-A--STKVKYHHLDHDKFEYSISVNNTSGL

LpoHcIV HTFMATDELELSHGIHL--D-G--STKVKYHHLNHEAFSYEIQVENHSDT

LpoHcVI NTFKEFSVLDLSHAYEFGRD-G--AVKVRFSHLNHEDFHYKIQVENHTSK

CroHcVI DTFKSNSLLDLSHAYEFGRT-G--AVKVRFNHLNHEEFDYKIQVENHTSK

CroHcV NTFKENALLNLSHAYEFGRT-G--NVKVRYNHLSHESFDYNIEVENHSNR

CroHcIV HTYMATDELELSHGIHL--N-G--PTKVKYHHLNHEPFTYEIHVENKSDS

CroHcIIIb NTYTTESELELSHGIHM--K-G--PTKVKYHHLDHDKFDYSISVNNTSGL

CroHcIIIa TTSMVESELELSHGINFGTD-H--SVKVKYHHPDHEPFSYHINVSNNSGS

CroHcII HTFMRENELELKYGVNLGHA-R--SVKARYYHLDHEPFSYAVDVQNNSAS

CroHcI TTFIKKNELELTHGMDFGTT-N--SVKVKYPHLEHEPFSFQITVENTSGA

AgoHcA HTFIREDELELSHCLHFAKP-G--SVRARYHHLDHESFSYIINAQNNSNA

AgoHcB ETTFETSLLDISHAFNFGRS-G--PVKVRYNHLTHEPFTYKIAVDNAGTK

AgoHcC TTTFVEDLLEISNAFNFGRT-G--AVKVRYHHLDHEPFVYDIEVENHSAR

AgoHcD HTYSTDSLLELSHGISL--K-G--HIQVKYEHLDHEPYNYEIEVDNRTGE

AgoHcE TTFMKEAELDLSYGIDFGSD-H--SVKVLYRHLDHEPFTYNIGIENSTGG

AgoHcF NTYMKESTLEMSHGISF--K-G--GVKVKYSHLDHDPFTYSISVENTTGD

AgoHcG RTFLKQEELVLSHGISFGTD-H--TVKVHYNHLDHEPFSYTINVDNSSGA

AgoHcX FTHMRKATLELSHAIRL--K-I--SVMVEYEHLDHESFIYTIKAKNMTKT

EcaHcA HTFIREDELELSHCLHFAKP-G--SVRARYHHLDHESFSYIISAQNNSNA

NinHcA HTFIREDELELTHCMNFGSP-G--SVKARYHHLDHESFSYIFNANNNGSE

EcaHcB ETTFANALLDISHAFNFGRT-G--PVKVRYNHLTHEPFTYKIVVDNAGTK

NinHcB KTFFREDFLDVSHAFYFGRT-G--SVKVRYQHLDHEPFTYQFVVENTGTK

EcaHcC TTTFVEDLLEISNAFNFGRT-G--AVKVRYQHLDHEPFVYDIEVENHSAR

EcaHcD HTYSKDSLLELSHGINL--K-G--HIQVKYEHLDHEPYNYEIEVDNRTGE

NinHcD HTYSKDAYLELSHGINL--K-N--KIKVKYEHLDHEPFSYTIAATNATGA

EcaHcE TTFMKEAELELSYGIDFGSD-H--SVKVLYRHLDHEPFTYNISVENSSGG

NinHcE TTFMKTDDLELTHGIDFGTT-K--SVKVKYQHLDHEPFTFNISVDNQSGG

EcaHcF NTYMKESTLEMSHGISF--K-G--AVKVKYQHLDHDPFTYSISVENTTGD

NinHcF NTYLKEDQLEVSHGVSL--K-G--SVKVKYHHLDHEPFAYNISCENSSGA

EcaHcG RTFLKQEELVLSHGINFGTE-H--TVKVHYNHLDHEPFSYTINVDNSSGA

NinHcG TTFIKDEELTLSHGINFGTD-R--KVKVKYHHLDHEPFSITVNAQNSSGA

TtrHcA TTFIKKNELELTHGIDFGTT-N--SVKVKYPHLEHEPFSFQITVENTSGA

EbaHcA RTFTREDELELVHCMNFGSP-R--SVRARYHHLDHESFTYTINVQNNSTA

MgiHcA HTYMVMDELELSHCMNFGTA-A--SVKARYHRLDHDAYSYSISVQNNGST

EbaHcB TTVMKENSLRTSHAFYFGRT-G--DMGVKYKHLDHEPFKYKIEVIDQGTK

MgiHcB TTFFTKDELNISRAYNMART-G--AIKIRYQHIDHEPFTYKINVFNKGSK

EbaHcC STTFKKDLLNLSYAYYLDRK-G--DIKVRYEHLDHDPFTYEIAVENHSRK

MgiHcC HTFFAEDTIDVTYAYNFRRP-A--PVKVRLHHLDHEPFNYNIIATNQSAN

EbaHcD NTFMKEDELELSHGINL--K-G--SVRVKYEHIDHEPYKYHIEVDNTTGA

MgiHcD HTTLKQDSLELHHGIPL--K-G--SVKVKYEHLDHDQFDYSIVVDNKTGK

EbaHcE TTYLKEAELELHHGIDFGTT-H--SVKAAYKHLDHEPFAYNISVENKTGG

MgiHcE TTFMKDSELELTYGINLGTT-H--SVSVRYHHLDHEPFTFNISVNNNTGE

EbaHcF NTFLKESQLELSHGITL--K-G--SVKVSYHHLDHDPFTFSIGVENTSGE

MgiHcF HTFFAEDQLELRHGINT--H-G--SVKVKYHHLDHEPFTYNISVENKTGA

EbaHcG TTFMKEDELDLSHGINFGTN-H--KVKVRYQHLDHEPFSISITVNNTTGA

MgiHcG TTFLKEDILELTHGINLGTT-H--SVKVTYDHLDHEPFHYSIDVQNKTGA

CsaHc1 TTFMKDDHLDLSHGIEFGTE-H--KVLVKYHHLDHEPFSLIINVDNTTSE

CsaHc2 TTFMKNDLLDLSHGINFGTG-H--KVQVQYNHLDHEPFSLIINVENDTGA

CsaHc3 TTYLKEDRLDLSHGINFGTG-H--KVYVKYHHLEHEPFSLIINVENNTGA

CsaHc4 TTFMKDDTLDLSHGIHFGDG-Q--SVKVQYHHLDHEPFSFIINVENNSGS

CsaHc5a TTYMKDDLLDLSHGINFGTG-H--KVHVEYHHLDHEPFSLIINIENDSGT

CsaHc5b TTYMKDDLLDLSHGINYGTG-H--KVQVEYHHLDHEPFSLIINIENDSGT

CsaHc6a TTYIKDDKLDLSHGINFGTG-H--RVYVKYHHLDHEPFSVIINVENNSGA

CsaHc6b TTYIKDDKLDLSHGINFGTG-H--RVYVKYHHLDHEPFSVIINVENNSGA

CsaHc6c TTYIKDDKLDLSHGINFGTG-H--RVYVKYHHLDHEPFSVIINVENNSGA

HauHcB KTFYRYRTIPLPTGFTFNHE-K--KVDVEHRYLDHEEFEYRLDITNNAAA

HauHcA TTFYKYRSFALPTGFTFNHE-K--HVEIEHRHLDHEEFEYKLDITNNAAT

ScoHcA HTYWEVDNYELGKGFDYTRK-T--TATVKVRHLQHEDYHYEIDIDNNAGK

ScoHcD RTYWQNSLFKIAKGFTFTTE-G--SVLVKVKHLNHETFYYNLEVTNNALE

ScoHcX HTFWENDIMEISKGHSFTLN-S--DAKVKIQHLQHEKFEIHLTVQNDKGE

ScoHcC HTYWQHSLLKVGQGFEFTKH-T--PAYVKVKHLQHESFTYVIDVENR-GR

ScoHcB TTFLENSIVHLDEGFSFTAR-G--HARVKVQHLQHEGFNYQIKV-NNAGG

SpiHc1 HTYYTSSTLRLSKGFDYTKD-S--EAKVIVEHTDHDDFVYVIDIDNNARV

AgiHc1 HTYYTSSTFRLSKGFDYTKD-S--EAKVIVEHTDHDDFVYVIDIDNNARV

PanHc1 HTYWTLNDFKLTKGFDYTLD-S--EAIIHMKHIDHEDFTYVFDVVNHDQK

PanHc2 RTFWQDAHLKVREGFLFTRQ-S--PAYVKLTHLDHETFTYRIDV-NNGGS

PciHc1 STYFEDFSFDLGNALDTTETVEDVKVTAHVSRLNHKPFTYKMHLHANQ--

PciHc2 STYFEDFSVDLGNALDTTETVEDVKVTAHVSRLNHKAFTYNIHINANH--

NkeHc ETYFEHFKFDLTMAVDDTPEIADVELTADVSRLNHKSFAYNFQLTNNKGS

GpuHc1 ETFFEDFEFDLKMAVDSSEAVAEVEVKAHVDRLNHKDFAYNFDIKSDS-A

GroHc1 ETFFEDFEYDLKMAVDSSEAVAEVEVKAHVDRLNHKDFAYNFDIKSDS-A

CcsHc ETFFEDYEFDLSNAVDSSDKIERVAVNASIKRLNHKDFNLKFEINSNNDV

EpuHc1 KTFFEDFEFDLQMAVDDTPEVQDVPITAIVSRLNHEPFSLTADITNNHGG

EpuHc2 KTYFEDFELGLKMAVDDTQEVQDVPITAIVSRLNHEPFSLTADIKNTHGG

OscHc1 VTYFEDFEFNLINALDDTEQIEDVPVSTTVMRLNHKPFAYHIDFTAKY--

OscHc3 VTYFEDFEFNLINALDDTEQIDDVPVSTSVHRMNHKPFDYHIIYNAKY--

OscHc2 TTFFEDFEIDLTNALDDTEEIDDVAVSTYVHRLNHKPFSYKIHVNADH--

OscHc4 TTYFEDFEIDLTHAIDDTEEIDDVAVSTYVHRLNHKPFAYKIKVKADH--

PleHc STFFEDFEFDLSNALDTTENVNEVSVKTHISRLNHKPFSLNIHAHAEH--

PleHc2 KTYFDTFEFSLVNAVDQSDKVADVAISADVHRLNHQEFSYNIDVSNNNGG

HamHcA NTFFDEFEFSLGNAVDELEKVHDVAISAYVHRLNHEEFSYKILVQNNNEG

PinHcB ITFFDEFQYSILNAVDSGENIEDVDINARVHRLNHNEFTYKITMSNNNDG

PinHcA ITFFDEFQYSLINAVDSGENIEDVEINARVHRLNHKEFTYKITMSNNNDG

PvuHc TTFFDQFKYSLVNAVDSGENVEDVEIYANVHRLNHEEFTYDIEVRNHNEE

PelHc1 TTFFDQFKYSLVNAVDSGENVEDVAIYANVHRLNHEEFTYEIEVRNHN-E

PelHc2 TTFFDQFKYSLVNAVDSGENIEDVEIYANVHRLNHEEFTYVIEVSNAN-E

PelHc3 TTFFDQFKYSLVNAVDSGENVEDVAIYANVHRLNHEEFTYDIEVRNHNEE

PelHc4 TTFFDQFKYSLVNAVDSGENVEDVAIYANVHRLNHEEFTYEIEVRNHN-E

CmaHc6 ETYFEDFEYSLINAVDDTTKVADVPISTIVKRLAHKDFTVESVITNNNEH

CmaHc1 STFFEDFEFDLRNALDTSDNVGDVTVKAHVSRLNHKPFYYNIHYNAKQ--

CmaHc2 STFFEDFKFDLRNALDTSDGVGDVTVYAHVPRLNHKPFGYNINYHAKS--

CmaHc3 ETYFETFEYSLVNAVDDTEDVDDVDIFTYISRLNHKEFSFVGDVTNELDH

CmaHc4 ETYFEDFEYSLINAVDDTAEIADVPISTVVKRLTHKSFTAKCVINNNNEH

CmaHc5 ETYFEDFEYSLINAVDDTAEIADVPISTVVKRLAHKDFTAECVINNNNEN

CsaHc ETHFEDYEYSLINGVDDTVDVLDVPISTVVSRLTHNDFKVKIGITNSQNH

PinHcC ETFFENFEYSLLNAVDDTVDIADVEILTYIERLNHKKFSFLILVTNNNNT

PvaHc1 ETYFEDFEYNLINAVDDTEQIADVDISTYVPRLNHKEFKIKVDVSNNKG-

PvaHc ETYFEDFEYSLINAVDDAEGIPDVEISTYVPRLNHKEFTFRIDVENGGA-

FchHc1 ETFFEDYEYSLINAVDDTEEIADVEISTYVPRLNHKDFAYNIEVTNNNGK

MjaHcL ETYFEDFEYNLINAVDDTEQIPDVEISTYVPRLNHKDFAFKIGVSNNKGE

MjaHcY ETYFEDFEYSLINAVDDAEGIQDVAISTYVPRLNHKEFTIKLDVKSDA--

CjaHc1 ETYWEDFEYSLINAVDDTLEVEDVEISTYVPRMAHKEFEYNIDIKNNKGS

PmaHc2 VTFFDESHINLGNMWVHTPE--KVGIEVTMKRLNHEAFKYVITATAEK--

StuHc2 ITHFNNFYIDLHNALDTTTTLGDVDIKARIGRMAHEPFKYTINVNSER--

PgrHc2 VTFFDESHINLGNMWAHTPE--KVGIEVTTKRLNHESFKYVITATADK--

CseHc2 ITYFDESHIDLNNIVENVGREQPVDIKAVVSRLNHEPFKYVITVNSKK--

PamHc2 ITYFDESHIDLNNIVDTSGQKEVVDIKALVGRLNHEPFKYVITVHSEK--

BduHc2 ITYFDESHIDLGNAVDTATHKQVVDIKAVVSRLNHEPFKYVITVNSKR--

HmeHc2 ITYFDESHIDLGNAVYTTGQREQVDIKAVVSRLNHEPFKYVITVNSKK--

TdoHc2 VTYFDEFNIDLANAVEGHYKEGDVQIKASVNRLNHEPFKYVITVHSDK--

StuHc3 KTFFEDFEFDLTQGMELTGTGDDAHIKAVMARINHKPFHYTIKVNSDV--

StuHc1 MTFFDDFEIDLTMALDDTPELADVPIKAISHRLNHAPFTYLLKMKSDA--

PmaHc1 VTYFEDFDIDMLNALDDTADLPDVDVKARVQRLNHEPFTWALHMESDK--

SamHc1 ETFFEYFDVDLLNALDDTEELPDVSIHARVRRLNHKPFVFSVRVNSEA--

BduHc1 VTYFEDFDIDLLNALDDTQELDDVEIKARVRRLNHKPFTFHITINSEL--

PamHc1 VTYFEDFDIDMLNALDDTAELQDVDIKARVRRLNHKPFSVQVTVNSER--

CseHc1 VTYFEDFDIDLLNALDDTLELKDVEIKARVRRLNHRPFTFSITVNSNQ--

HmeHc1 QTYLEDFDIDLLNALDDTQELDDVEIKARVRRLNHQPFTFRVTVSSEK--

CmoHc1 ETYFEDFDADLLNALDDTIDLDDVEIKARVRRLNHKPFTVQITAESER--

CacHc1 TTYFEDFDIDLLNALDDTVELEDVEIKARVRRLNHKPFNFKIEVNSDK--

TdoHc1 VTYFEDFDIDLLNALDDTVELPDVEIKARVRRLNHRPFTYTINLNSDK--

PgrHc1 VTYYEEFDIDMLNALDNTVDLPNVEVKARVQRLNHEPFTWALHVESDK--

ScuHc1 VTYFEDFDIDMLNALDDAAGLTDVDIKARVQRLNHKPFGIKIIANSAA--

MgeHc1 VTFFEPFDIDLYNALDDDKTVSDIEIKARVQRINHKPFTYHINVESDV--

PlaPPO EVTATVRLFMAPKFSEFRREFTYTEIRRLFFELDKFQAKIAPGWQTVTRL

AfrPPO PRRVCFRVFMTPIYDEVGRKLTFRQQTLLAVEMDKFAVTVNAPMVQLDRT

DmaPPO TQEIVFRIFLAPIADEAGRPFSFREQRLLMIELDKFVVRVTPGANMVKRK

DmePPOA1 RRTGTCRIFICPKVDERNQALNLEEQRLLAIEMDKFTVDLVPGENTIRRQ

DmePPO2 QRFGYVRIFMAPKNDERGQPMLMRDQRSMMIELDKFVTSLNPGPNTIRRR

PseHc DFEGHVRIFMSPKYTDSGNLATDEELRQHSIEMDRYSTVLSPGSNTLTRY

EpiHc1 DFTGTFRIFMAPKNDDLDIPMEINEQRILMIEMDKFQLKLSPGENNITRK

EspHc1 SKHATVRIFMAAKHDELGNEIPLDDQRRMMIELDKFKAEIPPGKSSIKRA

PimHc2 AKEGCIRIFLGPIEDELGNPLVLNTQRRFFIELDKFHHELAPGKSTISRA

PimHc3A VKRATVRIFLAPKYDELGNEIPIDDQRRLFIELDKFTATLQPGKNTVVRS

PimHc3B KKSATVRIFIAPKYDELGNEIKLENLRRLFIEMDKFKVELAPGPNTIVRN

PimHc3C DKLGTVRIFLAPVHDELGNVLNPDQLRRLMIELDKFQVDLHPGSNAIKRK

PimHc4 TK-ATIRIFLGPKCDELDNVLSLEDQRKLFIELDKFQRELKSGVNKIERN

PimHc5A AKHATVRIFLGPKYDELGNRMTLNDQRGLYIELDKFHKELAPGKNIIKRH

PimHc5B TRGAYVRIFLAPKYDELGNELTANELRRLMIEMDKFSYDLHPGRNLIERS

PimHc6 PKEATVRIFLGPKCDELHNLLEPDEQRRLFIELDKFHTTVAAGQSLIARN

AauHc6 EKHSTVRIFLAPKYDELNNKLEPDEQRRLFIELDKFFYTLTPGKNTIVRN

LpoHcII DKHATVRIFLAPKYDELGNEIKADELRRTAIELDKFKTDLHPGKNTVVRH

LpoHcIIIa TKHATVRVFLAPKYDELGNLLHPNDQRRLCIELDKFHKELKAGKNEITRN

LpoHcIIIb VKHATFRIFLGPKYDELGNLLTPEQQRRFFIELDKFHREVPSGVSVITRS

LpoHcIV VKHGTFRVFLAPKYDELGNRLILNEQRRLFIELDKFHHGIHKGHNTIVRN

LpoHcVI TKPATVRIFMAPKYDELGNELDTKNLRRLMIEMDKFHAEIHPGHNTIVRH

CroHcVI TKPATVRIFMAPKYDELGNELNTKNLRRLMIEMDKFHAEFYPGHNYIVRH

CroHcV TKPSTVRIFMAPKYDELGNELQTKDLRRLMIEMDKFHHDLHPGRNTIIRH

CroHcIV VKHGTFRIFLAPKYDELGNRLILNEQRRLFIELDKFHHGIHKGSNTIVRN

CroHcIIIb AKNATFRIFLGPKYDELGNLLTPEQQRRFFIELDKFHREVPTGKSVITRS

CroHcIIIa PKHATVRIFLAPKYDELGNLLHPNEQRRFCIELDKFHKELNAGKTEITRN

CroHcII DKHATVRIFLSPKYDELGNEIKANDLRHTAIELDKFKTDLHPGKNTVVRH

CroHcI KKDATVRIFLAPKFDELGNELPANIQRRLFIELDKFHRELISGQNIITHN

AgoHcA DKQTTVRIFLAPTYDELGNSIPLDEQRRLYIEMDKFYYTLRPGKNTIVRS

AgoHcB TRKATVRIFLGPEHDNLGNVIDIGRLRRLMIELDKFTTILEPGENVIERD

AgoHcC LRHGTCRIFLAPVHDELHNQLTPEELRRLMIELDRFRVELQPGVNQIHRH

AgoHcD AKETCVRIFLAPKYDELGNRLILEEQRRLYIELDKFHRRLEPGKNVLVRA

AgoHcE AKDVTVRIFLGPKYDELGNRLQPEQQRTLNIELDKFKATLDAGKNVVTRD

AgoHcF VKHATVRIFLGPTQDELGNRLRLNEQRRFYIELDKFHAELAAGKNTITRK

AgoHcG VKHATVRIFLGPKCDELGNILEPDEQRRLFIELDKFHKELGPGISTINRN

AgoHcX VKVATVRIFMAPRYDATGREFPFNVQRRYFIEMDKFRATLLPGENAITRN

EcaHcA DKQATVRIFLAPTYDELGNDISLDEQRRLYIEMDKFYHTLRPGKNTIVRS

NinHcA EKKGTVRIFLAPKYDELGNVIPLDEQRRLYIEMDKFDTDLRPGKNIIVRS

EcaHcB TRKATVRIFLGPEHDNLGNEFDIGRLRRLMIELDKFTTVLEPGENVIERD

NinHcB TRHAKVRIYLGPKNDHLGNPIKISNIRRLMIELDNFHVELKPGTNTVERR

EcaHcC LRHGTCRIFLAPVHDELQNQLTPEELRRLMIELDRFRVELKPGVNHNHRH

EcaHcD ARETCVRIFLAPKYDELGNRLILEEQRRLYIELDKFHRRLEPGKNVLVRA

NinHcD AKKTTVRIFLAPKYDELGNRLVLEDQRRLYIELDKFTTTLEPGRNTINRR

EcaHcE AKDVTMRIFLGPKYDELGNRLQPEQQRTLNIELDKFKATLDPGKNVVTRD

NinHcE AKQATVRIFLGPKYDELGNRLDPEHQRTLCIELDKFQVELAAGKNAITRD

EcaHcF VKHATVRIFLGPTQDELGNRLRLNEQRRFYIELDKFHAELAAGKNTITRK

NinHcF TKHATVRIFLGPVFDELGNKLSLNEARRFFIELDKFHAELAPGKNTVTRK

EcaHcG VKHATVRIFLGPKCDELGNILEPNEQRRLFIELDKFHKELGPGLNTINRN

NinHcG PKHATVRVFLAPKFDELGNRLSPDEQRPLFIELDKFHKQLAPGNNVISRN

TtrHcA KKDATVRIFLAPKLDELGNQLPANIQR-LFIELDKFHKELISGQNIITHN

EbaHcA AKEATVRIFLAPKFDELGNEIPLDEQRSLAIEMDKFRSQIQPGKSTIVRS

MgiHcA IKHGTVRIFLAPVHDELGNDLSLDEQRYLFIEMDKFKTELRPGKNTIIRG

EbaHcB TRHATVRIFLGPKYDELGNEIPPNELRHLMIELDKFHVELKPGLNTVERE

MgiHcB TKEATVRIFLAPVKDELGNDIDLHTLRRLAIEMDKFKVALQPGDNVIERK

EbaHcC TKHATVRIFLAPKYDELGNELSPNELRRLMIELDKFRAELHPGNNVIERK

MgiHcC KKHAFVRIFLAPVHDELGNVLKPEELRRLMIELDKFSVELHHGSNTIKRS

EbaHcD VKNATVRIFLAPKYDELGNRLVLDNQRGLFIELDKFHTEIPPGKSSIDRL

MgiHcD DKHATARIFLAPKYDELGNRIILDEQRRMYIEMDKFRVDLHPGKNTITRS

EbaHcE VKTATVRIFLAPKHDELGNLLEPDDQRRLAIELDKFVYELAAGKNVIAHD

MgiHcE AKEATVRMFLGPKFDELGNRLHLDEQRRLYIELDKFHVSLTPGKNSIVRD

EbaHcF VKNATVRVFLGPKYDELGNRILLNEQRRFFIELDKFRATLEPGKTIITHK

MgiHcF EKDSTVRIFLGPKYDELGNHLRIDEARRFYIELDKFHAKLSPGKNVVTRK

EbaHcG AKECIVRIFLAPQYDELGNLLTIEEGRRLFIELDKFKQTLAAGQSSFSRN

MgiHcG TKEATVRIFLGPKYDELGNVLIAEDKRRLMIELDKFHKTLAPGKNTISRN

CsaHc1 IKHATIRVFLGPKYDELGNRLAPDEQRRLMIELDKFHKELKPGKNFIHRN

CsaHc2 AKHATIRVFLGPKYDELGNRLVPDEQRRLMIELDKFHKELAPGKNVINRN

CsaHc3 AKHATLRVFLGPKYDELGNRLAPDEQRRLMIELDKFHKELAPGKNVINRN

CsaHc4 AKHATIRVFLGPKYDELGNRLAPDDQRRLMIELDKFHRELAPGKNVINRN

CsaHc5a AKHATIRVFLGPKYDELGNRLAPDDQRRLMIELDKFHKELAPGKNVINRN

CsaHc5b AKHATIRVFLGPKYDELGNRLAPDDQRRLMIELDKFHKELAPGKNVINRN

CsaHc6a AKHATLRLFLGPKYDELGNRLVPDDQRRLMIELDKFHKELAPGKNVITRN

CsaHc6b AKHATLRLFLGPKYDELGNRLVPDDQRRLMIELDKFHKELAPGKNVITRN

CsaHc6c AKHATLRLFLGPKYDELGNRFVPDDQRRLMIELDKFHKELAPGKNVITRN

HauHcB DKHAIIRLYLAPLHHNDGTEYTLTEKRTAVIELDTFLTTLKPGSNHITHD

HauHcA DKHAIIRLYLAPLHHNDGSELTLTEKRTAVIELDTFLTTLKPGHNQVTHD

ScoHcA AKKAVFRIFLAPKYNEKGELFPVNEQRQLLVELDKFVATLEPGHNVIERQ

ScoHcD EKHGVVRIFGAVINDERGHPYILNDQRHLVIELDKFTVNLKPGKNSVRQP

ScoHcX DTDLFVRIFLLPLEDEESHELSLEEMVRMAVDIEKRVIPAKPGSNDIVIS

ScoHcC TRTGFFRIFAAPKYNELGQKWHINDQRLIMVEMDKFIEKLYPGKNTIERH

ScoHcB EHKVVFRVFLAPKYDEEHHEFDFNEQRGMAIELDKFVATVPAGSSTVEQH

SpiHc1 EKTAVLRIFLAPKYDERGHPLTLKEQRVMMIELDKFKATLKPGHNVVRRC

AgiHc1 EKTAVLRIFLAPKYDERGHPLTLKEQRVMMIELDKFKAPLKPGHNVVRRC

PanHc1 EKHAAIRVFLRPTYDEAGHEFTPEQFWPLIIELDKFTYTLKPGHNIIRHN

PanHc2 AHEAVVRIFLAPVYDEFEHRFDIKHQKSLMIQMDKFVTKLTPGKNTIVRS

PciHc1 AEKVTVRVFIAPKYDQNLAPLEINQARWFAIALDTFWVDVAAGDSVIERS

PciHc2 AEKVTVRVLIAPKYDENHVPLDINHARWAGIELDTFWVDVPAGDSVIERS

NkeHc PASAVFRVFLCPRKDYNGILIPLEERRWLCIEMDKFWKTLSPGGNTVVRK

GpuHc1 DQHAVVRVFLCPRRDNNGIQFTFDEGRWNCIEMDKFWTKLAAGDNHIKRK

GroHc1 DQHAVVRVFLCPRRDNNGIQFTFDEGRWNCIEMDKFWTKLAAGDNHIKRK

CcsHc EKHGVVRTFICPRKDAHGVIFSFEEGRWNCIELDKFWTKLAPGVNSITRS

EpuHc1 DVFATIRVFLCPRYDANEILFDLNEGRWHCIEIDKFWRTLHSGDNHLERK

EpuHc2 DVFATIRVFLCPRHDHNGILFTLEEGRWHCIEIDKFWRTLHAGDNHIERE

OscHc1 NAIATVRIFLCPKHDYNHIEMTMDEYRWGCLEMDKFWIDAHYGENNLVRH

OscHc3 DAVATVRIFLCPKHDYNHIELKMDEYRWGCLEMDKFWVDAHSGENHIVRH

OscHc2 EDTATVRIYLCPKHDYNGIELHLDEYRWGCIEMDKFWTHLAAGSQDIVRK

OscHc4 EDTATVRIFLCPKHDYNGIELHLDEYRWGCIEMDKFWTHLSAGSQEITRK

PleHc DDKVTVRVYISAKHDENHIALDIDESRWGAILLDTFWTEVHAGDNEIKRK

PleHc2 KVLGTIRIFLCPVKDSNGVTFTWEEGHWHCIEMDKFYKSLAPGINHIVRK

HamHcA DILATVRIYLCPTVDNNHIHIPLSEGHWTCIEMDKFYKQLKTGSNKIDRK

PinHcB ERLATFRIFLCPIEDNNGITLTLDEARWFCIELDKFFQKVPSGPETIERS

PinHcA ERLATFRIFLCPIEDNNGITLTLDEARWFCIELDKFFQKVPKGPETIERS

PvuHc DKFATVRIDLCPTEDNNGITLNLDEARWLCLELDKFWTKLGDGKNLIERS

PelHc1 EKFATVRIFLCPTEDNNGITLNLDEERWLCLEMDKFWRKLANGKNLIERS

PelHc2 VHLATVRIFLCPTEDNNGITLNLDEERWLCLELDKFWTKLANGKNLIERS

PelHc3 EKFATVRIFLCPTEDNNGITLNLDEARWLCLEMDKFWKKLGNGLNMIERS

PelHc4 EKFATVRIFLCPTEDNNGITLNLDEARWLCLEMDKFWKKLANGKNLIERS

CmaHc6 EVMATVRIFAWPMYDNNDVEFSFNDGRWNAIEMDKFWIKLASGTNTITRS

CmaHc1 DDKVTVRVYLTPVRDENGIKLDINDNRWSAIMIDTFWTEVKAGTHNHRRS

CmaHc2 AERVTVRVYLTPKYDENGVQREMEVGRWHAILLDTFWVDVEAGDHVYHRD

CmaHc3 DVLATVRIFAWPHEDNNGVAFSFNDGRWNAIEMDKFWVMLHPGHNHIERS

CmaHc4 DVIATVRIFAWPKYDNNHVEFSFNDGRWNAIEMDKYWTKLTPGPNTITRS

CmaHc5 EVMATVRIFAWPKYDNNHVEFSFNDGRWNAIEMDKFWTKLAPGPNTITRS

CsaHc EVLATVRIFAWPKYDNNHVEFSFNEGRWNAIELAKFWTKLNSGSNIIERS

PinHcC EVLATVRIFAWPLRDNNGIEYSFNEGRWRALELDRFWVKVKHGHHQITRQ

PvaHc1 EEVSYRHIFAWPHLDNNGIKFTFDEGRWNAIELDKFWVKLPGGTHHIERK

PvaHc ERLATVRIFAWPHKDNNGIEYTFDEGRWNAIELDKFWVSLKGGKTSIERK

FchHc1 EVLTTVRIFAWPHRDNNGIEYTFDEGRWNAIELDKFWVKLSPGSNHIVRK

MjaHcL ETLATVRIFAWPHLDNNGIEFSFDEGRWHAIELDKFWVKLGTGVTEITRK

MjaHcY ARLATVRIFAWPHKDNNGIEYTFDEGRWNAIELDKFWVSLSSGSNAIERK

CjaHc1 KSLATIRIFGWPHRDANGEPLSFDEGRWVALELDKFWVELNPGNNHIVRK

PmaHc2 ETEGIVRIFLSPTYNWFGQEITLQDGHWGAIEMDRFPVKLTAGENVITRS

StuHc2 PVTATVRIFLAPAYNWYGEEIHLDEGRWLAVELDKFAVKLHEGENVITRL

PgrHc2 DTEGIVRIFLSPTYNWFGQEITLQDGHWGVIEMDRFPVKLTAGENVITRS

CseHc2 PVRGIVRIFLAPKLNWFGQRVPLDVARWGFIELDRFPVRLASGDNIVSRN

PamHc2 PVRAIVRVFPLPKYNWFGQMVPLDEARWGAIELDRFPVSLVHGDNIVTRN

BduHc2 PVNAIVRIFLAPKYDWFGQKVPLDAARWGVIELDRFPVKLVNGDTVITRS

HmeHc2 AVHGVARIFLSPKYNWFGQKIPLHVARWGMIELDRFPVKLEAGDNVITRN

TdoHc2 VAEGIVRIFIAPKYDWFGEVVPLDKIRWSLIELDRFPVHLRQGDNVIVRS

StuHc3 KRTGMVRIFLAPKYDWYGEEIDITHNAWQTVELDKFLVPLDPGENVIVRK

StuHc1 SHVATVRIFLGPKYNSYGEELSLDTKRWMMVEMDKFVVHLHSGNSEILRK

PmaHc1 EVTAAFRVFLGPKKDWYESDFTINEVRPYLIEIDKFVTKVVAGKSVIHRK

SamHc1 ERFVTVRVFLGPKYDWFGQEIPINEKRHYIVEIDKFVAKVNAGKTVIARK

BduHc1 DATAAVRVFLGPKYDWFGQEIPINEKRLYMVEIDKFVAKVNKGTTVVQRK

PamHc1 DAMAVVRIFLAPKYDWFGQEIPINEKRLYMIEIDKFVTKVNKGTTVVQRK

CseHc1 EQMAAVRIFLGPKFDWFGQEIPINEKRLYVIELDKFVAKVNKGATVIQRK

HmeHc1 NANVAVRVFLGPKYDWFGVQIPLNEKRLYMVEIDKFYTTVNKGTTVIERK

CmoHc1 ETLATVRIFLGPKYDWFGQEIPLEEKRQYLVEIDKFVTKVNSGKTTIQRK

CacHc1 EYTAAVRVYIGPKYDWFGQEITLDEKRLYMVEIDKFVTKLTAGHNNIFRK

TdoHc1 DATVIVRVFLGPKYDWFGQEIPLEKKRLYMIELDKFVAKVSAGQTPIQRK

PgrHc1 EVTAAFRVFLGPKQDWYGSDFTINTVRPYVIEIDKFVAKVVAGKSVIHRK

ScuHc1 EKTVTVRLFLAPKYDWYGREVPLDIQRWKFIELDKFAVKLTAGENAIVRK

MgeHc1 ERKVVVRTFIGPKYDWYHQEVPVNEKRWEMVELDKFLTHIPAGKSVIERL

PlaPPO SDESNVTIPDYTNFTWFETNPELGTRPGGFRET-----------------

AfrPPO SRESSVTIPIERFFRVYERRTANT---------------SD----ALSNY

DmaPPO SDDSSVTIPFERTFRDLEAAVPATPLAPGGVPTD---LPTD----RMTN-

DmePPOA1 STESSVAIPFERSFRPVGADYQPK---------------AA----DELAR

DmePPO2 STESSVTIPFERTFRNLDANRPAAGT-------------------PEELE

PseHc SQESAVTMTPNRQWTQLSSVD------------------VEFNPSSDNEN

EpiHc1 SEDSNVTLHKEMTW-EMIENQPDT---------------------SGPSS

EspHc1 SKDSSVIVKKDLSIAQLRAGETGG---------------AN----K---E

PimHc2 SKDSSVTVTHVPTVEEIQKGEHGD---------------DD----N---E

PimHc3A SVDSSVTLSQQHTFEELEKGE-GL---------------DE----S-RTE

PimHc3B SVDSSVALSRDHTVEELERGD-GI---------------TE----D-RTE

PimHc3C SVDSSVTLSYETTFEKLLRGE-LV---------------DA----R-PTE

PimHc4 AIESSVTVSELHTFQDLQQGK-GV---------------SE----D-ATE

PimHc5A SKDSSVTITHIPTFAELEAGK-GV---------------SE----D-NSE

PimHc5B SHDSSVIVHKEKTFAELVSHH------------------DE----H-AGE

PimHc6 HKESSVTISKIRTFNELKGGA-GL----------------D----H-ANE

AauHc6 HQDSSVTISKVRTFDQLGAGE-GV---------------SE----D-STE

LpoHcII SLDSSVTLSHQPTFEDLLHGV-GL---------------NE----H-KSE

LpoHcIIIa SVDSSVTISTLHTFDELESGV-GV---------------NE----N-ADE

LpoHcIIIb STDSSVTISHVPTFEELKDGK-GV---------------NL----D-NSE

LpoHcIV STESSVTVSKIHTFNELKAGV-GV---------------DE----K-NTE

LpoHcVI SKDSSVTISSARSFGQLAHGE-GI---------------NE----H-ANE

CroHcVI SKNSSVTISSARSFGQLAHGE-GV---------------NE----H-ANE

CroHcV SKDSSVTLSSERTFDELAHGE-GI---------------NE----H-ANE

CroHcIV STESSVTISKVHTFNELKAGV-GV---------------KE----S-NTE

CroHcIIIb SADSSVTISHVPTFEELRNGK-GV---------------DV----N-NSE

CroHcIIIa SVDSSVTISTLRTFNELNDGV-GV---------------SE----N-ADE

CroHcII SLDSSVTLSHQPTFEDLLHGI-GL---------------ND----N-KSE

CroHcI AADSSVTVSTLRSFEDLKAGK-GV---------------SE----D-ATE

AgoHcA STDSSVTLSSVHTFKELLRGE-DL---------------VE----G-QTE

AgoHcB SIDSSVTIREQYTYRQLQTGR--S---------------NR----E-QSE

AgoHcC SRDSSVTISKQAKFGELLKGH-GT---------------NV----N-ANE

AgoHcD SGDSSVTLSKVPTFQELESGNANV---------------SP----N---E

AgoHcE HRNSTVTVEQSIPVKRLRDEG-GV-----------------------AGE

AgoHcF SADSSVTVSHTPTFEQLQRGE-GV---------------DE----N-TTE

AgoHcG SVESNVTVAHTYTFDELREGK-LA---------------PE----D-ATE

AgoHcX STDSAVTVAHIPSFKELKEKA-VI---------------SDKQDRNITDT

EcaHcA STDSSVTLSSVHTFKELLRGE-DL---------------VE----G-QTE

NinHcA STDSTVTISSTYTFKELLHGE-DL---------------EE----D-RSE

EcaHcB SIDSSVTIREQYTYRQLQDGR--S---------------NR----E-QTE

NinHcB SIDSSVTLSKQPTLKELLQGK-GT---------------DR----R-GDE

EcaHcC SRDSSVTISKQPKFDELLKGK-GT---------------N-----N-ANE

EcaHcD SGDSSVTLSKVPTFEELESGNANV---------------NP----N---E

NinHcD SVDSSVTLSHVPTFEELEKGE-NI----------------P----NRETE

EcaHcE HRNSTVTVEQSVPVKKLREEG-GV-----------------------AGE

NinHcE HKLSSVTVSETHTFKQLLAGE-GV---------------SE----N-TTE

EcaHcF SSESSVTVSHTPTFEELQRGE-GV---------------DE----N-TTE

NinHcF STESSVTVKPTPKFSQLQSGE-GI---------------SE----N-STE

EcaHcG SVESNVTVAHTYTFDELREGK-LA---------------PE----D-ATE

NinHcG AIDSNVTLSHTYTFDELRAGQ-SA---------------SA----D-ASE

TtrHcA AADSSVTVSTLRTFEDLKAGK-GV---------------SE----D-ATE

EbaHcA SSDSSVTLTHELTYRQLLHGE-GI---------------SE----H-RTE

MgiHcA SFDSSITLSRQNTFAKLEHGS-DI---------------DE----N-LTE

EbaHcB SSESSVTIGTRPSLSKLASGE------------------------S-SHE

MgiHcB SSESSVTIKDPKSLDELARGE-LA---------------DE----H-EKE

EbaHcC SSESSVTLSTERSFGELLHGE-GA---------------ND----N-ADE

MgiHcC SKDSSVTISSVRSFRELLEGE-GI---------------KE----H-ASE

EbaHcD SSESSVTLSHTPTFDELQRGE-GV---------------DD----D-KTE

MgiHcD SDKSSVTITHVPTFDELEAGI-GV---------------GE----N-DSE

EbaHcE HRDSSVTVSKIYTFSQIQAGE-GV---------------HE----D-GSE

MgiHcE HRDSNVTISKLRTVAQLREDS-G-----------------E----D-DHY

EbaHcF SSDSAVTIDRTPTFEDLQKGD-VV---------------NE----T-NSE

MgiHcF SSESNVVANKTPNFDELEGGA------------------------S-SDQ

EbaHcG AIDSNVTLSKVPTFDDLKSGE--V---------------SD----D-YSE

MgiHcG SVDSSVTVSHVPTFDELKSG--GV---------------AA----N-NQE

CsaHc1 AFDSNVTLSHTYTFDELRSGL-SA---------------SQ----D-ATE

CsaHc2 AIESSVTLAHTYTFDELKAGQ-GA---------------SE----D-SSE

CsaHc3 AAESNVTLSHTYTFDELRSGE-GG---------------PA----D-ANE

CsaHc4 AIESSVTLAHTYTFEELKAGQ-AA---------------SQ----D-ATE

CsaHc5a AIESSVTLSHTYTFDELKSGQ-GA---------------NE----N-STE

CsaHc5b AIESSVTLSHTYTFDELKSGQ-GA---------------NE----N-STE

CsaHc6a AAESNVTLSHTYTFDELKAGK-GG---------------AE----N-ATE

CsaHc6b AAESNVTLSHTYTFDELKAGK-GG---------------AE----N-ATE

CsaHc6c AAESNVTLSHTYTFDELKAGK-GG---------------AE----N-ATE

HauHcB STDSAVTYFHE----------------------------------KDQHH

HauHcA STDSAVTYFHE----------------------------------KDQHH

ScoHcA SKESSVTMSKDHVFGEIRN--------------------------LADDH

ScoHcD CYNSAVTAKYDVFYGDVES--------------------------QKPQE

ScoHcX S--RSIGAPANKFFGSFEE--------------------------RYISE

ScoHcC SEDSTVTMSSASIFSDISS--------------------------EQSED

ScoHcB SSKSSVTQSNDNFYGSSAT--------------------------RSSEN

SpiHc1 SNESSVTMPVDHIYGDITR--------------------------TVDED

AgiHc1 SNESSVTMPVDHIYGDITQ--------------------------TVDED

PanHc1 VNESSVTMPREQIYDSVKQWS------------------------TKEHD

PanHc2 SLNSTVTMEANSIFGAKRP--------------------------SKTID

PciHc1 SSDSNVAIPDRISFPTLIHDTDEAVAAGGQLSFSVDGHNTR---------

PciHc2 SSDSNVAISDRISFPTLIHDTDEAVASGSSLSFATD-HHAR---------

NkeHc SGESSVTIPDRKHFAEMMSETDAAASSGGSLDYS---DLAH---------

GpuHc1 SSESSVSVPDVPSFASLIHDADAAVASGSELHLE---DFDR---------

GroHc1 SSESSVTVPDVPSFASLIHDADAAVASGSDLHLE---EFDR---------

CcsHc SKDSSVTVPDVPSFKRLIADTKAALAEDKEVKFE---EYHR---------

EpuHc1 SSDASTTVPDIPSYESLMKMADAAVASGEELHIE---QFTR---------

EpuHc2 SSDASTTVPDIPSFHTLIEKADAAVAAGEELHLE---QFTR---------

OscHc1 SHESSVTIPDRIPFDDLIKQADDAVANDSEFPR----RDWR---------

OscHc3 AHESSVTIPDRIPFEDLIKQADEAVANDSEFPR----RDWR---------

OscHc2 ASESSVTIPDRVPFSELIKEADEAVAAGSDLEH----TDIR---------

OscHc4 GSESSVTIPDRVPFSDLIKEADDAVAAGSDLDH----KNIR---------

PleHc SSESSVAIPDRVSFPQLIHDADEAVANGAELPH----KESR---------

PleHc2 STDSSVTVPDRPSFNILKSKTDRAVSSHSKLDLH---KFER---------

HamHcA STESSVTVPDSLSLAELHEKTDQAVASGSKLDLH---EYER---------

PinHcB SKDSSVTVPDMPSFQSLKEQADNAVNGGHDLDLS---AYER---------

PinHcA SKDSSVTVPDMPSFQSLKEQADNAVNGGHDLDLS---AYER---------

PvuHc SKDSSVTVPDMPSFESLKKQADEAVNGGHDLDLS---AYER---------

PelHc1 SKDSSVTVPDMPSFESLKHQADEAVKNGEDLDLS---AYER---------

PelHc2 SKDSSVTVPDMPSFESLKHQADEAVKNGEDLDLS---AYER---------

PelHc3 SKDSSVTVPDMPSFESLKHQADEAVKNGEDLDLS---AYER---------

PelHc4 SKDSSVTVPDMPSFESLKHQADEAVKNGEDLDLS---AYER---------

CmaHc6 SKDSSITVPDVPSFQDLIEKTKEAVSSGSELHLE---EYVS---------

CmaHc1 GFESNVAIPDRISFEELIKETDEAVDNNLELSL----NSGR---------

CmaHc2 ASQSNVAIPDRISFEELIKETDEAVANNEELSL----NSGR---------

CmaHc3 SHDSSATVPDIPSFQFIKDRTNEAIAQNKELHIE---EFES---------

CmaHc4 SMDSSVTVPDVPSFLTLIEKTEEAVSSGSELHLE---DYVS---------

CmaHc5 SKDSSVTVPDVPSFLTLIEKTEEAVSSGSELHLE---DYVS---------

CsaHc SKDFGVTVPDVPSFQTLMDMTDEALGSGSALHLE---DYES---------

PinHcC STESSVTVPDVPSLQTLIDRADAAISSGCALHLE---DYES---------

PvaHc1 CSESAVTVPDVPSFATLFEKTKEAL-GGADSGLK---DFES---------

PvaHc STESSVTVPDVPSIHDLFAEAEA---GGA--GLA---KFES---------

FchHc1 SSESAVTVPDVPSFDTLFKKAEAAL-GGGDAGLT---EFES---------

MjaHcL CSESAVTVPDVPSFATLFEKTKAAL-GGADSGLT---DFES---------

MjaHcY STESGVTVPDVPSIQTLFDKAAA---GGA--GLT---EYES---------

CjaHc1 SSDSGVTVPDVPSFKDIMDRTEAALKGGGNLDLH---EYES---------

PmaHc2 GKKSVVTIDEPMSFAEIHK----AVADKDATHFH---KEFR---------

StuHc2 SKDSTITIPDMKSHKDMIREVESALAGELEYHID---EHHR---------

PgrHc2 GKKSVVTIDEPMSFAEIHK----AVADKDATHFH---KEFR---------

CseHc2 SVDSIITVPEPRSFSNLVSDVEKAIRGEQVFSYD---KYYR---------

PamHc2 SEDSTVTIPEPRSFPDLMSDVQKAIRGEQDFKYD---IHHR---------

BduHc2 SDQSTVTIPEPKSYPELVNDVERAVRGEVDFVYD---KHHR---------

HmeHc2 SEDSIVTIPEPRSFPHLINYVERAINGEEEYVVD---KYHR---------

TdoHc2 SQDSTVTIPEPRSYPELIKEIEDALNGQDEYAID---KYHR---------

StuHc3 SDESTVTIPDPPSYAQLVKEVEDALSGTSVLKVH---KFHR---------

StuHc1 STESSVTIPDPKGYKGMVEAVKSAIAGDSEFKVN---KEHR---------

PmaHc1 SSESSVTIPDRETTKVLLEKVEHALEGKETLNVN---KDER---------

SamHc1 SSESSVTIPDRETTKVLTQRVEDAIAGKVQLTVN---KDVR---------

BduHc1 SSESSVTIPDRETTKDLVKKVHDALEGKSSFYVC---KDVR---------

PamHc1 SSESSVTIPDRETTKVLTKRVEEALQGKTTYMVN---KDVR---------

CseHc1 SSESSVAIPDRETTKILVRRVDDALQGKATYTVN---KDVR---------

HmeHc1 SSESSVTIPDRETTKVLQKKVEDAIQGNGAFLVN---KDVR---------

CmoHc1 SSESSVTIPDRETTKLLVAKVENAINGKATINVN---KDVR---------

CacHc1 SSESSVTIPDRETTKVLHKKVKDALKNSTPLLVN---KDVR---------

TdoHc1 SSESSVTIPDRETTKVLVQRVKDALEDKTTWNVN---KDLR---------

PgrHc1 SSESSVTIPDRETTTLLLDRVDLALDGKETLNVN---KDER---------

ScuHc1 SSESSVTIPDPVSTHDLRKLVDDAIAGTATLEVD---KDVR---------

MgeHc1 STESTVTTPDYESFRSLVNRVDEALKGNKEFIID---EEFR---------

PlaPPO --FC----GCGWPNHMLLPKGKPE-GMKFILFAMLTDWNQDKVNGAT-KQ

AfrPPO EMFC----GCGWPEHMLVPKGSAE-GMQFELIVIATDWSKDEVPVKT-TN

DmaPPO --FC----GCGWPDHFLVPRGAPNPGMPFTLFVMATSWKEDRVVNGEDSK

DmePPOA1 FKFC----GCGWPQHLLLPKGNAQ-GMLFDLFVMISDYSQDSVEQPKTPN

DmePPO2 FNFC----GCGWPNHMLVPKGLPE-GLQCVLFIMVSNYENDRIDQQL---

PseHc --FC----GCGWPDHLLIPKGTRN-GIKYVLFVMISNWRKEKIGETE-TY

EpiHc1 QHYC----ACGWPHHLLIPKGSAQ-GTAFHCFIMITDWKTDEVKSSK---

EspHc1 --TC----SCGWPEHLLVPRGNHR-GMVFDLVVILTDWEEDKATDTA-SK

PimHc2 --YC----SCGWPQHLLVPRGTHR-GMKFCLFVMVTDWEHDKAGELA-HK

PimHc3A --FC----SCGWPQHLLVPKGDIK-GMEFHLFVMLTDREKDKVSDGD-SH

PimHc3B --YC----SCGWPQHLLVPKGNSK-GMTFYLFVMLTDRDADKVADDDPEH

PimHc3C --YC----SCGWPEHLLLPKGNEK-GMEFHLFVMLTDWLDDVVGESR-KE

PimHc4 --FC----SCGWPEYMLIPRGNSK-GLQFQLFVMITDGVQDTAGSAS-DK

PimHc5A --FC----SCGWPQHMLVPRGNER-GMIFQLFVMLTDYTHDTVSGAS-EK

PimHc5B --SC----NCGWPEHLLVPKGHVN-GMPFHLFVILTDLAHDLVYDHGHRP

PimHc6 --YC----SCGWPEYMLVPRGHHR-GMVFQLFVMLTDYEQDKIADIN-EN

AauHc6 --YC----SCGWPEHMLIPRGSHK-GMEFELFVMLTDHDEDTVAGLS-EN

LpoHcII --YC----SCGWPSHLLVPKGNIK-GMEYHLFVMLTDWDKDKVDGSE---

LpoHcIIIa --YC----SCGWPKNMLVPRGNNK-GMTFELFVMLTDWEHDNVGGKGSDN

LpoHcIIIb --FC----SCGWPQHMLIPRGNHL-GMEFQLFVMLTDWEQDQAGKDL-EH

LpoHcIV --YC----SCGWPQHMLVPRGNEK-GMDFQLFVMVTDWEGDHVNGEP-S-

LpoHcVI --YC----SCGWPDHLLVPRGDEK-GMPFHLFVMLTDWLHDQVGDHG-KT

CroHcVI --YC----SCGWPDHLLVPRGNEN-GMPFHLFVMLTDWLQDQVGDHG-KT

CroHcV --YC----SCGWPDHLLVPRGNEK-GMPFHSFVMLTDWLHDQVGDHG-KT

CroHcIV --YC----SCGWPQHMLVPRGNEK-GMDFQLFVMVTDWEDDNVNGAL-S-

CroHcIIIb --FC----SCGWPQHMLIPRGNYL-GMEFQLFVMLTDWEQDKAGKDL-EH

CroHcIIIa --YC----SCGWPKNMLIPRGNHK-GMVFELFVMLTDWEHDNVSGKGQES

CroHcII --FC----SCGWPSHPLVPKGNVK-GMEFHLFVMLTDWDKDKVDGSE---

CroHcI --YC----SCGWPQNMLIPRGNRK-GMVFELFVMLTDWEKDNVNGSV-G-

AgoHcA --FC----SCGWPQHLLVPKGSEK-GMQFDLFVMLTDASVDRVQSGD-GT

AgoHcB --YC----SCGWPNDLLVPKGNEH-GMKFRLFVMLTDAVQGQVGDHG-AT

AgoHcC --YC----SCGWPDHLLVPKGNDR-GMPFHLGVIITDYLYDLVGDYG-HD

AgoHcD --YC----SCGWPEHMLVPRGKDR-GMDFYLFVVLTDYEEDKVQGAG-EQ

AgoHcE --YC----SCGWPEHMLIPKGNHR-GMDFELFVILTDYAEDAVNGHG-EN

AgoHcF --FC----SCGWPEHLLVPRGTYK-GMDFQLFVMLTDYEEDHVGSHN-GQ

AgoHcG --YC----NCGWPKHMLIPKGTHK-GMEFQLFVMLTDYTADNPDGGA-GK

AgoHcX --YC----SCGWPQHFLVPRGTQR-GMEFKLFVMLTDHRDDAVTSTVQAE

EcaHcA --FC----SCGWPQHLLVPKGNEK-GMQFDLFVMLTDASVDRVQSGD-GT

NinHcA --FC----SCGWPQHLLVPKGSNK-GMVFDLFVMITDAEKDKVPTSG-KK

EcaHcB --YC----SCGWPNDLLVPKGNEH-GMKFRLFVMLTDAVQGQVGDHG-AT

NinHcB --YC----SCGWPDHLLIPKGDSS-GMKFHLFAIFTNYFEDTVNDHG-RT

EcaHcC --YC----SCGWPDHLLVPKGNDR-GMPFHLCVMITDYLYDLVGDYG-YD

EcaHcD --YC----SCGWPEHMLVPRGKER-GMDFYLFVMLTDYEEDKVQGAG-EQ

NinHcD --YC----SCGWPEHMLVPRGTPR-GMVFHLFVMLTDYVEDKVLDDS-AT

EcaHcE --YC----SCGWPEHMLIPKGNHR-GMDFELFVIVTDYAQDAVNGHG-EN

NinHcE --FC----SCGWPEHMLIPRGSHK-GSEFDLFVILTDYEQDAVGGEA--S

EcaHcF --FC----SCGWPEHLLVPRGTYK-GMDFQLFVMLTDYEDDHVGSHN-GQ

NinHcF --FC----SCGWPEHLLVPRGTHK-GMDFYLFVMLTDYEHDHVNGLN-EK

EcaHcG --YC----NCGWPRHMLIPKGTHR-GMEFQLFVMLTDYTVDNPCGGA-GK

NinHcG --YC----SCGWPEHMLVPRGTHK-GMDFQLFVMLTDYSEDNPEGAN-VK

TtrHcA --YC----SCGWPQNMLIPRGNRK-GMVFELFVMLTDWQKDNVDGGG-G-

EbaHcA --YC----SCGWPQHLLVPRGNEK-GMVFHLFVMLTDWSQDKVSDGS-AE

MgiHcA --YC----SCGWPSHLLVPRGNEK-GMDFDLFVMVTDWEKDKVESAH-DD

EbaHcB --YC----SCGWPNHLLVPKGKEL-GMQYHLFVMLTDYNIDLVGDHG-KT

MgiHcB --FC----SCGWPDHLLVPKGDEQ-GMKFHLFVMLTDRRMDAVGNHG-KT

EbaHcC --FC----SCGWPQHLLIPKGNSR-GMEFHLFVMVTDWLQDTVTSGH-SQ

MgiHcC --FC----SCGWPDHLLIPKGNDQ-GMEFNLFVMVTDELRDRVGDYG-HA

EbaHcD --FC----SCGWPEHMLIPRGNSR-GMAFQLFVMLTDWEHDNVGGQG-GT

MgiHcD --FC----SCGWPSHMLVPRGTTR-GMKFQLFVMLTDFEKDDVGGSS-QA

EbaHcE --SC----SCGWPEHMLLPRGNFK-GMEFHLFVILTDHDQDAVEGAT-TK

MgiHcE --YC----SCGWPEHMLIPRGNHR-GMEFDLFVMLTDYTADSALDST-PE

EbaHcF --FC----SCGWPQHMLIPRGNHR-GMDFQLFVMLTDWDEDHVGPKT-DA

MgiHcF --FC----SCGWPNYMLLPRGNHK-GMEFELFVMLTDWGEDHVGSLS-DH

EbaHcG --FC----SCGWPENMLIPRGNHK-GKPFVLFVLLTDFEKDKV--PD-FQ

MgiHcG --YC----SCGWPGHMLIPRGTRK-GMDFHLYVVLTDWEKDKVNGGD--K

CsaHc1 --YC----SCGWPEHMLIPRGTTK-GIEFELFVMATDYTHDNPEGAD-VK

CsaHc2 --YC----SCGWPENMLIPKGTHK-GMEFELFVMATDYTLDNPEGAS-VK

CsaHc3 --YC----SCGWPEHMLLPKGTHK-GMEYELFVMATDYTQDNPDGAN-VK

CsaHc4 --YC----SCGWPENMLVPKGTSK-GMDFELFVMCTDYTVDNPEGAN-VR

CsaHc5a --YC----SCGWPENMLVPRGTHK-GMEFQLFVMATDYTKDNPEGAS-VK

CsaHc5b --YC----SCGWPENMLVPRGTHK-GMEFQLFVMATDYTKDNPEGAS-VK

CsaHc6a --YC----SCGWPEHMLLPRGTHK-GMEYELFVMATDYTKDNPDGAN-VQ

CsaHc6b --YC----SCGWPEHMLLPRGTHK-GMEYELFVMATDYTKDNPDGAN-VQ

CsaHc6c --YC----SCGWPEHMLLPRGTHK-GMEYELFVMATDYTKDNPDGAN-VQ

HauHcB --DC----PCGWPQNLLLPKSNYQ-GMEWRLFAVVDDADEEHSEIGE--N

HauHcA --DC----PCGWPQNLLLPKSNYH-GMEWRLFAVVDDADEEHSEIGE--N

ScoHcA --QC----SCGWPDYLLLPKGKYE-GMTYQLFVVATDYEEDHVEDAG-EE

ScoHcD --GC----NCGWPDYMLLPKGKYE-GLRFRVFAIVTNHDEDKVSDQE--T

ScoHcX --DCNFHSHCGWPNYLLVPKGSSQ-GTPFAFVVMLTLAEDDFTPNMD-DT

ScoHcC --HC----SCGWPDYLLVPKGNFE-GFPMEVFVIVTDYEEDKVEGPD-EG

ScoHcB --HC----SCGWPDYLLIPKGNHQ-GVQFNVYVIATSYDEDHVESDE--S

SpiHc1 --HC----HCGWPEYLLVPQGDYD-GMHYELFVMATNYDEDKAQDTD-LS

AgiHc1 --HC----HCGWPEYLLVPQGDYD-GMHYELFVMATNYDEDKDQNAD-MS

PanHc1 --HC----HCGWPEYMLFPKGNYE-GMAFKLFVMVTDWDQDKISDDT--S

PanHc2 --NC----RCGWPDYLLVPKGNYE-GMKFQLFVMVGDWAKDENTESR-GN

PciHc1 --------GCGHPQRLLLPKGTTN-GMDFHFDVYISSGE-DSIHDHV---

PciHc2 --------GCGHPQRLLLPKGTTN-GMDFHFDVYISSGE-DSVHDHV---

NkeHc --------SCGQPERLLLPKGKVE-GMEFGLLIAVTDGEHDAAVSGL--E

GpuHc1 --------SCGLPQRMLLPKGTEE-GLDFLLVVAVSDGTTDAQHDAL--E

GroHc1 --------ACGLPQRMLLPKGTEE-GLEFLLVVAVSDGTTDAQHDAL--E

CcsHc --------SCGIPNRLLLPKGSSE-GMKFVLAVAVTEASSDAHPEIL---

EpuHc1 --------SCGIPNRLLIPKGTPE-GMEFALFVAVTDGSKDAAIEGL--E

EpuHc2 --------SCGIPNRMLLPKGTPE-GMEFALFVAVTDGSKDAAIEGL--E

OscHc1 --------ACGHPARLLLPKGKTK-GMEFFLFVAITSGD-DAALSKP--T

OscHc3 --------ACGHPARLLLPKGKSK-GMEFVLFVAITSGD-DAALSKP--T

OscHc2 --------ACGHPARMLLPKGKTE-GMEFNLFVAITSGD-DAAHPDP--A

OscHc4 --------ACGHPARMLLPKGKSE-GMEFNLFVAITSGD-DAAHPDL--A

PleHc --------SCGHPQRLLLPKGKEQ-GMDFWLDIIITSGD-DAVQDDL--T

PleHc2 --------SCGIPNRLLLPKGKAR-GMDFALFVGVTNGDEAKTVDDP---

HamHcA --------SCGLPARLLLPKGTVN-GMDFDLFVAVTNGDEDKVTDHP--E

PinHcB --------SCGIPDRMLLPKSKPE-GMKFNLYVAVTDGDKDTEGHNG---

PinHcA --------SCGIPDRMLLPKSKPE-GMEFNLYVAVTDGDKDTEGHNG---

PvuHc --------SCGIPDRMLLPKSKPQ-GMEFNLYVAVTDGDKDTDGSHG---

PelHc1 --------SCGIPDRMLLPKSKPQ-GMEFNLYVVVTDGDKDTDGSDG---

PelHc2 --------SCGIPDRMLLPKSKPQ-GMEFNLYVAVTDGDKDTDGSDG---

PelHc3 --------SCGIPDRMLLPKSKPQ-GMEFNLYVAVTDGDKDTDGSDG---

PelHc4 --------SCGIPDRMLLPKSKPQ-GMEFNLYVAVTDGDKDTDGSDG---

CmaHc6 --------GLGLPNRFLLPKGTTQ-GMKFHLVVFVSDGAKDAAIDGL---

CmaHc1 --------SCGHPQRLLLPKGNEE-GLQFWLNVYVTSGE-DAVHDDL---

CmaHc2 --------SCGHPQRLLLPKGNEE-GLACWFTVYVTSGG-DGVHDDL--Q

CmaHc3 --------GLGLPNRFLIPKGNVK-GLDMDVMVAITSGEADAAVEGL---

CmaHc4 --------GLGLPNRFLLSKGTTK-GMDFHLVVFVSDGAKDAAIDGL---

CmaHc5 --------GLGLPNRFLLSKGTTE-GMKFHLVVFVSDGAKDAAIDGL---

CsaHc --------GLGLPDRFLLPKGKTE-GMDFHVVVFVSDGAKDAAVDGL---

PinHcC --------ALGLPNRFLLPKGQAQ-GMEFNLVVAVTDGRTDAALDDL---

PvaHc1 --------ATGIPNRFLIPKGNEQ-GLEFDLVVAVTDGAADAAVDGL---

PvaHc --------ATGLPNRFLLPKGNDR-GLEFDLVVAVTDGDADSAVPNL---

FchHc1 --------ATGIPNRFLLPKGNEQ-GLEFDLVVAVTDGEADAAVEGL---

MjaHcL --------ATGIPNRFLLPKGNEK-GLEFDLVVAVTDGAADAAVDGL---

MjaHcY --------ATGLPNRFLLPKGNEQ-GLEFDLVVAVTDGDADAAVADL---

CjaHc1 --------ATGMPNRFLIPKGNKN-GMEFDLIVCVTDGKADAAIDNL---

PmaHc2 --------HCGFPHRLLVPKGRPE-GMHYKLMVVITDYHKDVVVPDM-DV

StuHc2 --------HCGFSQGLLIPKGSEA-GTHFKVFIMLTDWDKDHANADA--H

PgrHc2 --------HCGFPHRLLVPKGRPE-GMHYKLMVVITDYHKDVVVPDM-DV

CseHc2 --------HCGFPRRLLLPKGKPE-GMVYQLYVVVTDYDKDIVEPGI-QP

PamHc2 --------HCGFPHRLLLPKGKPE-GMVYRLFVVVTDYDKDVVAPGM-SP

BduHc2 --------HCGLPHRLLLPKGKPE-GMDFKLYVVISDFDKDVVSSEV-NV

HmeHc2 --------HCGFPHRLLLPKGKPE-GMTYRIYVIITDYNKDIITSDW-KP

TdoHc2 --------HCGYPHRLLLPKGTSG-GMVFNLYVTITNYEQDKVHPGV-TV

StuHc3 --------HCGIPDRMLLPKGKVG-GMEFMLLVVVTDGGADKGVTIH---

StuHc1 --------HCGIPDRLLLPKGSKE-GTPFTLFVMVTDFDDDNANTDV--E

PmaHc1 --------HCGYPDRLLLPKGRNT-GMPVQIYVIVTDFEKEKVNDLP---

SamHc1 --------HCGYPDRLLLPKGRRD-GMPFTLFVVLTDYEKDKVNDLP---

BduHc1 --------HCGYPDRLLLPKGKKE-GMPFTLYVIVTDFEKEKVNDLP---

PamHc1 --------HCGYPDRLLLPKGKKD-GMIFTMYVIVTDYETEKVNDLP---

CseHc1 --------HCGYPERLLLPKGKRD-GMPFSLFVILTDFDKEKVNDLP---

HmeHc1 --------HCGYPDRLLLPKGKKD-GMPFTFYVIVTDFDKEKVNDVP---

CmoHc1 --------HCGYPDRLLLPKGKKG-GMPFTLYVILTDFNKEKVNDLP---

CacHc1 --------HCGYPGRLLLPKGKIE-GMPFPLYAIVTDFEQEKVNDLP---

TdoHc1 --------HCGFPDRLLLPKGKKE-GMPFTLFVMVTDWEKEKVNDVP---

PgrHc1 --------HCGYPDRLLLPKGRKQ-GMPYQIYVIVTDFEKEKVNDLP---

ScuHc1 --------HCGVPDRLLLPKGKTN-GMKYTMFVMLSDFEEDKVNDLP---

MgeHc1 --------HCGLPDRLLIPKGNEE-GYPVKFFVIVTDWEEDKVNQEV---

PlaPPO K---CCDPTSYCGAK--NQLYPDKKSMGFPFDRPASAATRSLGDFLRDKS

AfrPPO K--PCSRGTSYCGVL--DDKYPDKRPMGFPFDRPIEDATTTYEDFTEGLT

DmaPPO K--LCRNAASYCGIL--DEKYPDKRPMGFPFDRPPDKMITTLAKFIEKSP

DmePPOA1 D--ACSTAYSFCGLK--DKLYPDRRTMGYPFDRRLPN--ANLTELVGAFG

DmePPO2 -VGRCSDAASYCGVR--DRLYPDRQSMGFPFDRLPRSGVDRLVNFLT--P

PseHc R--TCDDSLTMCGLL--DKKFPDKRSMGFPLDRPIPS--TTSNWFNR--P

EpiHc1 -KPTCQDAVSYCGII--NDLYPDKKPMGFPFDRLIPS---DLATFVS-RS

EspHc1 C--LCKDAVSYCGSK--DEKYPDKKPMGFPFDRKIGF--NRLSNFLT--P

PimHc2 A--ICSDAVSYCGAK--DEKFPDKHAMGFPFDRPIHS--HTLADFST--H

PimHc3A KA-LCTDAVSYCGAR--DEKYPDKKAMGFPFDRKIPA--ANHKEFLT--S

PimHc3B S--ICSDAVSYCGAK--DDKYPDKKSMGFPFDRTMPY--ETSDEFLT--P

PimHc3C G--ICVDAVSYCGAR--DDLYPDRRAMGFPFDRRIEA--DTVEEWLL--P

PimHc4 L--ICADALSYCGAK--DHLYPDKKPMGFPFDRPFEA--HSFQEIIT--P

PimHc5A A--ICSDAVSYCGAK--DSKYPDKKAMGFPFDRVIHA--RTPAQFKT--P

PimHc5B T--VCREAVSYCGLK--DELYPDKRPMGFPFDRYIAE--EHLHDWLL--P

PimHc6 A--VCRDAISYCGAK--DDKYPDKKAMGFPFDRKIEA--RTGAEFLT--P

AauHc6 A--VCSDAVSYCGAR--DDRYPDKKAMGFPFDRKIEA--RTAAEFLT--P

LpoHcII -SVACVDAVSYCGAR--DHKYPDKKPMGFPFDRPIHT--EHISDFLT--N

LpoHcIIIa H--MCDDAVSYCGAK--DSKYPDKKPMGFPFDRRIDA--HDIEEFLT--P

LpoHcIIIb L--TCADAVSYCGAR--DHLYPDKKPMGFPFDRPIKA--HTVEEFLT--P

LpoHcIV L--ICADAVSYCGAR--DHKYPDKKPMGFPFDRPIDA--RTPSQFAT--P

LpoHcVI G--MCTDAVSYCGAK--DQLYPDRRPMGFPFDRHIEL--EHLKDWVL--P

CroHcVI G--MCTDAVSYCGAK--DQLYPDRRPMGFPFDRDIEE--EHLSDWLL--P

CroHcV S--ICVDAVSYCGAK--DQLYPDRRAMGFPFDRAIES--DHLEEWIL--P

CroHcIV L--ICADAVSYCGAR--DHKYPDKRPMGFPFDRPIDA--RTPSQFAT--P

CroHcIIIb L--TCADAVSYCGAR--DHLYPDKKPMGYPFDRPIKA--HTVEEFLT--P

CroHcIIIa P--MCDDAVSYCGAK--DSKYPDKKHMGFPFDRRIDA--PDIEEFLT--P

CroHcII -SVACVDAVSYCGAR--DHKYPDMKPMGFLFDRPILT--EHISDFLT--N

CroHcI ---FCSDAVSYCGAK--DHKYPDKKPMGFPFARKIKN--EPLEDFLT--T

AgoHcA P--LCADALSYCGVL--DQKYPDKRAMGYPFDRMIIA--DTHEEFLT--G

AgoHcB G--LCTDAVSYCGAK--DQLYPDRYPMGFPFDRDIKA--DSILEWVN--P

AgoHcC T--PCVDAVSYCGAK--DSLYPDRRAMGFPFDRPIPE--EHASNLHQ--P

AgoHcD T--ICSDAVSYCGAK--DQKYPDKKAMGYPFDRPIQV--RTPSQFKT--P

AgoHcE A--ECVDAASYCGAK--DQKYPDKKPMGFPFDRVIDA--LTLEEFLT--P

AgoHcF T--LCADAVSYCGAK--DSKYPDKRAMGFPFDRVIKA--RTVPDFGT--K

AgoHcG T--VCADAVSYCGAK--DQKYPDTKPMGFPFDRPTKI--HTAEEILT--P

AgoHcX P--KCKESVSYCGLK--DQKYPDKKAMGFPFDRRITD---TATDFAT--P

EcaHcA P--LCADALSYCGVL--DQKYPDKRAMGYPFDRKITA--DTHEEFLT--G

NinHcA ---LCNDALSYCGVM--DEKYPDKRAMGYPFDRTITA--QSHEEFIT--P

EcaHcB G--LCTDAVSYCGAK--DQLYPDRYPMGFPFDRDIKA--DSIPEWLH--P

NinHcB N--ECVDAVSYCGAK--DQLYPDKRAMGFPWDREIVA--NDFNEWRQ--P

EcaHcC T--PCVDAVSYCGAK--DSLYPDRRAMGFPFDRPIPE--GHASNLHQ--P

EcaHcD T--ICSDAVSYCGAK--DQKYPDKKAMGYPFDRPIQV--RTPSQFKT--P

NinHcD V--VCSDAVSYCGAK--DQKYPDRRAMGYPFDRPIKA--RTPSQFKT--Q

EcaHcE A--ECVDAVSYCGAK--DQKYPDKKPMGFPFDRVIEG--LTLEEFLT--P

NinHcE G--VCVDAISYCGAK--DQKYPDKKPMGFPFDRVITA--DTVAEFLT--P

EcaHcF T--LCADAVSYCGAK--DSKYPDKRAMGFPFDRVIKA--RTVADFRT--T

NinHcF S--ICADAVSYCGAK--DQKYPDKKSMGFPFDRVIKA--RTLAEFSS--A

EcaHcG I--VCADAVSYCGAK--DQKYPDTKPMGFPFDRPTKI--HTAEEILT--P

NinHcG T--ICNDAVSYCGAK--DQKYPDKKPMGFPFDRPLLS--SVAAKLPT--E

TtrHcA ---FCSDAVSYCGAK--DHKYPDKKPMGFPFDRKIEN--EHLEDFLT--T

EbaHcA AEHACSDAVSYCGVM--DEKYPDKKAMGFPFDRTIET---TREGFFT--P

MgiHcA P--LCTDAVSYCGVI--DDKYPDKKAMGFPFDRYISS---DREKFLS--P

EbaHcB G--RCTEAVSYCGAK--DQLYPDKKAMGFPFDREIEI--DHLEEWLT--P

MgiHcB G--VCFDAVSYCGAK--DQLYPDKRAMGFPFDREVDE--DHLEKWLL--P

EbaHcC A--ICVDAVSYCGAK--DQLYPDRKPMGFPFDRIIEA--KTMKEWLY--P

MgiHcC D--ICMDAVSYCGAK--DQFYPDRRPMGFPFDRVIQE--EEMGQWQL--P

EbaHcD S--LCVDAVSYCGAK--DEKYPDKRAMGFPFDRTITA--RNPTMFKT--H

MgiHcD H--LCSDALSYCGVK--DEKYPDKRAMGFPFDRTITQ--RFPTQFST--H

EbaHcE G--SCADAFSYCGVK--DHKYPDSKAMGFPFDRHIVN--HDIHGFLP--S

MgiHcE G--VCVDSLSYCGIK--DHKYPDKKPMGYPFDRPIDV---DIEEFLT--P

EbaHcF F--ICADAVSYCGAK--DDRYPDKKAMGFPFDREITA--HGPVDFAT--P

MgiHcF H--VCHDAFSYCGVK--DQKYPDKKPMGFPFDRVIKA--RSIAQFKT--H

EbaHcG R--ICMDAFSYCGTK--TGMYPDLKPMGFPFDRPIDH--DHADELFT--D

MgiHcG M--TCADAVSYCGAL--DHKYPDKRPMGFPFDRPVVD--KTEEELTAHVS

CsaHc1 T--ICGDAVSYCGAK--DQKYPDKKPMGYPFDRPTTA--RSAQELLT--D

CsaHc2 T--ICSDAVSYCGAK--DQKYPDKKPMGFPFDRPIVA--RSAEELLT--E

CsaHc3 T--TCADAVSYCGAK--DQKYPDLKPMGFPFDRPVSA--RTAEELLT--E

CsaHc4 T--ICSDAVSYCGAK--DQKYPDKKPMGFPFDRHLTA--RTAEELLT--D

CsaHc5a T--ICSDAVSYCGAK--DQKYPDRKPMGFPFDRPTTA--RTAEELLT--D

CsaHc5b T--ICSDAVSYCGAK--DQKYPDRKPMGFPFDRPTTA--RTAEELLT--D

CsaHc6a T--ICSDAVSYCGAK--DQKYPDLKPMGYPFDRHLTA--RNEAELLT--D

CsaHc6b T--ICSDAVSYCGAK--DQKYPDLKPMGYPFDRHLTA--RNEAELLT--D

CsaHc6c T--ICSDAVSYCGAK--DQKYPDLKPMGYPFDRHLTA--RNEAELLT--D

HauHcB C--NCGSALSYCGIV--SGKYPNSKAMGYPFDRHIDA--HNVEDWVT--P

HauHcA C--NCGSALSYCGIV--SGKYPNSKAMGYPFDRHISA--HSVEDWVT--P

ScoHcA C--QCRDSMSYCGSV--EHKLPDNKPLGYPFDRRIDG--TGFEEFKT--Q

ScoHcD C--LCGDAVAYCGAH--NQKYPDKKPMGFPFDRRIDE--RTFEHFHT--P

ScoHcX C--FCADSWSHCGSL--FIQYPENVEMGFPFQPIIEC--TKEEFFAL--P

ScoHcC C--ACHDALTYCGGI--DYHFPDKRAMGFPFDRPIKQ--RNFNAFKT--K

ScoHcB C--HCGDSLSYCGAL--YDKYPDRRPMGYPFDRHADA--QTFDEFKT--K

SpiHc1 C--KCH-AHSYCGNI--LGEYLDKRPLGYPFDRKIKA--TGWAEFKT--Q

AgiHc1 C--KCH-AHSYCGNI--LGEYLDKRPLGYPFDRKIKA--AGWEEFKT--Q

PanHc1 C--HCKNSLCYCGTI--FQEYPDKRPMGFPFDRPLPK--SGWEAFRT--P

PanHc2 C--FCKDSLTYCGGI--DSKYRDTKPFGFPYDRKIKA--ESWQDWET--D

PciHc1 ----EDGNHGYCGMH--GQKYPDKKPMGFPYDRHISD-----RRMYDGLK

PciHc2 ----EDGNHGYCGMH--GQKYPDKKPMGFPYDRHIPD-----RRLYDGLK

NkeHc -DNEHGSNHGYCGIH--NELYPDKQPMGFPLDRKIED-----RNMFVGMP

GpuHc1 AVDAHG--HAQCGVH--GEKYPDHQPMGFPLDRRIPD-----DRIFLSAD

GroHc1 AVDAHG--HAQCGVN--GEKYPDHQPMGFPLDRRIPD-----DRIFLSAD

CcsHc -EIQGAHSHAQCGVQ--GEKYPDSKPMGFPLDRRIED-----ERILLSSS

EpuHc1 -KDEHGGSHAQCGIH--GEVYPDKRPLGFPLDRQIPD-----ERILEHFP

EpuHc2 -KDEHGGSHAQCGMH--GEVYPDKRPLGYPLDRHIPD-----ERTLEHFP

OscHc1 -KSEHGSAHGYCGVQ--GEIYPDKRPMGYPLDRRIPD-----DRLFH-VP

OscHc3 -SSEHGSAHGYCGVR--GEIYPDKRPMGYPLDRRIPD-----DRLYH-VP

OscHc2 -SNEHDSNHGYCGTH--GQVYPDKRPMGYPLDRKIPD-----ERVFH-MD

OscHc4 -TNEHGSTHGYCGIH--GQVYPDKRPMGFPLDRKVTD-----EREFH-ID

PleHc -VNAHGSTHGYCGIH--GEKYPDKRPMGFPFDRPIPD-----LRVFK-VQ

PleHc2 --EELGATHAQCGIH--GEKYPDKKPMGYPVDRSVPD-----NRVFLESP

HamHcA -TDEHGGTHAQCGAH--GEKYPDKKPMGYPLERSIPD-----ERVFHDVA

PinHcB -GHDYGGTHAQCGVH--GEAFPDNRPLGYPLERRIPD-----ERVIDGVS

PinHcA -GHDYGGTHAQCGVH--GEAYPDNRPLGYPLERRIPD-----ERVIDGVS

PvuHc -DHDHHGTHAQCGIH--GELYPDHRPLGYPLERRIPD-----DRVFDGVS

PelHc1 -DHDHHGTHAQCGIH--GEQYPDHRPLGYPLERRIPD-----ERVFDGVS

PelHc2 -DHDHHGTHAQCGIH--GEQYPDHRPLGYPLERRIPD-----ERVFDGVS

PelHc3 -DHDHHGTHAQCGIH--GEQYPDHRPLGYPLERRIPD-----ERVFDGVS

PelHc4 -DHDHHGTHAQCGIH--GEQYPDHRPLGYPLERRIPD-----ERVFDGVS

CmaHc6 ---HENTSFNHYGAH--NGKYPDKRPHGYPMDRRVDD-----KRIFSGVT

CmaHc1 -HTDIHGNHGYCGIQ--GAKYPDKRPMGFPFERRVPD-----IRVIKNLP

CmaHc2 -TNDYGSNHGYCGIQ--GAKYPDKRPMGFPYERRIPN-----KFLVLGLP

CmaHc3 ---HENTSFNHYGCA--DGTYPDKRPHGYPLDRHVDD-----ERIINDLH

CmaHc4 ---LENTSFNHYGAH--SGKYPDKQPHGYPLDRRVDD-----KRIITGVT

CmaHc5 ---YENTSFNHYGAH--NGKYPDKQPHGYPLDRRVDD-----ERIITGVT

CsaHc ---HESTTYNHYGCH--DGTYPDNQPHGYPLDRRVDD-----ERIITGVS

PinHcC ---HENTKFIHYG-Y--DRQYPDKRPHGYPLDRRVDD-----ERIFEALP

PvaHc1 ---HENTEFNHYGSH---GVYPDKRPHGYPLDRKVPD-----ERVFEDLP

PvaHc ---HENTEYNHYGSH---GVYPDKRPHGYPLDRKVPD-----ERVFEDLP

FchHc1 ---HDNTDFIHYGSH---GKYPDNRPHGYPLDRKVPD-----DRVFEVLP

MjaHcL ---HENTEFNHYGAY---GKYPDNRPHGYPLDRSVPD-----ERVFEDLP

MjaHcY ---HQNTDYNHYGAH---GVYPDKKPHGYPLDRKVPD-----ERVFEELS

CjaHc1 ---HEVHDFVHFGHH---GTYPDKRPHGYPLDRRYDD-----ARVFHEVP

PmaHc2 EHMDKLQSVGYCGVM--EGKIPDGKPMGYPFDRRISC-----EESFI-TK

StuHc2 PEDDYGGSIGYCGAL--WAKYPDKKPMGFPFDRHIQD-----EEDFF-TE

PgrHc2 EHMDKLQSVGYCGVM--EGKIPDGKPMGFPFDRPVPC-----EETFI-TK

CseHc2 EDVENLSSLGYCGVM--DGKIPDGRPMGFPFDRRIVS-----PEQFL-TP

PamHc2 DDVNTLSSLGYCGVM--DGKMPDGKPMGFPFDRRVPS-----EETFY-TP

BduHc2 DDLHHISSLGYCGVL--DGKIPDGKPMGFPFDRRIPS-----EELFL-TP

HmeHc2 EDIENLSSLGYCGVL--EGKIPDARPMGFPFDRRITS-----EEEFF-TQ

TdoHc2 DEVDNLTSLGYCGVL--DGVIPDGRPMGYPYDRPIPD-----KEVFN-IP

StuHc3 DDHIYGGSTSLCGIR--GEKYPDKRALGFPFDRYIHS-----VEDFV-TP

StuHc1 STHDYGGSISYCGTLTEGQKYPDKKPMGFPFDRHIED-----VHDFK-TK

PmaHc1 YDYDYGGSLSYCGVVG-GHKYPDTKAMGFPFDRRIYS-----REDFF-TD

SamHc1 FDYDYGGSVSYCGTL--GHRYPDAKPMGFPFDRRIDQ------DSFF-TK

BduHc1 WDYDYGGSISYCGVLS-GHKYPDSKPMGFPFDRHIDG------DHFF-TE

PamHc1 YDYEYGGAISYCGTLA-GHKYPVNKPMGFPFDRQIDG------DNFC-TP

CseHc1 WDYDYGGSISYCGTVS-GHKYPDSKPMGFPFDRQINS------DNFF-RS

HmeHc1 LDYNYGGSISYCGTLA-GHKYPDNKPMGFPFDRRIDE------DHFF-TP

CmoHc1 YDYDYGGSLSYCGTI--NHKYPDSRPMGFPFDRRLST------EEFS-MP

CacHc1 FDYDYGGSISYCGTL--GHKYPDSKPMGYPFDRPIGR-------EFY-YP

TdoHc1 YDYDYGGSISYCGTL--NHKYPDTQPMGYPFDRRIEN-----VEEFL-TP

PgrHc1 YDYDYGGSLSYCGVVG-GHKYPDTKAMGFPFDRRIYS-----REDFF-TD

ScuHc1 HDYEYGGFVSYCGTI--NHKYPDKKPMGWPLDRKICK------DSFHDVT

MgeHc1 ENHRYGNVYSYCGTYG-DRLYPDKKAFLYPFDRVIKD-----VNVFK-TP

PlaPPO NMLTTDVVLVHT

AfrPPO NIFKREVVVQFK

DmaPPO NIKTTEIRIQFE

DmePPOA1 NMAKTDLRIVFN

DmePPO2 NMSIVDVNIRHE

PseHc NMMRQDVTIYHF

EpiHc1 NMKIFDISIIHR

EspHc1 NMKSTEVRILFK

PimHc2 NMATTAVEIVFK

PimHc3A NMYLKEVTIRYI

PimHc3B NMKCKEITIKFH

PimHc3C NMSDTIIKITHV

PimHc4 NINVSNVVIKFD

PimHc5A NMSFTDVRIQHK

PimHc5B NMTSTDITIKHQ

PimHc6 NMAVQDVKIKFS

AauHc6 NMGLTDIKIKFH

LpoHcII NMFIKDIKIKFH

LpoHcIIIa NMALTDVKIKFE

LpoHcIIIb NMSLTDIKIQYK

LpoHcIV NMSFTDIRIQFS

LpoHcVI NMKDTLITVTHH

CroHcVI NMKDVLITVTHH

CroHcV NMRNTVITVTYH

CroHcIV NMSFTDIRIQFV

CroHcIIIb NMSLTDVKIQYK

CroHcIIIa NMTLTDIKIKFE

CroHcII NMFIKDIHIKFH

CroHcI NMGVSAIKIQFM

AgoHcA NMNISHVTVRFQ

AgoHcB NMHFSEVTITHH

AgoHcC NIRFTQIKIQHH

AgoHcD NMAFQEIIIQYE

AgoHcE SMSCTDVRIKYT

AgoHcF NMSFTDVKIQFK

AgoHcG NMSLTDVVIQFV

AgoHcX NMKFTDIKVQFT

EcaHcA NMNISHVTVRFQ

NinHcA NMKISNITVRFQ

EcaHcB NMHFSEVTITHH

NinHcB NMISIPIDIVHS

EcaHcC NVSFSQIKIQHH

EcaHcD NMAFQEIIIQYE

NinHcD NMSFTEVRIQYG

EcaHcE SMSCTDVRIKYT

NinHcE NMSCTDVKIKHV

EcaHcF NMSFTDVKIQFK

NinHcF NMKFQEVKIQFK

EcaHcG NMSLTDVVIQYV

NinHcG NTCVTDIKIKFL

TtrHcA NMGVSDIKIQFM

EbaHcA NMSLTEVTVRYD

MgiHcA NMRLTKIIIKHD

EbaHcB NMTQVEVTIKHE

MgiHcB NMSNTIVTILHH

EbaHcC NMEDVIVTIKHH

MgiHcC NMSNTHIKIQHQ

EbaHcD NMHFSDVVIQFN

MgiHcD NMSFTDIRIQFV

EbaHcE NASVTEVKIKFT

MgiHcE NMTVADVKIKFR

EbaHcF NMSFTDIKIQFK

MgiHcF NMSFTDVTIQFK

EbaHcG NHHRSTVTIQYV

MgiHcG NLHRTDVKIKFL

CsaHc1 NMSLTTVKIQYL

CsaHc2 NMSLTDVKIKFL

CsaHc3 NMSLTDVLIKYV

CsaHc4 NMAITDVKIKFL

CsaHc5a NMTLTDVKIKFA

CsaHc5b NMTLTDVKIKFA

CsaHc6a NMVMTDVVIKFL

CsaHc6b NMVMTDVVIKFL

CsaHc6c NMVMTDVVIKFL

HauHcB NIQHVDITIKFA

HauHcA NINYADITIKFT

ScoHcA NMYYGDVVIQFT

ScoHcD NMIATDVIIKFT

ScoHcX NIAKQEVIIKFT

ScoHcC NMGKVTVDVKFT

ScoHcB NMNSVTVTIKHT

SpiHc1 NMCDADIVIKFS

AgiHc1 NMCDTDVVIKFS

PanHc1 NMFRKDLTVKFT

PanHc2 NIAHTDITVKFV

PciHc1 NLHTQTVKIFHH

PciHc2 NLHTQTVKIFFR

NkeHc NINYNIVNVFHK

GpuHc1 NIEKLIVTVHHK

GroHc1 NIEKLLVTVTHK

CcsHc NLKYTVVKITHK

EpuHc1 NLHKSIVVVHHE

EpuHc2 NLHKSVVTIRHD

OscHc1 NINVSTVKVYHK

OscHc3 NIHLSTVKVYHK

OscHc2 NIKITTVTVSHK

OscHc4 NINVSTVKVFHK

PleHc NQHGQVVKIFHH

PleHc2 NIKRVYVKVFHD

HamHcA NINVTHVKVFFK

PinHcB NIKHVVVKIVHH

PinHcA NIKHVVVKIVHH

PvuHc NIKHALVKIVHD

PelHc1 NIKHALVKIVHD

PelHc2 NIKHALVKIVHD

PelHc3 NIKHALVKIVHD

PelHc4 NIKHALVKIVHD

CmaHc6 NIKCMNVKVYHV

CmaHc1 NFFGKIVDVYHK

CmaHc2 NHHSQEVNVYHK

CmaHc3 NFKHIQVKVFHH

CmaHc4 NFKGMDVKVYHV

CmaHc5 NFKGVDVKVYHV

CsaHc NFKAVDVKVYHV

PinHcC NFKQRTVKLYSH

PvaHc1 NFGHIHLKVFNH

PvaHc NFKHIQVKVFNH

FchHc1 NFKHIQVKVFNH

MjaHcL NFGHIQVKVFNH

MjaHcY NFKRIQVKVFNH

CjaHc1 NYKHTIVKIFHR

PmaHc2 NMKFVDITVKTR

StuHc2 NMKLIDVVIKNI

PgrHc2 NMKFVDITVKTR

CseHc2 NMEVVDITIKNV

PamHc2 NIKTIDLTIKNV

BduHc2 NMKVVNLVIKNV

HmeHc2 NMKVVDVTIPNV

TdoHc2 NSKVIEVTIKNA

StuHc3 NMFCKDVVITHV

StuHc1 NMIVKDVVVTHK

PmaHc1 NMYTKDVTITFK

SamHc1 NIYQRDVTITFK

BduHc1 NMYQKEVKITFQ

PamHc1 NMFQKDVIITFK

CseHc1 NIYQKDVVITFK

HmeHc1 NMGQKEVTISFK

CmoHc1 NFCSKDVTITFK

CacHc1 NMFEKDVVITHK

TdoHc1 NMYVKDVVITHN

PgrHc1 NMYTKDVTITFN

ScuHc1 NMYFRDVEIKFE

MgeHc1 NMFGKYVSIYHK

;

End;

begin mrbayes;

prset aamodelpr=fixed(WAG);

lset rates=gamma;

mcmc ngen=5000000 printfreq=100 samplefreq=100 nchains=4 savebrlens=yes;;

End;
