# Supplementary material for: The diversity and evolution of chelicerate hemocyanins
Source: BMC Evol Biol. 2012 Feb 14;12:19. doi: 10.1186/1471-2148-12-19 (PMC3306762; doi:10.1186/1471-2148-12-19)
Supplement: Additional file 3 — Molecular properties of chelicerate hemocyanin cDNA and the deduced amino acid. The asterisks (*) denote incomplete N-terminal sequences of P. imperator hemocyanins subunits, with 8 and 9 amino acids missing. [file 1471-2148-12-19-S3.DOC]

**Additional file 3.** Molecular properties of chelicerate hemocyanin cDNA and the deduced amino acid. The asterisks (*) denote incomplete N-terminal sequences of *P. imperator* hemocyanins subunits, with 8 and 9 amino acids missing.

| Species | subunit | accession no. | nucleotide [bp] | amino acids | molecular mass [kDa] |
| --- | --- | --- | --- | --- | --- |
|  |  |  | cDNA |  |  |
| *Endeis spinosa* | EspHc1 | FR865911 | 2084 | 631 | 72.2 |
| *Carcinoscorpius rotundicauda* | CroHcI | DQ090484 | 2175 | 624 | 71.7 |
| *Carcinoscorpius rotundicauda* | CroHcII | DQ090485 | 2240 | 629 | 72.6 |
| *Carcinoscorpius rotundicauda* | CroHcIIIa | DQ090486 | 2054 | 631 | 73.0 |
| *Carcinoscorpius rotundicauda* | CroHcIIIb | DQ090487 | 2155 | 628 | 72.8 |
| *Carcinoscorpius rotundicauda* | CroHcIV | DQ090488 | 2188 | 624 | 72.6 |
| *Carcinoscorpius rotundicauda* | CroHcV | DQ090489 | 2581 | 638 | 74.0 |
| *Carcinoscorpius rotundicauda* | CroHcVI | DQ090490 | 2268 | 631 | 72.9 |
| *Limulus polyphemus* | LpoHcII | AM260213 | 2252 | 629 | 72.8 |
| *Limulus polyphemus* | LpoHcIIIa | AM260214 | 2054 | 627 | 72.3 |
| *Limulus polyphemus* | LpoHcIIIb | FR865912 | 2147 | 628 | 72.8 |
| *Limulus polyphemus* | LpoHcIV | AM260215 | 2183 | 624 | 72.8 |
| *Limulus polyphemus* | LpoHcVI | AM260216 | 2223 | 638 | 73.4 |
| *Tachypleus tridentatus* | TtrHcA | None | amino acids | 622 | 71.4 |
| *Androctonus australis* | AauHc6 | P80476 | amino acids | 626 | 71.8 |
| *Pandinus imperator* | PimHc2* | FN424080 | >1965 | >616 | >70.2 |
| *Pandinus imperator* | PimHc3A | FN424079 | 2494 | 627 | 72.4 |
| *Pandinus imperator* | PimHc3B | FN424082 | 2063 | 632 | 72.7 |
| *Pandinus imperator* | PimHc3C | FN424081 | 2246 | 637 | 73.6 |
| *Pandinus imperator* | PimHc4* | FN424085 | >1897 | >616 | >70.4 |
| *Pandinus imperator* | PimHc5A | FN424084. | 2079 | 627 | 72.0 |
| *Pandinus imperator* | PimHc5B | FN424086 | 2189 | 633 | 73.7 |
| *Pandinus imperator* | PimHc6 | FN424083 | 1995 | 625 | 71.7 |
| *Euphrynichus bacillifer* | EbaHc-a | FR865913 | 2066 | 631 | 72.5 |
| *Euphrynichus bacillifer* | EbaHc-b | FR865914 | 2044 | 627 | 72.3 |
| *Euphrynichus bacillifer* | EbaHc-c | FR865915 | 2064 | 630 | 72.4 |
| *Euphrynichus bacillifer* | EbaHc-d | FR865916 | 2033 | 627 | 72.1 |
| *Euphrynichus bacillifer* | EbaHc-e | FR865917 | 1994 | 626 | 71.2 |
| *Euphrynichus bacillifer* | EbaHc-f | FR865918 | 2101 | 626 | 72.1 |
| *Euphrynichus bacillifer* | EbaHc-g | FR865919 | 2056 | 623 | 71.7 |
| *Mastigoproctus giganteus* | MgiHc-a | FR865913 | 2103 | 631 | 72.7 |
| *Mastigoproctus giganteus* | MgiHc-b | FR865914 | 2148 | 627 | 72.3 |
| *Mastigoproctus giganteus* | MgiHc-c | FR865915 | 2226 | 639 | 73.7 |
| *Mastigoproctus giganteus* | MgiHc-d | FR865916 | 2099 | 629 | 72.7 |
| *Mastigoproctus giganteus* | MgiHc-e | FR865917 | 2014 | 621 | 71.7 |
| *Mastigoproctus giganteus* | MgiHc-f | FR865918 | 2085 | 622 | 72.1 |
| *Mastigoproctus giganteus* | MgiHc-g | FR865919 | 2050 | 626 | 72.0 |
| *Acanthoscurria gomesiana* | AgoHc-x | EST | >2110 | >642 | 73.8 |
| *Acanthoscurria gomesiana* | AgoHc-a | EST | 2141 | 631 | 72.3 |
| *Acanthoscurria gomesiana* | AgoHc-b | EST | 2179 | 627 | 72.1 |
| *Acanthoscurria gomesiana* | AgoHc-c | EST | 2184 | 630 | 72.7 |
| *Acanthoscurria gomesiana* | AgoHc-d | EST | 2089 | 627 | 71.9 |
| *Acanthoscurria gomesiana* | AgoHc-e | EST | 2258 | 624 | 71.3 |
| *Acanthoscurria gomesiana* | AgoHc-f | EST | 2222 | 629 | 72.0 |
| *Acanthoscurria gomesiana* | AgoHc-g | EST | 2097 | 629 | 71.7 |
| *Eurypelma californicum* | EcaHc-a | X16893 | 2110 | 631 | 72.2 |
| *Eurypelma californicum* | EcaHc-b | AJ290429 | 2144 | 627 | 72.0 |
| *Eurypelma californicum* | EcaHc-c | AJ277489 | 2174 | 629 | 72.4 |
| *Eurypelma californicum* | EcaHc-d | AJ290430 | 2042 | 627 | 72.0 |
| *Eurypelma californicum* | EcaHc-e | X16894 | 2192 | 624 | 71.5 |
| *Eurypelma californicum* | EcaHc-f | AJ277491 | 2060 | 629 | 72.0 |
| *Eurypelma californicum* | EcaHc-g | AJ277492 | 2088 | 629 | 71.7 |
| *Nephila inaurata* | NinHc-a | AJ547807 | 2067 | 630 | 72.1 |
| *Nephila inaurata* | NinHc-b | AJ547808 | 2060 | 628 | 72.6 |
| *Nephila inaurata* | NinHc-d | AJ547809 | 2327 | 627 | 71.9 |
| *Nephila inaurata* | NinHc-e | AJ547810 | 2106 | 625 | 71.0 |
| *Nephila inaurata* | NinHc-f | AJ547811 | 2152 | 626 | 71.7 |
| *Nephila inaurata* | NinHc-g | AJ547812 | 2059 | 626 | 71.2 |
| *Cupiennius salei* | CsaHc1 | AJ307903 | 2084 | 634 | 72.7 |
| *Cupiennius salei* | CsaHc2 | AJ307904 | 2048 | 626 | 71.0 |
| *Cupiennius salei* | CsaHc3 | AJ307905 | 2039 | 626 | 71.3 |
| *Cupiennius salei* | CsaHc4 | AJ307906 | 2030 | 626 | 71.1 |
| *Cupiennius salei* | CsaHc5 | AJ307907 | 2101 | 626 | 71.4 |
| *Cupiennius salei* | CsaHc5' | AJ307908 | 2101 | 626 | 71.5 |
| *Cupiennius salei* | CsaHc6 | AJ307909 | 2266 | 626 | 71.6 |
| *Cupiennius salei* | CsaHc6' | AJ307910 | 2264 | 626 | 71.5 |
| *Cupiennius salei* | CsaHc6'' | AJ307911 | 2265 | 626 | 71.6 |
