# Supplementary material for: The diversity and evolution of chelicerate hemocyanins
Source: BMC Evol Biol. 2012 Feb 14;12:19. doi: 10.1186/1471-2148-12-19 (PMC3306762; doi:10.1186/1471-2148-12-19)

**Additional file 4.** Phylogenetic tree of the chelicerate hemocyanin subunits excluding AgoHcX. The numbers at the nodes represent Bayesian posterior probabilities estimated with the WAG model of amino acid substitution. The species abbreviations are: Aau, *Androctonus australis*; Ago, *Acanthoscurria gomesiana*; Cro, *Carcinoscorpius rotundicauda*; Csa, *Cupiennius salei*; Eba, *Euphrynichus bacillifer*; Eca, *Eurypelma californicum*; Esp, *Endeis spinosa*; Lpo, *Limulus polyphemus*; Mgi, *Mastigoproctus giganteus*; Nin, *Nephila inaurata*; Pin, *Pandinus imperator*; Ttr, *Tachypleus tridentatus*. See Supplementary Table 1 for abbreviations of the proteins.


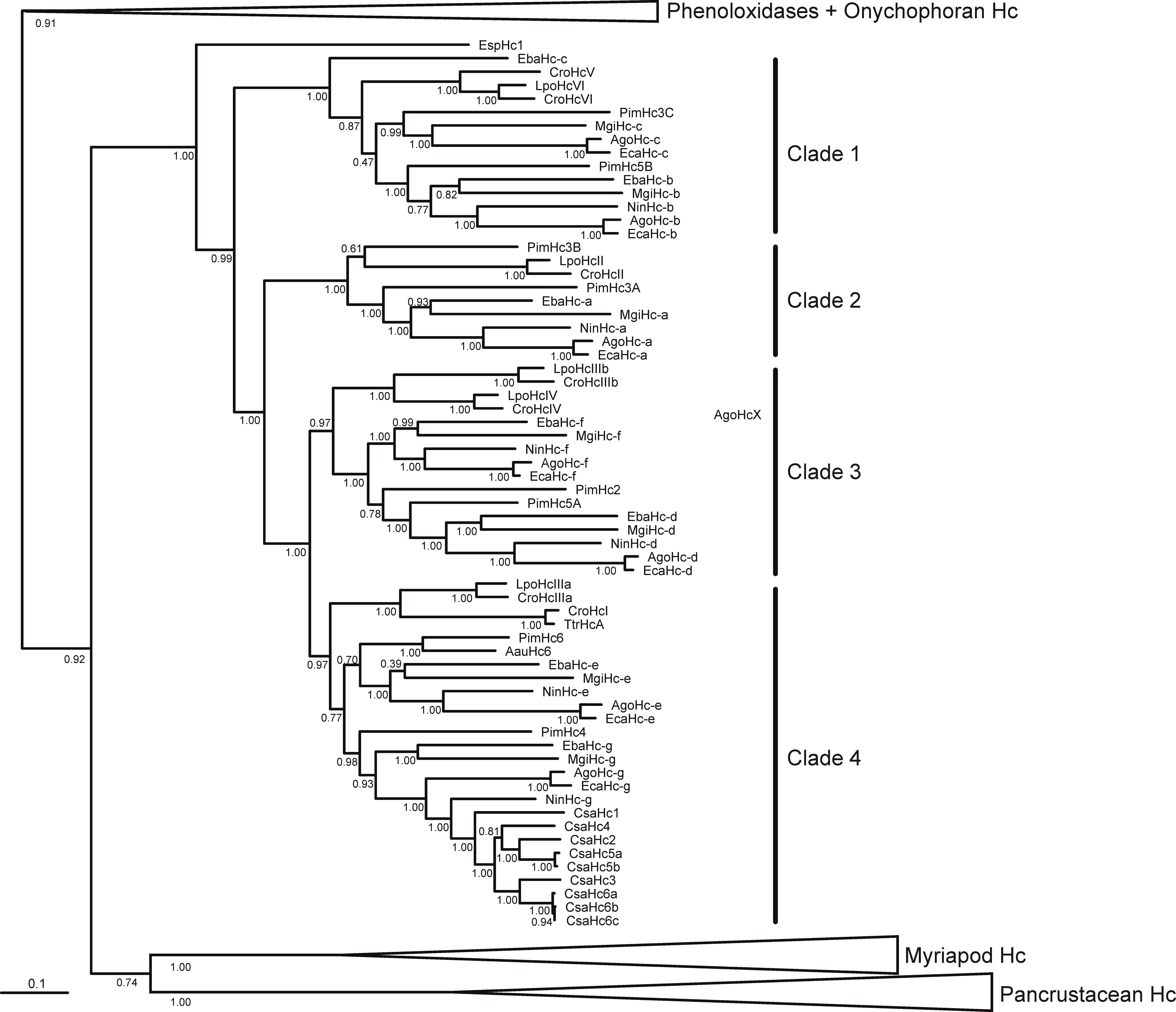

Supplement: Additional file 4 — Phylogenetic tree of the chelicerate hemocyanin subunits excluding AgoHcX. The numbers at the nodes represent Bayesian posterior probabilities estimated with the WAG model of amino acid substitution. The species abbreviations are: Aau, Androctonus australis; Ago, Acanthoscurria gomesiana; Cro, Carcinoscorpius rotundicauda; Csa, Cupiennius salei; Eba, Euphrynichus bacillifer; Eca, Eurypelma californicum; Esp, Endeis spinosa; Lpo, Limulus polyphemus; Mgi, Mastigoproctus giganteus; Nin, Nephila inaurata; Pin, Pandinus imperator; Ttr, Tachypleus tridentatus. See Additional file 1 for abbreviations of the proteins. [file 1471-2148-12-19-S4.DOC]
