# Supplementary material for: The diversity and evolution of chelicerate hemocyanins
Source: BMC Evol Biol. 2012 Feb 14;12:19. doi: 10.1186/1471-2148-12-19 (PMC3306762; doi:10.1186/1471-2148-12-19)
Supplement: Additional file 5 — Divergence times of chelicerate hemocyanin subunit types (see Figure 2A). Rates across sites were modeled assuming a gamma distribution (Γ) or with a Dirichlet process (D). Divergence time priors were either uniform or modeled with a birth death process. Hard or soft bounds were applied. Divergence times are given in Ma. [file 1471-2148-12-19-S5.DOC]

**Additional file 5.** Divergence times of chelicerate hemocyanin subunit types (see Fig. 2A). Rates across sites were modeled assuming a gamma distribution (**Γ**) or with a Dirichlet process (D). Divergence time priors were either uniform or modeled with a birth death process. Hard or soft bounds were applied. Divergence times are given in Ma.

| **Rates across sites** | **Γ** | **D** | **Γ** | **D** | **Γ** | **D** |
| --- | --- | --- | --- | --- | --- | --- |
| **Divergence time priors** | uniform | uniform | birth death | birth death | birth death | birth death |
| **Bounds** | hard | hard | hard | hard | soft | soft |
|  |  |  |  |  |  |  |
| clade I – (clades II, III, IV) | 539 | 539 | 539 | 539 | 540 | 540 |
| b+c – V+VI | 465 | 465 | 465 | 465 | 445 | 444 |
| b – c | 459 | 459 | 460 | 460 | 437 | 437 |
| V – VI | 182 | 182 | 177 | 174 | 169 | 168 |
| clade II – (clades III + IV) | 537 | 537 | 537 | 537 | 536 | 536 |
| clades III – IV | 515 | 516 | 516 | 516 | 509 | 510 |
| a – II | 472 | 472 | 470 | 470 | 453 | 452 |
| d+f - IIIb + IV | 484 | 483 | 480 | 480 | 467 | 467 |
| d – f | 461 | 461 | 460 | 460 | 441 | 441 |
| IIIb – IV | 302 | 302 | 311 | 313 | 304 | 306 |
| e+g – I+IIIa | 493 | 494 | 496 | 498 | 484 | 489 |
| e – g | 477 | 478 | 480 | 479 | 466 | 467 |
| I – IIIa | 299 | 301 | 318 | 318 | 309 | 316 |
