# Supplementary material for: The diversity and evolution of chelicerate hemocyanins
Source: BMC Evol Biol. 2012 Feb 14;12:19. doi: 10.1186/1471-2148-12-19 (PMC3306762; doi:10.1186/1471-2148-12-19)
Supplement: Additional file 6 — Divergence times of chelicerate taxa, as estimated from the hemocyanin sequences (see Figure 2B). Rates across sites were modeled assuming a gamma distribution (Γ) or with a Dirichlet process (D). Divergence time priors were either uniform or modeled with a birth death process. Hard or soft bounds were applied. Divergence times are given in Ma. [file 1471-2148-12-19-S6.DOC]

**Additional file 6.** Divergence times of chelicerate taxa, as estimated from the hemocyanin sequences (see Fig. 2B). Rates across sites were modeled assuming a gamma distribution (**Γ**) or with a Dirichlet process (D). Divergence time priors were either uniform or modeled with a birth death process. Hard or soft bounds were applied. Divergence times are given in Ma.

| **Rates across sites** | **Γ** | **D** | **Γ** | **D** | **Γ** | **D** |
| --- | --- | --- | --- | --- | --- | --- |
| **Divergence time priors** | uniform | uniform | birth death | birth death | birth death | birth death |
| **Bounds** | hard | hard | hard | hard | soft | soft |
|  |  |  |  |  |  |  |
| Pycnogonida – Euchelicerata | 541 | 541 | 541 | 541 | 543 | 542 |
| Arachnida – Xiphosura | 478 | 479 | 478 | 478 | 462 | 463 |
| Scorpiones – Tetrapulmonata | 442 | 442 | 443 | 443 | 419 | 419 |
| Araneae – Pedipalpi | 386 | 385 | 388 | 387 | 369 | 369 |
| Araneomorpha – Mygalomorpha | 283 | 283 | 282 | 281 | 271 | 270 |
| Uropygi – Amblypygi | 348 | 349 | 353 | 354 | 333 | 333 |
| *L. polyphemus* – (*C. rotundicauda* + T. *tridentatus*) | 69 | 69 | 64 | 64 | 62 | 62 |
| *C. rotundicauda* – *T. tridentatus* | 23 | 23 | 22 | 22 | 21 | 21 |
| *P. imperator* – *A. australis* | 219 | 218 | 225 | 228 | 221 | 221 |
| *N. inaurata* – *C. salei* | 247 | 247 | 248 | 246 | 239 | 240 |
| *E. californicum* – *A. gomesiana* | 34 | 34 | 32 | 32 | 30 | 30 |
